# Supplementary material for: Cyprocide selectively kills nematodes via cytochrome P450 bioactivation
Source: Nat Commun. 2024 Jul 2;15:5529. doi: 10.1038/s41467-024-49738-4 (PMC11219838; doi:10.1038/s41467-024-49738-4)
Supplement: Supplementary file 1 — Supplementary Information [file 41467_2024_49738_MOESM1_ESM.pdf]

SUPPLEMENTARY INFORMATION

Cyprocide Selectively Kills Nematodes via Cytochrome P450 Bioactivation

Authors: Jessica Knox[1,2], Andrew R. Burns[1,2], Brittany Cooke[1,2], Savina R. Cammalleri[1,2], Megan Kitner[3], Justin Ching[4], Jack M.P. Castelli[1,2], Emily Puumala[1], Jamie Snider[2], Emily Koury[5], J.B. Collins[5], Salma Geissah[1,6], James J. Dowling[1,6], Erik C. Andersen[7], Igor Stagljar[1,2,8,9], Leah E. Cowen[1], Mark Lautens[4], Inga Zasada[3] and Peter J. Roy[1,2,10*]

1. Department of Molecular Genetics, University of Toronto, Toronto, ON, Canada

2. Terrence Donnelly Centre for Cellular and Biomolecular Research, University of Toronto, Toronto, ON, Canada

3. United States Department of Agriculture – Agricultural Research Service, Horticultural Crops Disease and Pest Management Research Unit, Corvallis, OR, USA

4. Davenport Research Laboratories, Department of Chemistry, University of Toronto, Toronto, ON, Canada

5. Molecular Biosciences, Northwestern University, Evanston, IL, USA

6. Division of Neurology and Program in Genetics and Genome Biology, The Hospital for Sick Children, Toronto, ON, Canada

7. Biology Department, Johns Hopkins University, Baltimore, MD, USA

8. Department of Biochemistry, University of Toronto, Toronto, ON, Canada

9. Mediterranean Institute for Life Sciences, Meštrovićevo Šetalište 45, HR-21000 Split, Croatia

10. Department of Pharmacology and Toxicology, University of Toronto, Toronto, ON, Canada

Supplementary Information includes:

- Supplementary Methods
- Supplementary Figures 1-58
- Supplementary Tables 1-3

SUPPLEMENTARY METHODS

Synthesis of Cyprocide Analogs

Chemistry-General Considerations

Unless otherwise stated, all reactions were carried out under an atmosphere of dry argon, using glassware that was either oven (120 °C) or flame-dried. Work-up and isolation of compounds was performed using standard benchtop techniques. All commercial reagents were purchased from chemical suppliers (Sigma-Aldrich, Combi-Blocks, or Alfa Aesar) and used without further purification. Dry solvents were obtained using standard procedures (dichloromethane and acetonitrile were distilled over calcium hydride). Togni's reagent I was prepared according to literature procedures[1]. Reactions were monitored using thin-layer chromatography (TLC) on EMD Silica Gel 60 F254 plates. Visualization was performed under UV light (254 nm) or using potassium permanganate ($KMnO_4$) stain. Flash column chromatography was performed on Siliaflash P60 40-63 μm silica gel purchased from Silicycle. NMR characterization data were obtained at 293 K on a Varian Mercury 300 MHz, Varian Mercury 400 MHz, Bruker Advance III 400 MHz, Agilent DD2 500 MHz equipped with a 5 mm Xses cold probe or Agilent DD2 600 MHz. ^{1}H spectra were referenced to the residual solvent signal ($CDCl_3$ = 7.26 ppm, DMSO-d_6 = 2.50 ppm). ^{13}C{^{1}H} spectra were referenced to the residual solvent signal ($CDCl_3$ = 77.16 ppm, DMSO-d_6 = 39.52 ppm). Data for ^{1}H NMR are reported as follows: chemical shift (δ ppm), multiplicity (s = singlet, d = doublet, t = triplet, q = quartet, p = quintet, h = sextet, m = multiplet, br = broad), coupling constant (Hz), integration. NMR spectra were recorded at the University of Toronto Department of Chemistry NMR facility (http://www.chem.utoronto.ca/facilities/nmr/nmr.html). Infrared spectra were recorded on a PerkinElmer Spectrum 100 instrument equipped with a single-bounce diamond/ZnSe ATR accessory in the solid state and are reported in wavenumber (cm^{-1}) units. High resolution mass spectra (HRMS) were obtained on a JEOL JMS-T200GC AccuTOF GCx plus(EI) or an Agilent 6538 UHD Q-TOF (ESI) or a JEOL JMS-100LP AccuTOFLC-plus 4G mass spectrometer equipped with an IONICS® Direct Analysis in Real Time (DART) ion source at the Advanced Instrumentation for Molecular Structure (AIMS) facility of the Department of Chemistry at the University of Toronto (https://sites.chem.utoronto.ca/chemistry/facilities/massspec/about.htm). Melting point ranges were determined on a Fisher-Johns melting point apparatus and are reported uncorrected.

General Procedure A

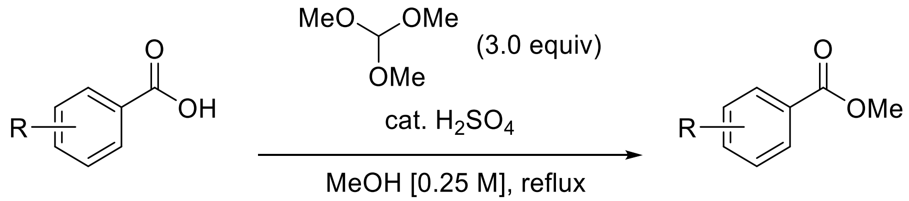

(5.0 mmol) **1a-j**

This reaction was performed in air using glassware directly from the drawer. A round bottom flask equipped with a PTFE-coated magnetic stir bar was charged with carboxylic acid (5.0 mmol, 1.0 equiv). Methanol (20 mL), followed by concentrated sulfuric acid (1 drop per mmol), and trimethyl orthoformate (1.64 mL, 15.0 mmol, 3.0 equiv) were added sequentially at room temperature. The solution was stirred at reflux for 16 h. The solution was cooled to room temperature and concentrated *in vacuo*. The residue was then diluted with ethyl acetate and the organic layer was washed twice with a saturated solution of $NaHCO_3$, water, and brine. The organic layer was dried over $MgSO_4$, filtered, and concentrated *in vacuo* to afford the methyl benzoates (**1a-j**). The crude material was used in the following step without further purification.

General Procedure B

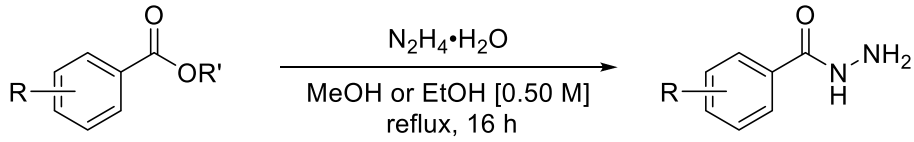

1a-j **2a-j**

This reaction was performed in air using glassware directly from the drawer. A round bottom flask was equipped with a PTFE-coated magnetic stir bar was charged with methyl or ethyl benzoates (5.0 mmol 1.0 equiv). Methanol (10 mL) or ethanol (10 mL) was added, followed by N_2H_4•H_2O (0.20 mL per mmol). The solution was stirred at reflux for 16 h. The solution was cooled to room temperature and concentrated *in vacuo*. The crude solid was recrystallized from methanol or ethanol to afford the aryl hydrazides (**2a-j**).

General Procedure C

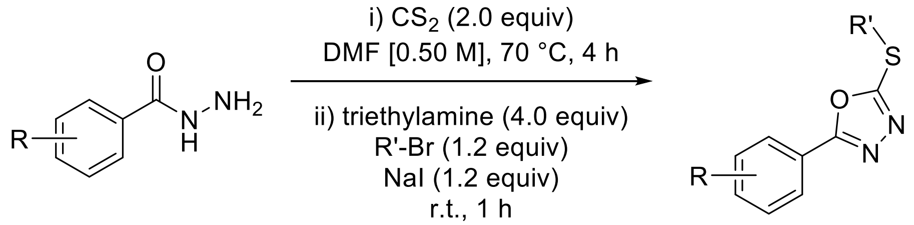

2a-j **3a-k**

A modified literature procedure for the one-pot synthesis of oxadiazoles was followed[2]. A flame-dried 9-dram vial was charged with a PTFE-coated magnetic stir bar and cooled under a positive pressure of argon. To the vial was added hydrazide (1.0 mmol, 1.0 equiv), anhydrous DMF (2.0 mL), followed by CS_2 (0.18 mL, 3.0 mmol, 3.0 equiv). The solution was stirred at 70 ^{o}C for 4 h. The mixture was then cooled to ambient temperature. To the vial were added triethylamine (0.50 mL, 4.0 mmol, 4.0 equiv), alkyl bromide (1.2 mmol, 1.2 equiv), and NaI (0.18 g, 1.2 mmol. 1.2 equiv) sequentially, and the resulting mixture was stirred over night at ambient temperature. The solution was diluted with water and extracted twice with ethyl acetate. The combined organic layers were washed four times with brine, dried over $MgSO_4$, filtered, and concentrated *in vacuo*. The crude material was purified by flash column chromatography with the indicated eluent to afford the alkylated oxadiazoles (**3a-k**).

General Procedure D

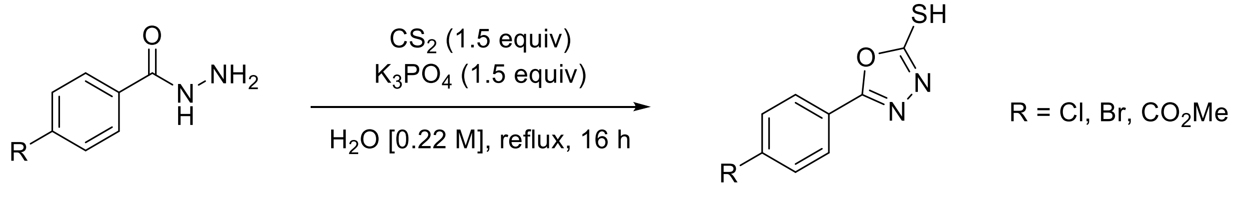

4a-c

A modified literature procedure was followed[3]. This reaction was not performed under inert atmosphere and the glassware was not flame- or oven-dried. A round bottom flask equipped with a PTFE-coated magnetic stir bar was charged with hydrazide (380 mg, 2.2 mmol, 1.0 equiv), K_3PO_4 (709 mg, 3.3 mmol, 1.5 equiv), distilled water (10 mL), and CS_2 (0.20 mL, 3.3 mmol, 1.5 equiv). The solution was stirred at reflux for 16 h. The mixture was allowed to cool to ambient temperature, and 1 M HCl was added dropwise until pH 7, leading to the precipitation of a white solid. The solid was collected by vacuum filtration and washed with ice cold distilled water. The thiols **4a-c** were used as is for future steps.

General Procedure E

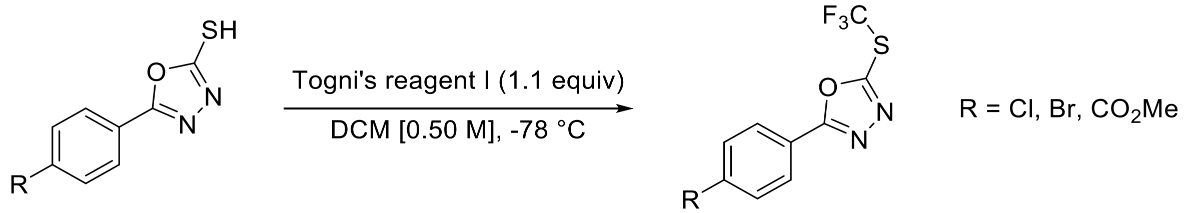

4a-c **3n-p**

A flame-dried 9-dram vial was charged with a PTFE-coated magnetic stir bar and cooled under a positive pressure of argon. To the vial was added thiol (106 mg, 0.5 mmol, 1.0 equiv.) and DCM (1 mL). The solution was cooled to -78 ^{o}C, and Togni's reagent I (263 mg, 0.8 mmol, 1.6 equiv) dissolved in DCM (1 mL) was added dropwise. The mixture was stirred at -78 ^{o}C for three hours and allowed to warm to room temperature. The mixture was concentrated *in vacuo*, and purified by flash column chromatography with the indicated eluent to afford products **3n-p**.

Characterization data for products

2-(ethylthio)-5-(4-(trifluoromethyl)phenyl)-1,3,4-oxadiazole (3a)(cyprocide-V)

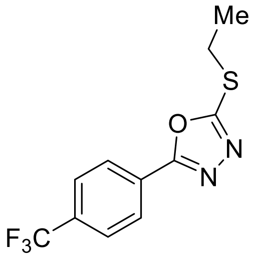

Synthesized according to general procedures A, B, and C from 4-(trifluoromethyl)benzoic acid. The crude material was purified by flash column chromatography (2.5% → 5% EtOAc in pentanes) to afford **3a** as an off-white solid (183 mg, 67% yield from last step), (mp = 76–78 ^{o}C). **^{1}H NMR (500 MHz, CDCl$_3$)** δ 8.13 (dd, *J* = 8.9, 0.8 Hz, 2H), 7.76 (dd, *J* = 8.9, 0.7 Hz, 2H), 3.34 (q, *J* = 7.4 Hz, 3H), 1.53 (t, *J* = 7.4 Hz, 3H). **^{13}C NMR (125 MHz, CDCl$_3$)** δ 165.5, 164.6, 133.3 (q, *J* = 32.9 Hz), 127.1, 126.2 (q, *J* = 3.8 Hz), 124.8, 122.6, 27.2, 14.8. **^{19}F NMR (375 MHz, CDCl$_3$)** δ -63.1. **IR (neat):** 2942, 1622, 1559, 1505, 1469, 1414, 1310, 1201, 1113. **HRMS (DART):** calc for $C_{11}H_{10}N_2OF_3S$ 275.0460 [M+H]$^+$, found 275.0469.

2-(ethylthio)-5-phenyl-1,3,4-oxadiazole (3b)(cyprocide-X)

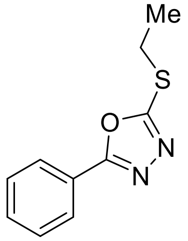

Synthesized according to general procedures B and C from methyl benzoate. The crude material was purified by flash column chromatography (gradient elution, 2.5% → 10% EtOAc in pentanes) to afford **3b** as a white solid (48.2 mg, 23% yield from last step). Characterization data matched that previously reported[4]. **^{1}H NMR (CDCl$_3$, 500 MHz):** δ 8.03 – 7.96 (m, 2H), 7.58 – 7.43 (m, 2H), 3.31 (q, *J* = 7.4 Hz, 1H), 1.51 (t, *J* = 7.4 Hz, 1H). **^{13}C NMR (CDCl$_3$, 125 MHz):** δ 165.8, 164.5, 131.7, 129.1, 126.8, 123.8, 27.2, 14.9.

2-(ethylthio)-5-(3-fluorophenyl)-1,3,4-oxadiazole (3c)(cyprocide-P)

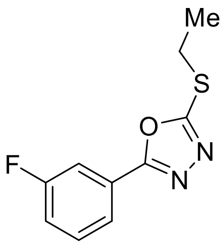

Synthesized according to general procedure C from 3-fluorobenzohydrazide. The crude material was purified by flash column chromatography (gradient elution, 5% → 10% EtOAc in pentanes) to afford **3c** as an off-white solid (98.2 mg, 44% yield from last step). **^{1}H NMR (CDCl$_3$, 500 MHz):** δ 7.80 (ddd, *J* = 7.8, 1.5, 1.0 Hz, 1H), 7.72 – 7.68 (m, 1H), 7.50 – 7.43 (m, 1H), 7.21 (tdd, *J* = 8.4, 2.6, 1.0 Hz, 1H), 3.32 (q, *J* = 7.3 Hz, 2H), 1.52 (t, *J* = 7.4 Hz, 3H). **^{13}C NMR (CDCl$_3$, 125

MHz): δ 165.0, 164.8 (d, $J = 3.4$ Hz), 162.9 (d, $J = 247.7$ Hz), 131.0 (d, $J = 8.1$ Hz), 125.7 (d, $J = 8.6$ Hz), 122.5 (d, $J = 3.3$ Hz), 118.8 (d, $J = 21.3$ Hz), 113.8 (d, $J = 24.4$ Hz), 27.2, 14.8. **^{19}F NMR (375 MHz, $CDCl_3$)** δ -111.2. **IR (neat):** 2925, 1557, 1486, 1458, 1341, 1308, 1267, 1230, 1172. **HRMS (DART):** calc for $C_{10}H_{10}N_2OFS$ 225.0492 $[M+H]^+$, found 225.0494.

2-(ethylthio)-5-(4-nitrophenyl)-1,3,4-oxadiazole (3d)(cyprocide-T)

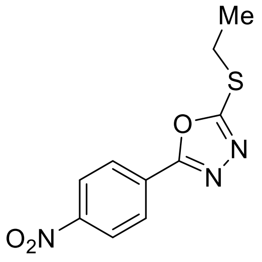

Synthesized according to general procedures B and C from methyl 4-nitrobenzoate. The crude material was purified by flash column chromatography (gradient elution, 5% → 15% EtOAc in pentanes) to afford **3d** as a yellow solid (157 mg, 62% yield from last step), (mp = 98–100 °C). **^{1}H NMR ($CDCl_3$, 500 MHz):** δ 8.41 – 8.32 (m, 2H), 8.23 – 8.16 (m, 2H), 3.36 (q, $J = 7.4$ Hz, 2H), 1.54 (t, $J = 7.4$ Hz, 3H). **^{13}C NMR ($CDCl_3$, 125 MHz):** δ 166.3, 164.0, 149.5, 129.3, 127.6, 124.5, 27.3, 14.8. **IR (neat):** 3108, 1603, 1558, 1513, 1436, 1416, 1361, 1343, 1191. **HRMS (DART):** calc for $C_{10}H_{10}N_3O_3S$ 252.0437 $[M+H]^+$, found 252.0438.

methyl 4-(5-(ethylthio)-1,3,4-oxadiazol-2-yl)benzoate (3e)(cyprocide-U)

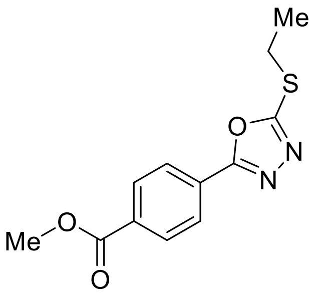

Synthesized according to general procedures B and C from dimethyl terephthalate. General procedure C was modified using iodoethane *in lieu* of bromoethane and NaI. The crude material was purified by flash column chromatography (gradient elution, 5% → 15% EtOAc in pentanes). The material was recrystalized from pentanes to afford **3e** as a white solid (40.2 mg, 15% yield from last step), (mp = 93–95 °C). **^{1}H NMR ($CDCl_3$, 500 MHz):** δ 8.18 – 8.12 (m, 2H), 8.11 – 8.05 (m, 2H), 3.95 (s, 3H), 3.34 (q, $J = 7.4$ Hz, 2H), 1.52 (t, $J = 7.4$ Hz, 3H). **^{13}C NMR ($CDCl_3$, 125 MHz):** δ 166.2, 165.4, 165.0, 132.8, 130.4, 127.6, 126.7, 52.6, 27.2, 14.8. **IR (neat):** 2954, 1724, 1616, 1584, 1498, 1469, 1438, 1410, 1311. **HRMS (DART):** calc for 265.0641 $C_{12}H_{13}N_2O_3S$ $[M+H]^+$, found 265.0646.

2-(2,3-dichlorophenyl)-5-(ethylthio)-1,3,4-oxadiazole (3f)(cyprocide-R)

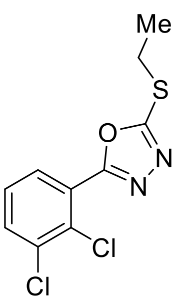

Synthesized according to general procedures A, B, and C from 2,3-dichlorobenzoic acid. The crude material was purified by flash column chromatography (gradient elution, 5% → 10% EtOAc in pentanes) to afford **3f** as an off-white solid (135 mg, 49% yield from last step), (mp = 28–30 °C). **^{1}H NMR ($CDCl_3$, 500 MHz):** δ 7.84 (dd, $J = 7.8$, 1.6 Hz, 1H), 7.63 (dd, $J = 8.1$, 1.6 Hz, 1H), 7.34 (t, $J = 8.0$ Hz, 1H), 3.33 (q, $J = 7.4$ Hz, 2H), 1.53 (t, $J = 7.4$ Hz, 3H). **^{13}C NMR ($CDCl_3$, 125 MHz):** δ 165.6, 163.6, 135.2, 133.2, 131.7, 129.5, 127.7, 125.3,

27.2, 14.9. **IR (neat):** 2961, 1526, 1466, 1458, 1443, 1396, 1263, 1199, 1184. **HRMS (DART):** calc for $C_{10}H_9N_2OSCl_2$ 274.9807 $[M+H]^+$, found 274.9812.

2-(3,4-dichlorophenyl)-5-(ethylthio)-1,3,4-oxadiazole (3g)(cyprocide-S)

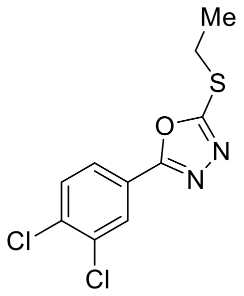

Synthesized according to general procedures A, B, and C from 3,4-dichlorobenzoic acid. The crude material was purified by flash column chromatography (gradient elution, 5% → 10% EtOAc in pentanes) to afford **3g** as a white solid (138 mg, 50% yield from last step), (mp = 63–65 °C). **^{1}H NMR ($CDCl_3$, 500 MHz):** δ 8.09 (d, *J* = 2.0 Hz, 1H), 7.84 (dd, *J* = 8.4, 2.0 Hz, 1H), 7.57 (d, *J* = 8.4 Hz, 1H), 3.33 (q, *J* = 7.4 Hz, 2H), 1.52 (t, *J* = 7.4 Hz, 3H). **^{13}C NMR ($CDCl_3$, 125 MHz):** δ 165.3, 164.0, 136.2, 133.8, 131.4, 128.4, 125.7, 123.6, 27.2, 14.8. **IR (neat):** 3087, 1602, 1542, 1454, 1393, 1374, 1270, 1256, 1188. **HRMS (DART):** calc for $C_{10}H_9N_2OSCl_2$ 274.9807 $[M+H]^+$, found 274.9812.

2-(5-chloropyridin-2-yl)-5-(ethylthio)-1,3,4-oxadiazole (3h)(cyprocide-Z)

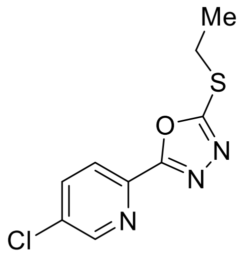

Synthesized according to general procedures A, B, and C from 5-chloropicolinic acid. The crude material was purified by flash column chromatography (gradient elution, 5% → 10% EtOAc in pentanes) to afford **3h** as an off-white solid (116 mg, 48% yield from last step), (mp = 63–65 °C). **^{1}H NMR ($CDCl_3$, 500 MHz):** δ 8.68 (dd, *J* = 2.4, 0.8 Hz, 1H), 8.14 (dd, *J* = 8.5, 0.7 Hz, 1H), 7.84 (dd, *J* = 8.5, 2.4 Hz, 1H), 3.34 (q, *J* = 7.4 Hz, 2H), 1.51 (t, *J* = 7.4 Hz, 3H). **^{13}C NMR ($CDCl_3$, 125 MHz):** δ 166.4, 164.2, 149.4, 141.5, 137.2, 134.5, 123.6, 27.2, 14.7. **IR (neat):** 3047, 2968, 2927, 1563, 1538, 1472, 1455, 1418, 1112. **HRMS (DART):** calc for $C_9H_9N_3OSCl$ 242.0149 $[M+H]^+$, found 242.0156.

2-(3-chlorophenyl)-5-(ethylthio)-1,3,4-oxadiazole (3i)(cyprocide-O)

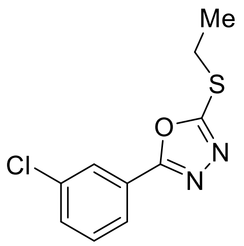

Synthesized according to general procedures B, and C from ethyl 3-chlorobenzoate. The crude material was purified by flash column chromatography (gradient elution, 2.5% → 10% EtOAc in pentanes) to afford **3i** as an off-white solid (147 mg, 61% yield from last step), (mp = 40–42 °C). **^{1}H NMR ($CDCl_3$, 500 MHz):** δ 7.98 (ddd, *J* = 2.1, 1.7, 0.5 Hz, 1H), 7.89 (ddd, *J* = 7.7, 1.7, 1.2 Hz, 1H), 7.48 (ddd, *J* = 8.1, 2.1, 1.2 Hz, 1H), 7.43 (ddd, *J* = 8.1, 7.7, 0.5 Hz, 1H), 3.32 (q, *J* = 7.4 Hz, 2H), 1.52 (t, *J* = 7.4 Hz, 3H). **^{13}C NMR ($CDCl_3$, 125 MHz):** δ 165.1, 164.6, 135.3, 131.7, 130.5, 126.7, 125.4, 124.8, 27.2, 14.8. **IR (neat):** 2964, 2927, 2867, 1553, 1352, 1412, 1175, 1088, 1065. **HRMS (DART):** calc for $C_{10}H_{10}N_2OSCl$ 241.0197 $[M+H]^+$, found 241.0208.

2-(4-chlorophenyl)-5-((2,2,2-trifluoroethyl)thio)-1,3,4-oxadiazole (3j)(cyprocide-G)

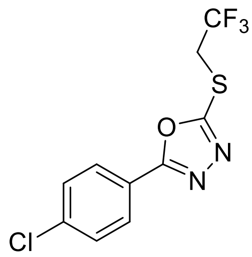

Synthesized according to general procedures B and C from methyl 4-chlorobenzoate. General procedure C was modified using alkyl iodide 1,1,1-trifluoro-2-iodoethane *in lieu* of alkyl bromide and NaI. The crude material was purified by flash column chromatography (gradient elution, 5% → 10% EtOAc in pentanes). The material was recrystallized from pentanes to afford **3j** as a beige solid (30.3 mg, 10% yield from last step), (mp = 77–79 °C). **^{1}H NMR ($CDCl_3$, 500 MHz):** δ 7.97 – 7.93 (m, 2H), 7.52 – 7.47 (m, 2H), 3.99 (q, J = 9.3 Hz, 2H). **^{13}C NMR ($CDCl_3$, 125 MHz):** δ 166.0, 161.8, 138.5, 129.7, 128.2, 124.3 (q, J = 276.7 Hz), 121.8, 34.4 (q, J = 35.3 Hz). **^{19}F NMR (375 MHz, $CDCl_3$)** δ -66.9. **IR (neat):** 3009, 2957, 1608, 1553, 1476, 1316, 1279, 1180, 1125. **HRMS (DART):** calc for $C_{10}H_7N_2OF_3SCl$ 294.9914 [M+H]$^+$, found 294.9918.

Synthesis of 2-(4-chlorophenyl)-5-(propylthio)-1,3,4-oxadiazole (3k)

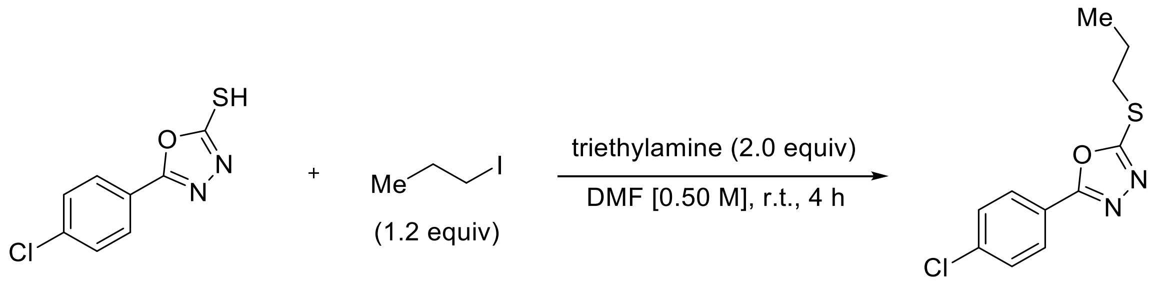

4a **3k**

2-(4-chlorophenyl)-5-(propylthio)-1,3,4-oxadiazole (3k)(cyprocide-J)

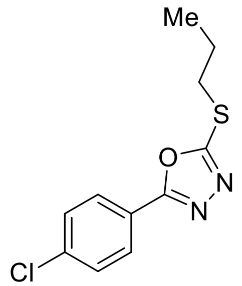

A flame-dried 9-dram vial was charged with a PTFE-coated magnetic stir bar and cooled under a positive pressure of argon. To the vial were added thiol **4** (149 mg, 0.7 mmol, 1.0 equiv.), DMF (1.4 mL), 1-iodopropane (82 µL, 0.84 mmol, 1.2 equiv.), and triethylamine (0.20 mL, 1.4 mmol, 2.0 equiv), and the resulting mixture was stirred at ambient temperature for 4 h. The solution was diluted with water and extracted twice with ethyl acetate. The combined organic layers were washed four times with brine, dried over $MgSO_4$, filtered, and concentrated *in vacuo*. The crude material was purified by flash column chromatography (gradient elution, 2.5% → 10% EtOAc in pentanes) to afford **3k** as an off-white solid (150 mg, 59% yield), (mp = 55–57 °C). **^{1}H NMR ($CDCl_3$, 500 MHz):** δ 7.95 – 7.92 (m, 2H), 7.49 – 7.44 (m, 2H), 3.28 (dd, J = 7.5, 6.9 Hz, 2H), 1.88 (h, J = 7.4 Hz, 2H), 1.08 (t, J = 7.4 Hz, 3H). **^{13}C NMR ($CDCl_3$, 125 MHz):** δ 165.0, 165.0, 137.9, 129.5, 128.0, 122.3, 34.7, 22.8, 13.3. **IR (neat):** 2952,

2927, 2867, 1607, 1487, 1460, 1412, 1368, 1125. **HRMS (DART):** calc for $C_{11}H_{12}N_2OSCl$ 255.0353 $[M+H]^+$, found 255.0369.

Synthesis of 2-(4-chlorophenyl)-5-(ethylsulfinyl)-1,3,4-oxadiazole (3l)

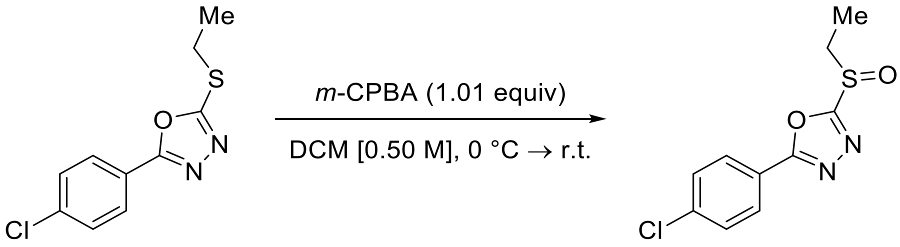

Cyprocide-B **3l**

2-(4-chlorophenyl)-5-(ethylsulfinyl)-1,3,4-oxadiazole (3l)(cyprocide-K)

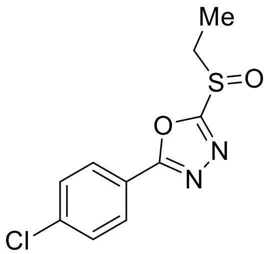

Cyprocide-B was prepared according to general procedures B and C from methyl 4-chlorobenzoate. A flame-dried 9-dram vial was charged with a PTFE-coated magnetic stir bar and cooled under a positive pressure of argon. To the vial was added **Cyprocide-B** (122 mg, 0.51 mmol, 1.0 equiv) and DCM (5mL). The solution was cooled to 0 °C and *m*-CPBA (89.7 mg, 0.52 mmol, 1.01 equiv) was added portionwise. The mixture was then stirred at 0 °C for 2 h and then warmed to ambient temperature. The mixture was diluted with DCM, washed twice with a saturated aqueous solution of $NaHCO_3$ and once with brine. The organic layer was dried over $MgSO_4$, filtered, and concentrated *in vacuo*. The crude material was purified by flash column chromatography (gradient elution, 30% → 35% EtOAc in pentanes) to afford **3l** as a white solid (84.8 mg, 65% yield), (mp = 56–58 °C). **^{1}H NMR ($CDCl_3$, 500 MHz):** δ 8.11 – 8.01 (m, 2H), 7.57 – 7.50 (m, 2H), 3.47 (qd, *J* = 7.4, 2.1 Hz, 2H), 1.48 (t, *J* = 7.5 Hz, 3H). **^{13}C NMR ($CDCl_3$, 125 MHz):** δ 166.7, 165.9, 139.4, 129.9, 128.9, 121.2, 47.7, 6.4. **IR (neat):** 2934, 1600, 1544, 1478, 1470, 1403, 1185, 1116, 1089. **HRMS (DART):** calc for $C_{10}H_{10}N_2O_2SCl$ 257.0146$[M+H]^+$, found 257.0139.

Synthesis of 2-(4-chlorophenyl)-5-(ethylsulfonyl)-1,3,4-oxadiazole (3m)

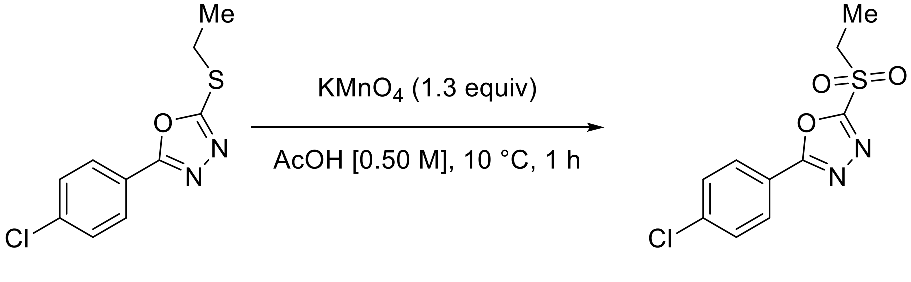

Cyprocide-B **3m**

2-(4-chlorophenyl)-5-(ethylsulfonyl)-1,3,4-oxadiazole (3m)(cyprocide-L)

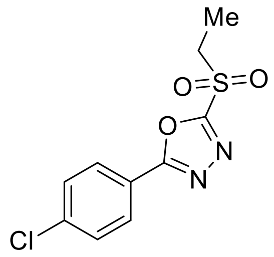

Cyprocide-B was prepared according to general procedures B and C from methyl 4-chlorobenzoate. A modified literature procedure was followed for the oxidation of sulfide to sulfone[4]. A flame-dried 9-dram vial was charged with a PTFE-coated magnetic stir bar and cooled under a positive pressure of argon. To the vial was added **Cyprocide-B** (96.3 g, 0.51 mmol, 1.0 equiv) and glacial acetic acid (1mL). The solution was cooled to 10 °C and $KMnO_4$ (82.2 mg, 0.52 mmol, 1.3 equiv) was added as a 5% aqueous solution over 10 min. The mixture was then stirred at 10 °C for 1 h and then warmed to ambient temperature after which a 40% $NaHSO_3$ solution was added until the colour dissipated. The mixture was extracted with twice with ethyl acetate and the combined organic layers were washed with brine, dried over $MgSO_4$, filtered, and concentrated *in vacuo*. The crude material was purified by flash column chromatography (gradient elution, 10% → 20% EtOAc in pentanes) to afford **3m** as a white solid (72.1 mg, 66% yield). Characterization data matched that previously reported[4]. **^{1}H NMR ($CDCl_3$, 500 MHz):** δ 8.17 – 7.99 (m, 2H), 7.59 – 7.48 (m, 2H), 3.62 (q, *J* = 7.4 Hz, 2H), 1.55 (t, *J* = 7.4 Hz, 3H). **^{13}C NMR ($CDCl_3$, 125 MHz):** δ 166.0, 161.5, 140.0, 130.0, 129.1, 120.7, 50.2, 7.0.

2-(4-chlorophenyl)-5-((trifluoromethyl)thio)-1,3,4-oxadiazole (3n)(cyprocide-I)

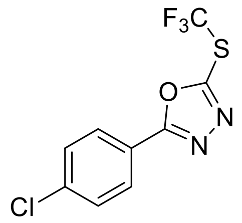

Synthesized according to general procedures A, B, E, and D from methyl 4-chlorobenzoate. The crude material was purified by flash column chromatography (gradient elution, 0% → 10% EtOAc in pentanes). The material was recrystallized from pentanes to afford the product **3n** as a white solid (33.1 mg, 24% yield), (mp = 27–29 °C). **^{1}H NMR ($CDCl_3$, 500 MHz):** δ 8.07 – 7.99 (m, 2H), 7.58 – 7.50 (m, 2H). **^{13}C NMR ($CDCl_3$, 125 MHz):** δ 167.9, 154.3 (q, *J* = 3.3 Hz), 139.3, 129.7, 128.5, 127.1 (q, *J* = 313.3 Hz), 121.2. **^{19}F NMR (375 MHz, $CDCl_3$)** δ -38.7. **IR (neat):** 3090, 2918, 1603, 1544, 1479, 1407, 1299, 1280, 1148. **HRMS (DART):** calc for $C_9H_5N_2OF_3SCl$ 280.9758 $[M+H]^+$, found 280.9768.

2-(4-bromophenyl)-5-((trifluoromethyl)thio)-1,3,4-oxadiazole (3o)(cyprocide-N-2)

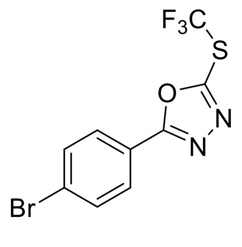

Synthesized according to general procedures A, B, E, and D from methyl 4-bromobenzoate. The crude material was purified by flash column chromatography (gradient elution, 2.5% → 10% EtOAc in pentanes). The material was recrystallized from pentanes to afford the product **3o** as a white solid (20.6 mg, 13% yield), (mp = 48–50 °C). **^{1}H NMR ($CDCl_3$, 500 MHz):** δ 7.98 – 7.93 (m, 2H), 7.73 – 7.68 (m, 2H). **^{13}C NMR ($CDCl_3$, 125 MHz):** δ 168.1, 154.5

(q, J = 3.2 Hz), 132.9, 128.8, 127.9, 127.2 (q, J = 312.6 Hz), 121.8. **^{19}F NMR (375 MHz, $CDCl_3$)** δ -38.7. **IR (neat):** 3087, 1599, 1543, 1478, 1403, 1278, 1148, 1092, 1068. **HRMS (DART):** calc for $C_9H_5N_2OF_3SBr$ 324.9253 $[M+H]^+$, found 324.9259.

methyl 4-(5-((trifluoromethyl)thio)-1,3,4-oxadiazol-2-yl)benzoate (3p)(cyprocide-U-2)

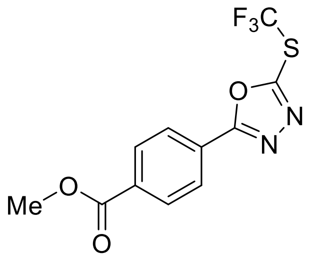

Synthesized according to general procedures A, B, E, and D from dimethyl terephthalate. The crude material was purified by flash column chromatography (gradient elution, 2.5% → 10% EtOAc in pentanes). The material was recrystallized from pentanes to afford the product **3p** as a white solid (33.1 mg, 24% yield), (mp = 61–63 °C). **^{1}H NMR ($CDCl_3$, 500 MHz):** δ 8.24 – 8.19 (m, 2H), 8.19 – 8.15 (m, 2H), 3.97 (s, 3H). **^{13}C NMR ($CDCl_3$, 125 MHz):** δ 168.0, 166.0, 154.9 (q, J = 3.3 Hz), 134.0, 130.6, 127.4, 127.2 (q, J = 312.9 Hz), 126.6, 52.8. **^{19}F NMR (375 MHz, $CDCl_3$)** δ -38.6. **IR (neat):** 2968, 1718, 1545, 1478, 1446, 1417, 1279, 1158, 1105. **HRMS (DART):** calc for $C_{11}H_8N_2O_3F_3S$ 305.0200 $[M+H]^+$, found 305.0198.

HRMS data of products **3a**, **3c-3l**, and **3n-3p** are displayed in Supplementary Figs. 7-20. The NMR spectra of products **3a-3p** are displayed in Supplementary Figs. 21-58.

SUPPLEMENTARY FIGURES

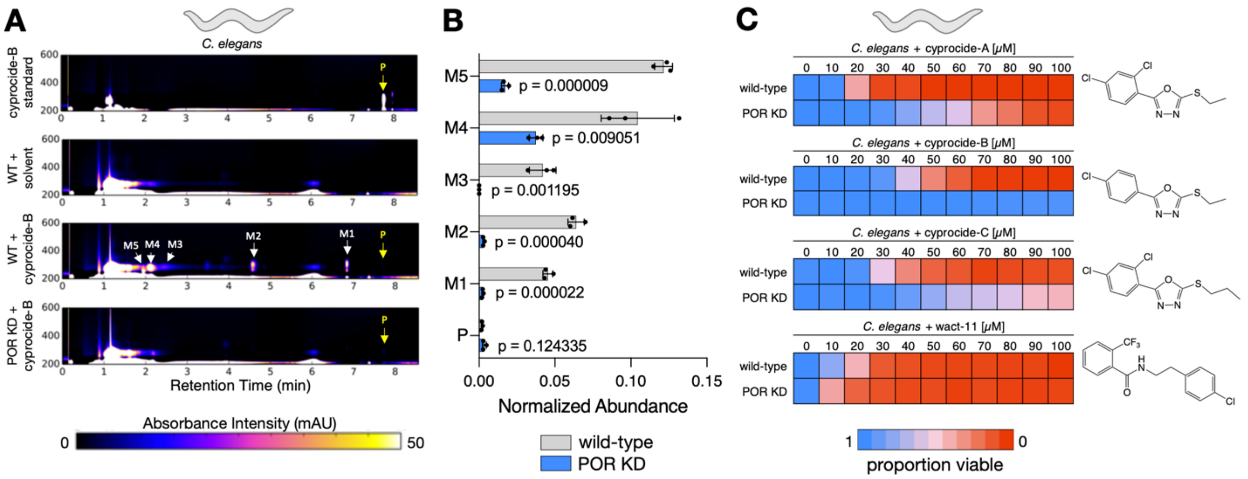

Supplementary Figure 1. The Cyprocides are metabolically bioactivated by P450s in *C. elegans.* (**A**) HPLC chromatograms of *C. elegans* lysates after six-hour incubation with 100 μM cyprocide-B. Cyprocide-B is transformed into five metabolites visible by HPLC-DAD. Peaks corresponding to unmodified cyprocide-B parent (P) and metabolites (M1-M5) are indicated in wild-type (WT) and POR knockdown (POR KD) conditions. The Y-axis indicates absorbance wavelength in nm. (**B**) HPLC-DAD quantification of parent and metabolite abundance from (A) in WT and POR KD conditions (n=3 biological replicates (BRs)). Error bars represent standard deviation, p-values were obtained from unpaired two-tailed Student's t-tests comparing the mean normalized abundance in WT and POR KD conditions (**C**) Viability of adult *C. elegans* wild-type or POR KD worms after a 48-hour incubation in each chemical exposure condition is indicated by the colour-coded scale (n=3 BRs). Structures of assayed compounds are shown. Source data are provided as a Source Data file.

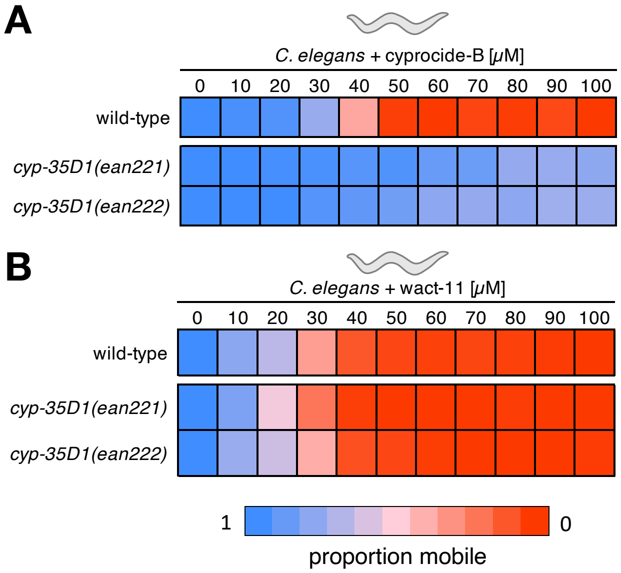

Supplementary Figure 2. *C. elegans* CYP-35D1 is required for cyprocide-B-induced lethality. Adult *C. elegans* wild-type (N2), *cyp-35D1(ean221)*, and *cyp-35D1(ean222)* deletion mutants were exposed to the indicated concentrations of cyprocide-B (**A**) or wact-11 (**B**) for four days. The average proportion of mobile worms in each condition at the endpoint of the assay is indicated by the colour coded scale (n=3 BRs). Source data are provided as a Source Data file.

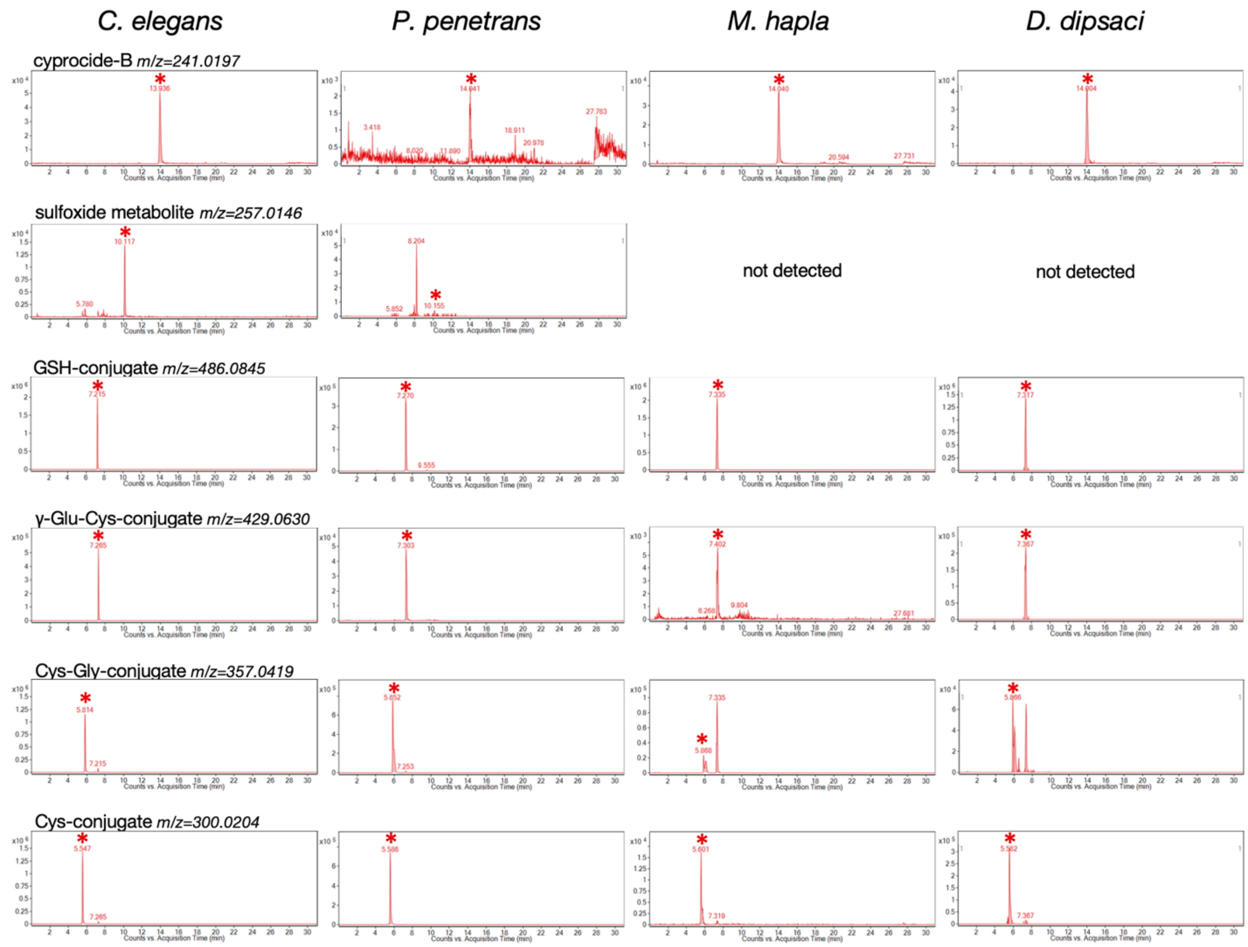

Supplementary Figure 3. Cyprocide-B is metabolized in diverse nematode species. Nematode lysates were analysed by LC-MS after a six-hour (*C. elegans*), 16-hour (*P. penetrans* and *M. hapla* J2), or 24-hour (*D. dipsaci*) incubation with 100 μM cyprocide-B. EICs for the indicated m/z values of the cyprocide-B parent and metabolites are shown for each species. Asterisks indicate the analyte peak at the retention time of interest. Analyte peaks with an abundance below the threshold of $1x10^3$ counts were considered 'not detected'. Source data are provided as a Source Data file.

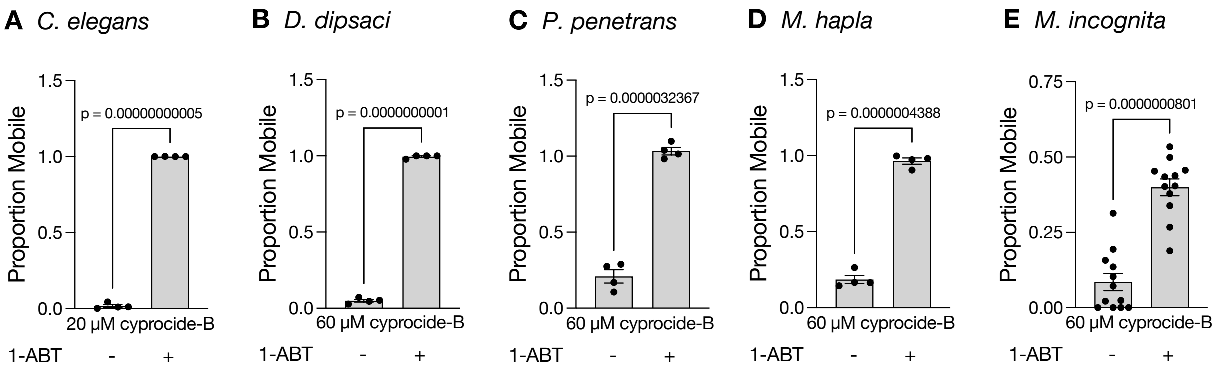

Supplementary Figure 4. 1-ABT suppresses cyprocide-B activity in *C. elegans* and PPNs. Preincubation with 1 mM of the non-specific, irreversible P450 inhibitor 1-ABT suppresses the activity of cyprocide-B in *C. elegans* L1s (n=4 BRs) (**A**), *D. dipsaci* (n=4 BRs) (**B**), *P. penetrans* (n=4 BRs) (**C**), *M. hapla* J2s (n=4 BRs) (**D**), and *M. incognita* J2s (n=12 technical replicates (TRs) from 2 BRs) (**E**). The proportion of mobile worms after chemical exposure is reported (see methods for details). Error bars represent SEM, p-values were obtained from unpaired two-tailed Student's t-tests comparing the mean proportion mobile in conditions with and without 1-ABT. Source data are provided as a Source Data file.

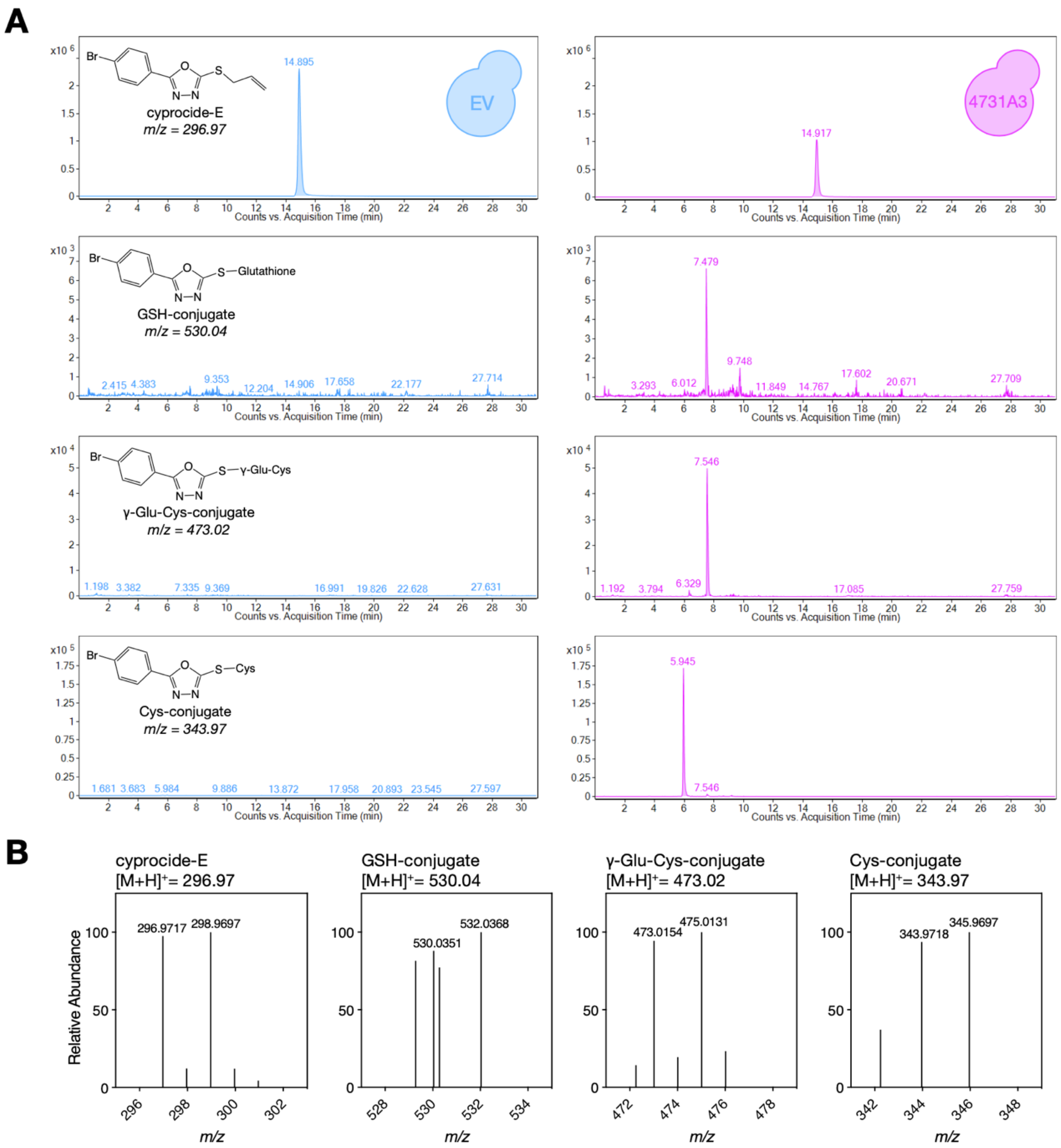

Supplementary Figure 5. LMW thiol conjugates of cyprocide-E are produced in yeast expressing *M. incognita* CYP4731A3. Yeast expressing *Mi*-CYP4731A3 and the empty vector (EV) control strain were exposed to 100 μM cyprocide-E for six hours, lysed, and analyzed by LC-MS. (**A**) Extracted ion chromatograms for the indicated cyprocide-E parent and thiol conjugate m/z values are shown for both EV (left) and CYP4731A3-expressing (right) strains. (**B**) Mass spectra from cyprocide-E-exposed CYP4731A3-expressing yeast lysates highlighting the cyprocide-E parent and thiol conjugate masses. Source data are provided as a Source Data file.

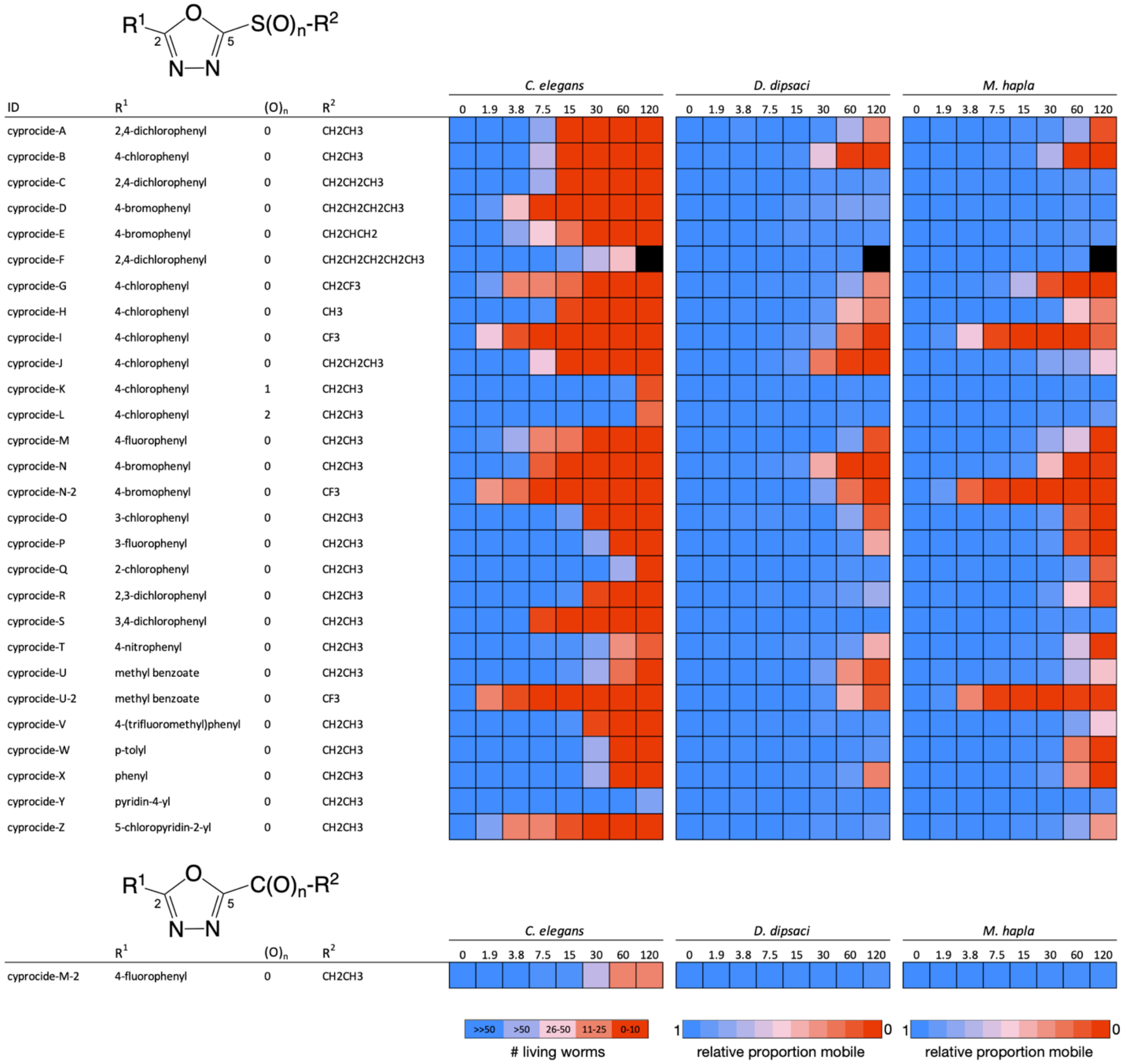

Supplementary Figure 6. SAR analysis identifies cyprocide-B analogs with increased potency in *C. elegans* and PPNs. 29 Cyprocides with modifications in the electron withdrawing group (R^1), leaving group (R^2), or oxidation state $(O)_n$, were tested in dose-response analyses against *C. elegans, D. dipsaci* and *M. hapla*. In *C. elegans* the number of living worms after five days of compound exposure is indicated by the colour-coded scale. In *D. dipsaci* and *M. hapla* the proportion of mobile worms in each condition relative to the DMSO solvent control after five days of compound exposure is indicated by the colour-coded scale. Black indicates that the condition was not tested. These summary data are the mean of two biological replicates (n=3 TRs for each). Source data are provided as a Source Data file.

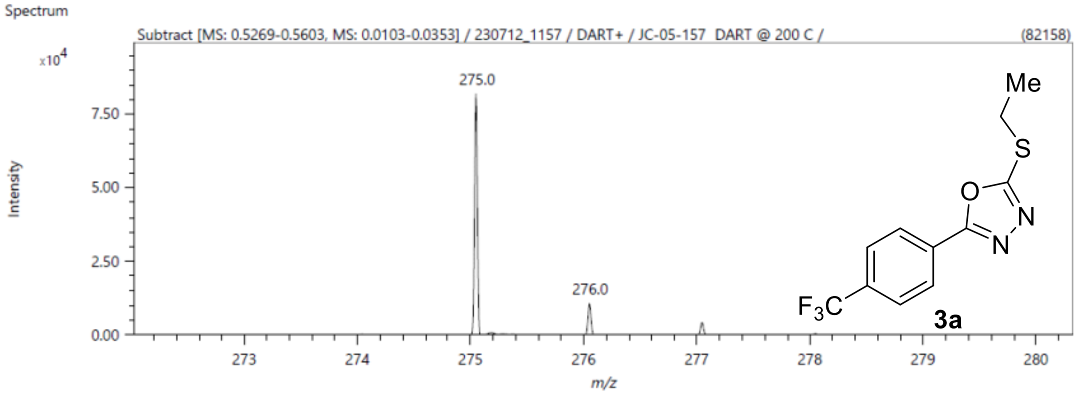

Elemental Composition

Parameters

			Elements Set 1:						
Tolerance:	±10.00 mDa		Symbol	C	H	O	N	S	F
Electron:	Even		Min	0	0	0	0	1	3
Charge:	+1		Max	100	200	20	10	1	3
DBE:	-1.5 - 100.0								

Results

Mass	Intensity	Formula	Calculated Mass	Mass Difference [mDa]	Mass Difference [ppm]	DBE
275.04686	82157.92	C11 H10 N2 O F3 S	275.04604	0.81	2.95	6.5
		H10 N8 O4 F3 S	275.04923	-2.38	-8.64	-1.5
		C6 H10 N4 O3 F3 S	275.04202	4.84	17.58	2.5
		C5 H10 N6 O2 F3 S	275.05326	-6.40	-23.26	2.5
		C2 H6 N10 O F3 S	275.03934	7.52	27.34	3.5
		C H10 N6 O5 F3 S	275.03800	8.86	32.21	-1.5

Supplementary Figure 7. HRMS data of compound 3a.

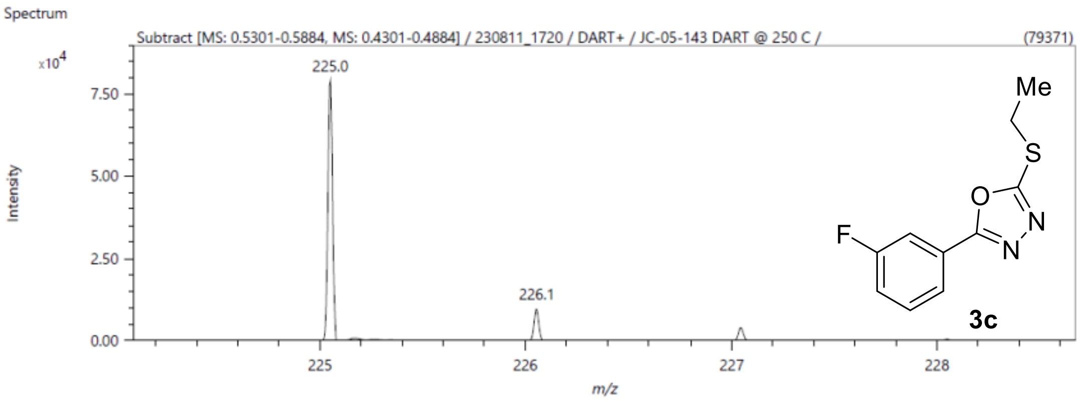

Elemental Composition

Parameters		Elements Set 1:						
Tolerance:	±10.00 mDa	Symbol	C	H	O	N	F	S
Electron:	Even	Min	0	0	0	0	0	0
Charge:	+1	Max	100	200	20	10	1	1
DBE:	-1.5 - 100.0							

Results

Mass	Intensity	Formula	Calculated Mass	Mass Difference [mDa]	Mass Difference [ppm]	DBE
225.04942	79370.66	C10 H10 N2 O F S	225.04924	0.18	0.82	6.5
		C2 H6 N8 O4 F	225.04906	0.37	1.63	3.5
		C9 H9 N2 O5	225.05060	-1.18	-5.23	6.5
		C13 H9 N2 S	225.04810	1.33	5.89	10.5
		C5 H5 N8 O3	225.04791	1.51	6.71	7.5
		C H10 N4 O8 F	225.04772	1.70	7.57	-1.5

Supplementary Figure 8. HRMS data of compound 3c.

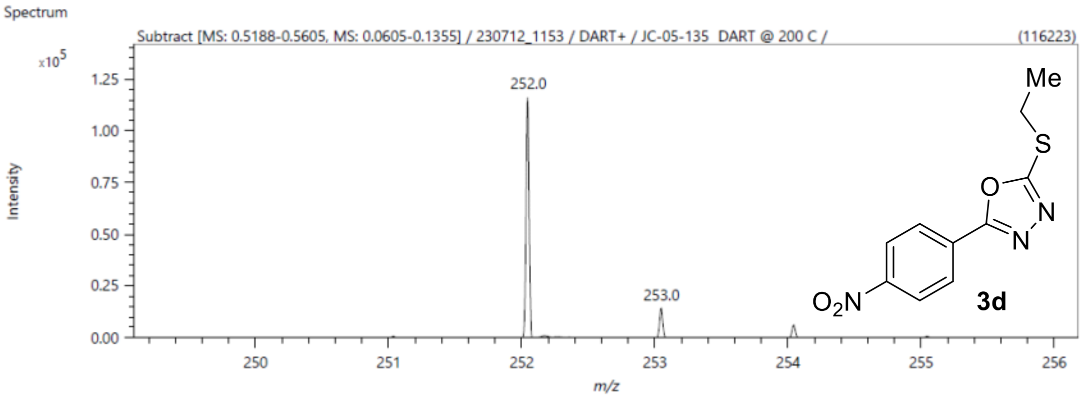

Elemental Composition

Parameters		Elements Set 1:					
Tolerance:	±10.00 mDa	Symbol	C	H	O	N	S
Electron:	Even	Min	0	0	0	0	1
Charge:	+1	Max	100	200	20	10	1
DBE:	-1.5 - 100.0						

Results

Mass	Intensity	Formula	Calculated Mass	Mass Difference [mDa]	Mass Difference [ppm]	DBE
252.04375	116223.49	C10 H10 N3 O3 S	252.04374	0.01	0.04	7.5
		C6 H6 N9 O S	252.04105	2.70	10.70	8.5
		C15 H10 N O S	252.04776	-4.01	-15.92	11.5
		C5 H10 N5 O5 S	252.03972	4.03	16.00	3.5
		C4 H14 N O9 S	252.03838	5.37	21.31	-1.5
		C3 H14 N3 O8 S	252.04961	-5.86	-23.26	-1.5

Supplementary Figure 9. HRMS data of compound 3d.

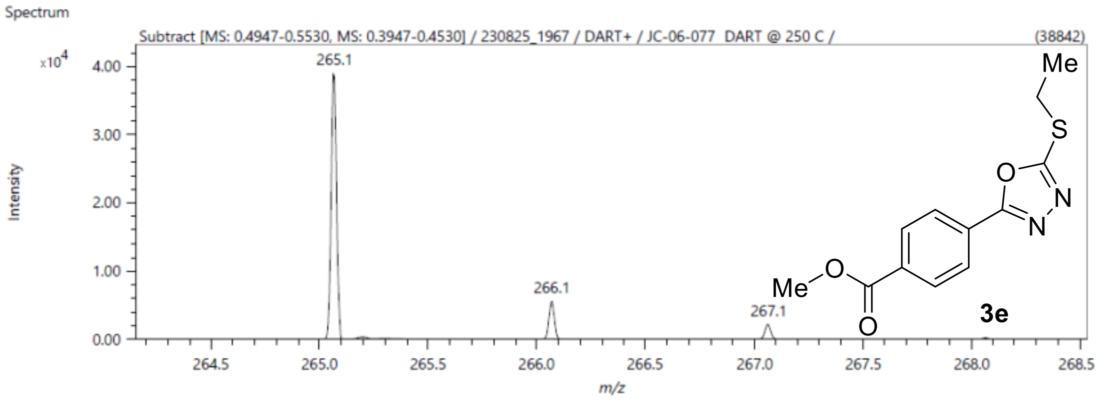

Elemental Composition

Parameters		Elements Set 1:						
Tolerance:	±10.00 mDa	Symbol	C	H	O	N	S	Cl
Electron:	Even	Min	0	0	0	0	0	0
Charge:	+1	Max	100	200	10	10	1	0
DBE:	-1.5 - 100.0							

Results

Mass	Intensity	Formula	Calculated Mass	Mass Difference [mDa]	Mass Difference [ppm]	DBE
265.06463	38841.68	C20 H9 O	265.06479	-0.17	-0.62	16.5
		C12 H13 N2 O3 S	265.06414	0.49	1.84	7.5
		C4 H9 N8 O6	265.06396	0.67	2.53	4.5
		C3 H13 N4 O10	265.06262	2.01	7.57	-0.5
		C8 H13 N2 O8	265.06664	-2.02	-7.60	3.5
		C H13 N8 O6 S	265.06733	-2.70	-10.19	-0.5

Supplementary Figure 10. HRMS data of compound 3e.

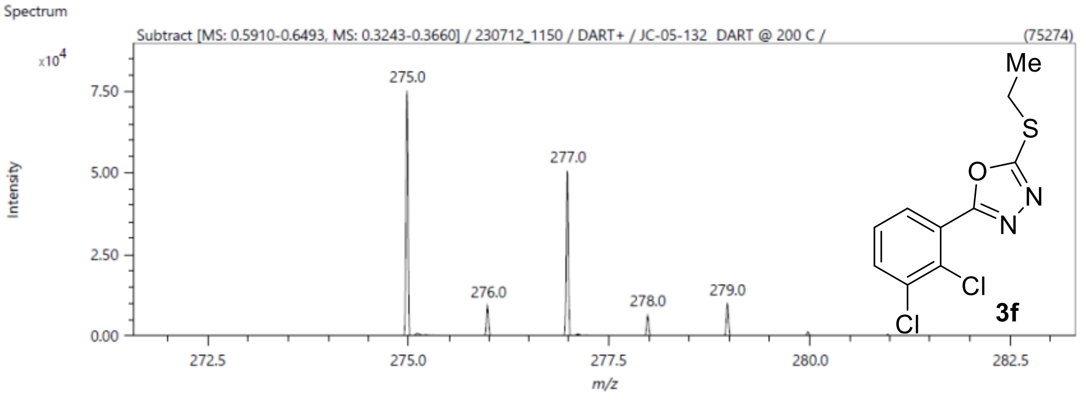

Elemental Composition

Parameters		Elements Set 1:						
Tolerance:	±10.00 mDa	Symbol	C	H	O	N	S	Cl
Electron:	Even	Min	0	0	0	0	1	2
Charge:	+1	Max	100	200	20	10	1	2
DBE:	-1.5 - 100.0							

Results

Mass	Intensity	Formula	Calculated Mass	Mass Difference [mDa]	Mass Difference [ppm]	DBE
274.98119	75274.42	C10 H9 N2 O S Cl2	274.98072	0.48	1.74	6.5
		C5 H9 N4 O3 S Cl2	274.97669	4.50	16.37	2.5
		C4 H9 N6 O2 S Cl2	274.98793	-6.73	-24.48	2.5
		C H5 N10 O S Cl2	274.97401	7.19	26.14	3.5
		H9 N6 O5 S Cl2	274.97267	8.52	31.00	-1.5
		C8 H13 O4 S Cl2	274.99061	-9.42	-34.24	1.5

Supplementary Figure 11. HRMS data of compound 3f.

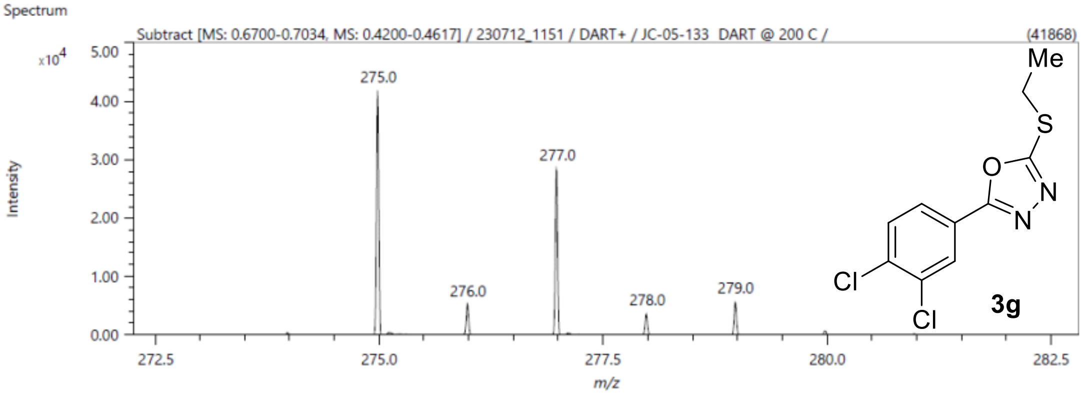

Elemental Composition

Parameters		Elements Set 1:						
Tolerance:	±10.00 mDa	Symbol	C	H	O	N	S	Cl
Electron:	Even	Min	0	0	0	0	1	2
Charge:	+1	Max	100	200	20	10	1	2
DBE:	-1.5 - 100.0							

Results

Mass	Intensity	Formula	Calculated Mass	Mass Difference [mDa]	Mass Difference [ppm]	DBE
274.98129	41867.61	C10 H9 N2 O S Cl2	274.98072	0.58	2.09	6.5
		C5 H9 N4 O3 S Cl2	274.97669	4.60	16.72	2.5
		C4 H9 N6 O2 S Cl2	274.98793	-6.64	-24.13	2.5
		C H5 N10 O S Cl2	274.97401	7.28	26.49	3.5
		H9 N6 O5 S Cl2	274.97267	8.62	31.35	-1.5
		C8 H13 O4 S Cl2	274.99061	-9.32	-33.90	1.5

Supplementary Figure 12. HRMS data of compound 3g.

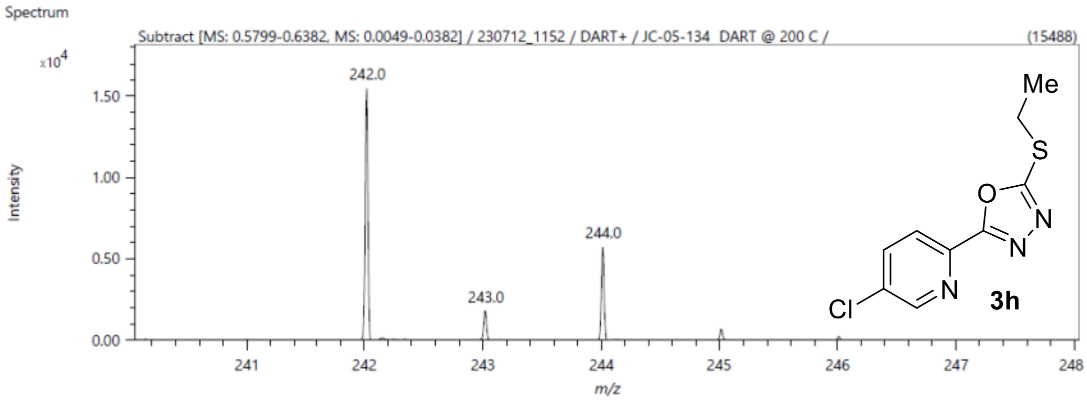

Elemental Composition

Parameters	
Tolerance:	±10.00 mDa
Electron:	Even
Charge:	+1
DBE:	-1.5 - 100.0

Elements Set 1:

Symbol	C	H	O	N	S	Cl
Min	0	0	0	0	1	1
Max	100	200	20	10	1	1

Results

Mass	Intensity	Formula	Calculated Mass	Mass Difference [mDa]	Mass Difference [ppm]	DBE
242.01557	15488.00	C9 H9 N3 O S Cl	242.01494	0.64	2.63	6.5
		C4 H9 N5 O3 S Cl	242.01091	4.66	19.25	2.5
		C3 H9 N7 O2 S Cl	242.02215	-6.57	-27.17	2.5
		C7 H13 N O4 S Cl	242.02483	-9.26	-38.26	1.5

Supplementary Figure 13. HRMS data of compound 3h.

Spectrum

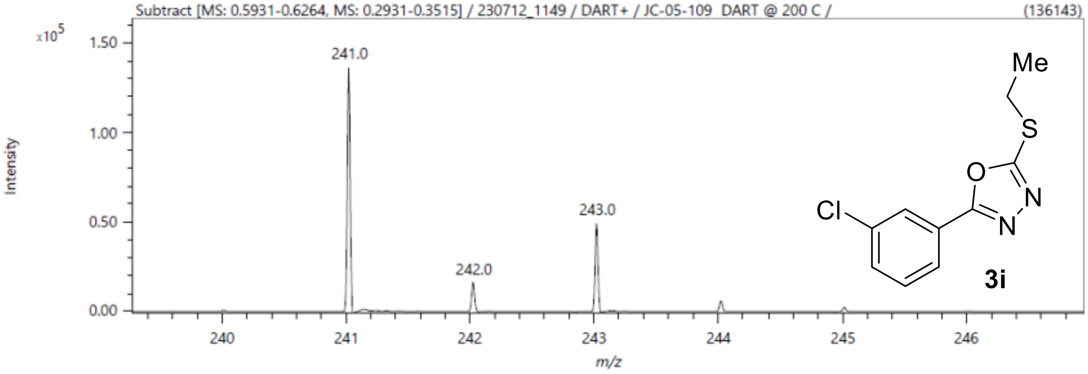

Elemental Composition

Parameters		Elements Set 1:						
Tolerance:	±10.00 mDa	Symbol	C	H	O	N	S	Cl
Electron:	Even	Min	0	0	0	0	1	1
Charge:	+1	Max	100	200	20	10	1	1
DBE:	-1.5 - 100.0							

Results

Mass	Intensity	Formula	Calculated Mass	Mass Difference [mDa]	Mass Difference [ppm]	DBE
241.02082	136142.61	C10 H10 N2 O S Cl	241.01969	1.13	4.68	6.5
		C5 H10 N4 O3 S Cl	241.01567	5.15	21.37	2.5
		C4 H10 N6 O2 S Cl	241.02690	-6.08	-25.23	2.5
		C H6 N10 O S Cl	241.01298	7.84	32.51	3.5
		C8 H14 O4 S Cl	241.02958	-8.77	-36.38	1.5
		H10 N6 O5 S Cl	241.01164	9.17	38.06	-1.5

Supplementary Figure 14. HRMS data of compound 3i.

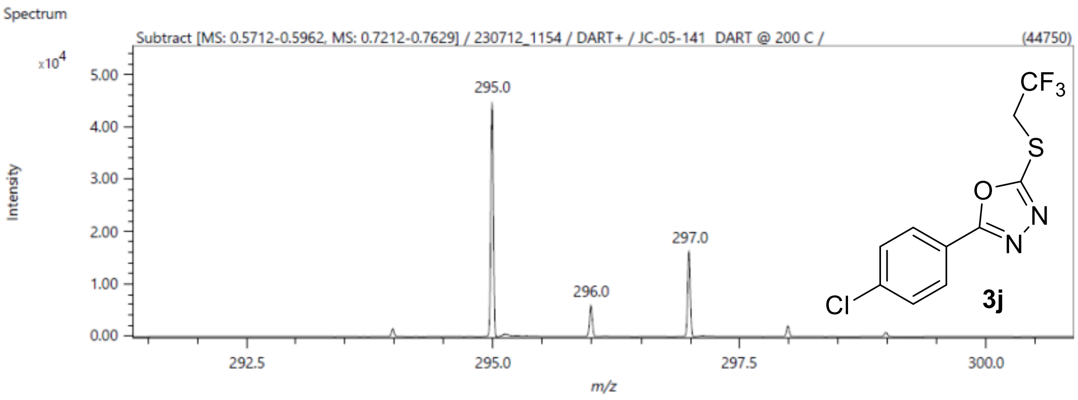

Elemental Composition

Parameters	
Tolerance:	±10.00 mDa
Electron:	Even
Charge:	+1
DBE:	-1.5 - 100.0

Elements Set 1:

Symbol	C	H	O	N	S	Cl	F
Min	0	0	0	0	1	1	3
Max	100	200	20	10	1	1	3

Results

Mass	Intensity	Formula	Calculated Mass	Mass Difference [mDa]	Mass Difference [ppm]	DBE
294.99184	44749.67	C10 H7 N2 O F3 S Cl	294.99142	0.42	1.42	6.5
		C5 H7 N4 O3 F3 S Cl	294.98740	4.44	15.06	2.5
		C4 H7 N6 O2 F3 S Cl	294.99863	-6.79	-23.02	2.5
		C H3 N10 O F3 S Cl	294.98471	7.13	24.16	3.5
		H7 N6 O5 F3 S Cl	294.98338	8.46	28.70	-1.5
		C8 H11 O4 F3 S Cl	295.00132	-9.48	-32.12	1.5

Supplementary Figure 15. HRMS data of compound 3j.

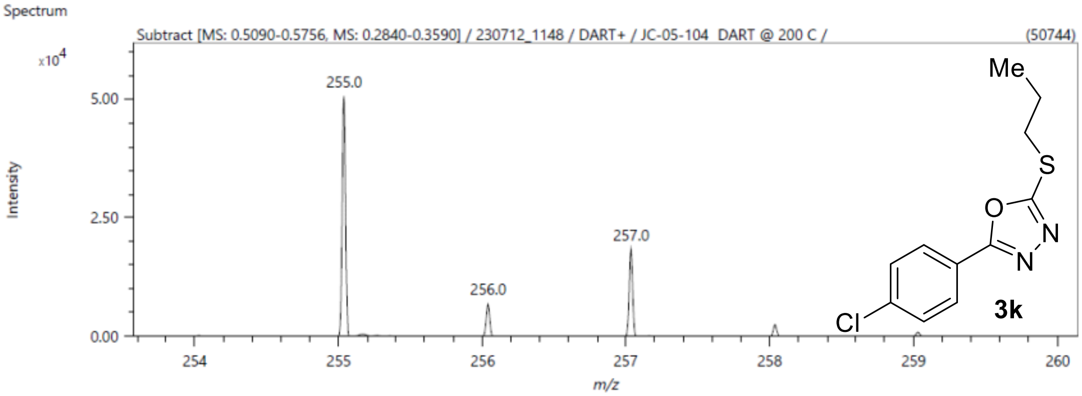

Elemental Composition

Parameters		Elements Set 1:						
Tolerance:	±10.00 mDa	Symbol	C	H	O	N	S	Cl
Electron:	Even	Min	0	0	0	0	1	1
Charge:	+1	Max	100	200	20	10	1	1
DBE:	-1.5 - 100.0							

Results

Mass	Intensity	Formula	Calculated Mass	Mass Difference [mDa]	Mass Difference [ppm]	DBE
255.03686	50743.80	C11 H12 N2 O S Cl	255.03534	1.52	5.97	6.5
		H12 N8 O4 S Cl	255.03853	-1.67	-6.53	-1.5
		C6 H12 N4 O3 S Cl	255.03132	5.55	21.74	2.5
		C5 H12 N6 O2 S Cl	255.04255	-5.69	-22.30	2.5
		C2 H8 N10 O S Cl	255.02863	8.23	32.27	3.5
		C9 H16 O4 S Cl	255.04523	-8.37	-32.83	1.5

Supplementary Figure 16. HRMS data of compound 3k.

Spectrum

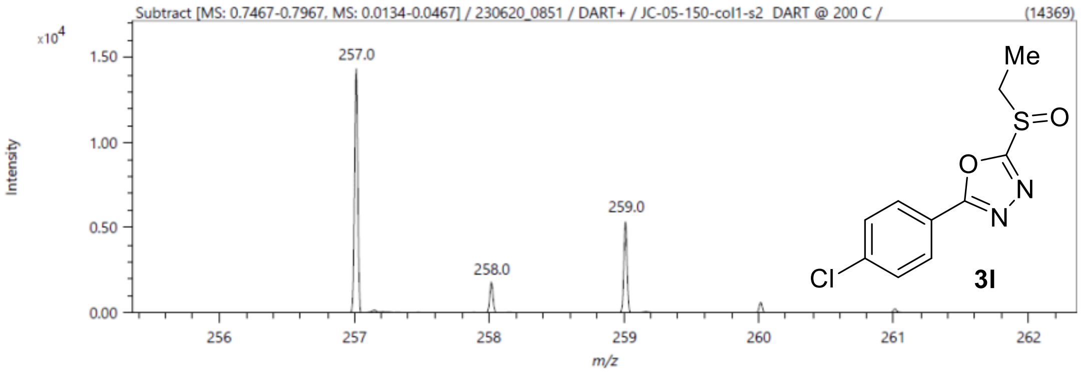

Elemental Composition

Parameters	
Tolerance:	±10.00 mDa
Electron:	Even
Charge:	+1
DBE:	-1.5 - 100.0

Elements Set 1:

Symbol	C	H	O	N	Cl	S
Min	0	0	0	0	0	1
Max	100	200	20	10	1	1

Results

Mass	Intensity	Formula	Calculated Mass	Mass Difference [mDa]	Mass Difference [ppm]	DBE
257.01394	14369.20	C10 H10 N2 O2 S Cl	257.01460	-0.66	-2.56	6.5
		C11 H5 N4 O2 S	257.01277	1.17	4.56	11.5
		H5 N10 O5 S	257.01596	-2.02	-7.84	3.5
		C6 H6 N8 S Cl	257.01192	2.03	7.89	7.5
		C10 H9 O6 S	257.01144	2.51	9.76	6.5
		C16 H5 N2 S	257.01680	-2.85	-11.09	15.5

Supplementary Figure 17. HRMS data of compound 3l.

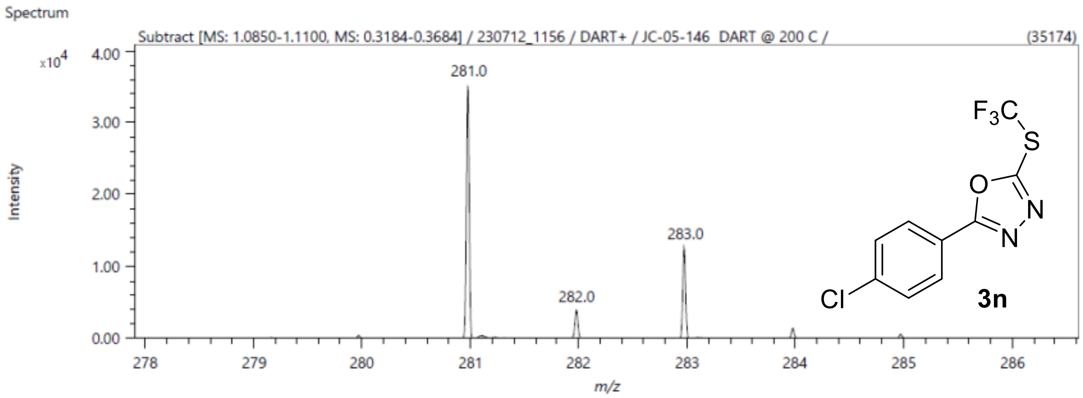

Elemental Composition

Parameters		Elements Set 1:							
Tolerance:	±10.00 mDa	Symbol	C	H	O	N	S	Cl	F
Electron:	Even	Min	0	0	0	0	1	1	3
Charge:	+1	Max	100	200	20	10	1	1	3
DBE:	-1.5 - 100.0								

Results

Mass	Intensity	Formula	Calculated Mass	Mass Difference [mDa]	Mass Difference [ppm]	DBE
280.97676	35173.71	C9 H5 N2 O F3 S Cl	280.97577	0.99	3.53	6.5
		C4 H5 N4 O3 F3 S Cl	280.97175	5.01	17.84	2.5
		C3 H5 N6 O2 F3 S Cl	280.98298	-6.22	-22.14	2.5
		H N10 O F3 S Cl	280.96906	7.70	27.40	3.5
		C7 H9 O4 F3 S Cl	280.98567	-8.90	-31.69	1.5

Supplementary Figure 18. HRMS data of compound 3n.

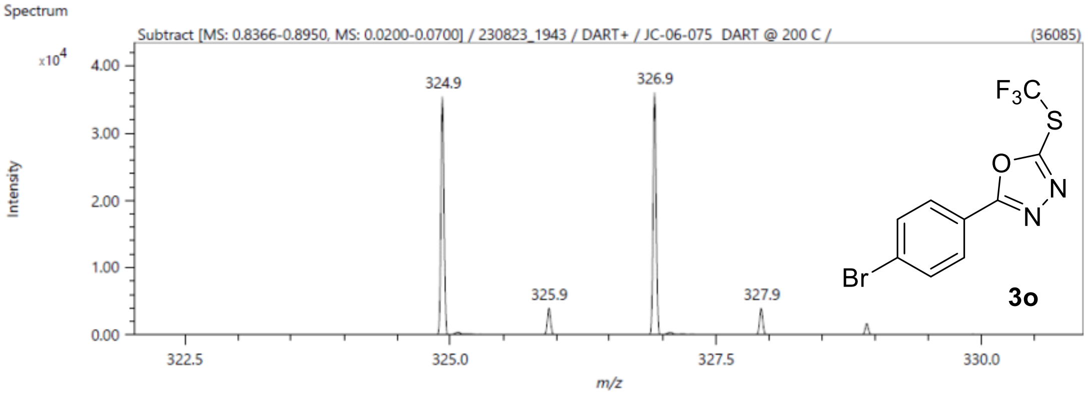

Elemental Composition

Parameters		Elements Set 1:							
Tolerance:	±10.00 mDa	Symbol	C	H	O	N	S	F	Br
Electron:	Even	Min	0	0	0	0	0	3	1
Charge:	+1	Max	100	200	10	10	1	3	1
DBE:	-1.5 - 100.0								

Results

Mass	Intensity	Formula	Calculated Mass	Mass Difference [mDa]	Mass Difference [ppm]	DBE
324.92590	35486.22	C9 H5 N2 O F3 S Br	324.92526	0.64	1.97	6.5
		C H N8 O4 F3 Br	324.92507	0.82	2.53	3.5
		C5 H5 N2 O6 F3 Br	324.92776	-1.86	-5.73	2.5
		H5 N4 O8 F3 Br	324.92374	2.16	6.65	-1.5
		C6 H N6 O2 F3 Br	324.92910	-3.20	-9.85	7.5
		C12 H N2 O F3 Br	324.92189	4.01	12.34	11.5

Supplementary Figure 19. HRMS data of compound 3o.

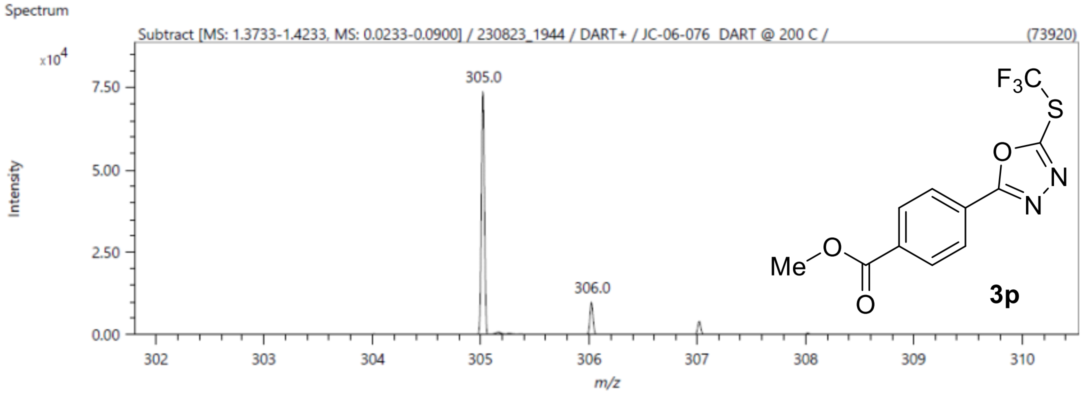

Elemental Composition

Parameters		Elements Set 1:						
Tolerance:	±10.00 mDa	Symbol	C	H	O	N	S	F
Electron:	Even	Min	0	0	0	0	0	3
Charge:	+1	Max	100	200	10	10	1	3
DBE:	-1.5 - 100.0							

Results

Mass	Intensity	Formula	Calculated Mass	Mass Difference [mDa]	Mass Difference [ppm]	DBE
305.01979	73919.98	C3 H4 N8 O6 F3	305.02004	-0.25	-0.83	4.5
		C11 H8 N2 O3 F3 S	305.02022	-0.44	-1.43	7.5
		C2 H8 N4 O10 F3	305.01870	1.09	3.56	-0.5
		C19 H4 O F3	305.02088	-1.09	-3.56	16.5
		C7 H4 N8 O F3 S	305.01754	2.25	7.38	8.5
		C14 H4 N2 O3 F3	305.01685	2.94	9.62	12.5

Supplementary Figure 20. HRMS data of compound 3p.

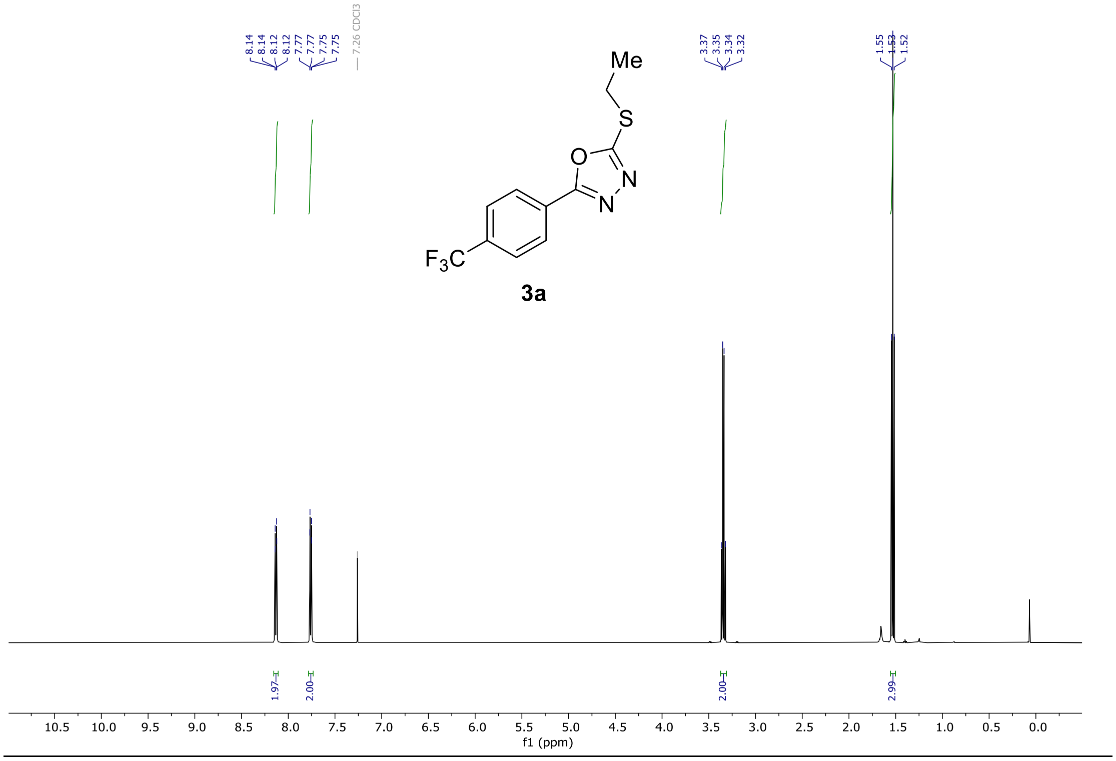

Supplementary Figure 21. ^{1}H NMR spectra of compound 3a. $CDCl_3$ solvent and 500 MHz frequency.

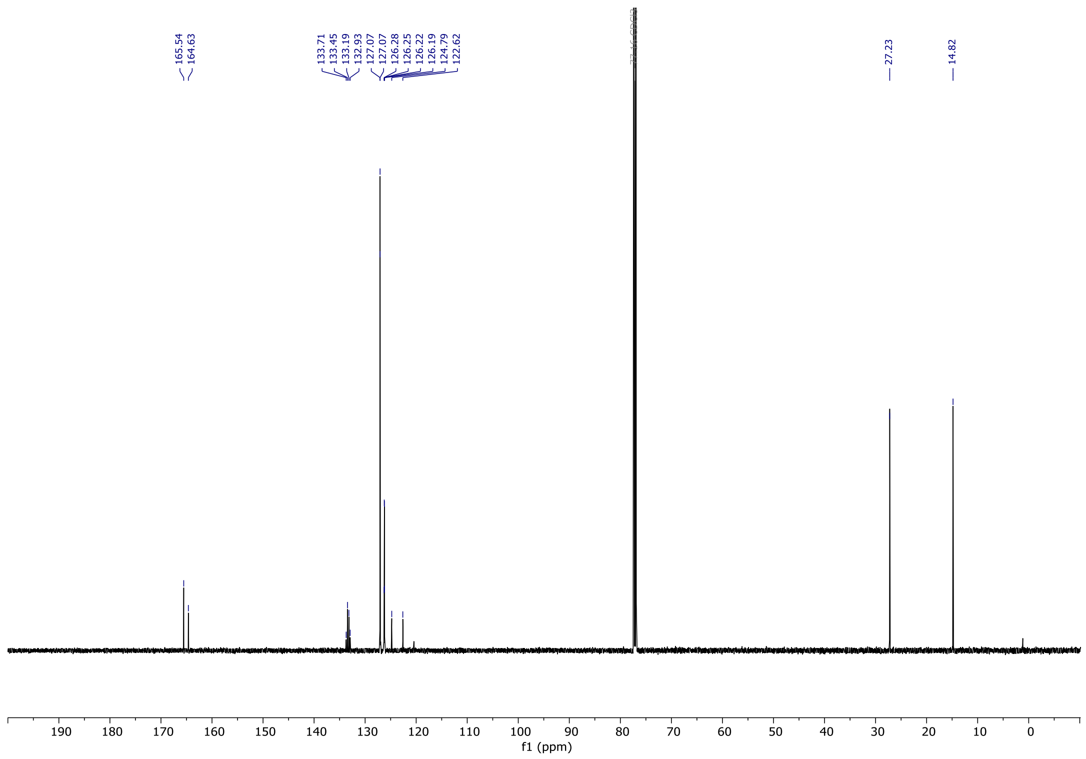

Supplementary Figure 22. ^{13}C NMR spectra of compound 3a. $CDCl_3$ solvent and 125 MHz frequency.

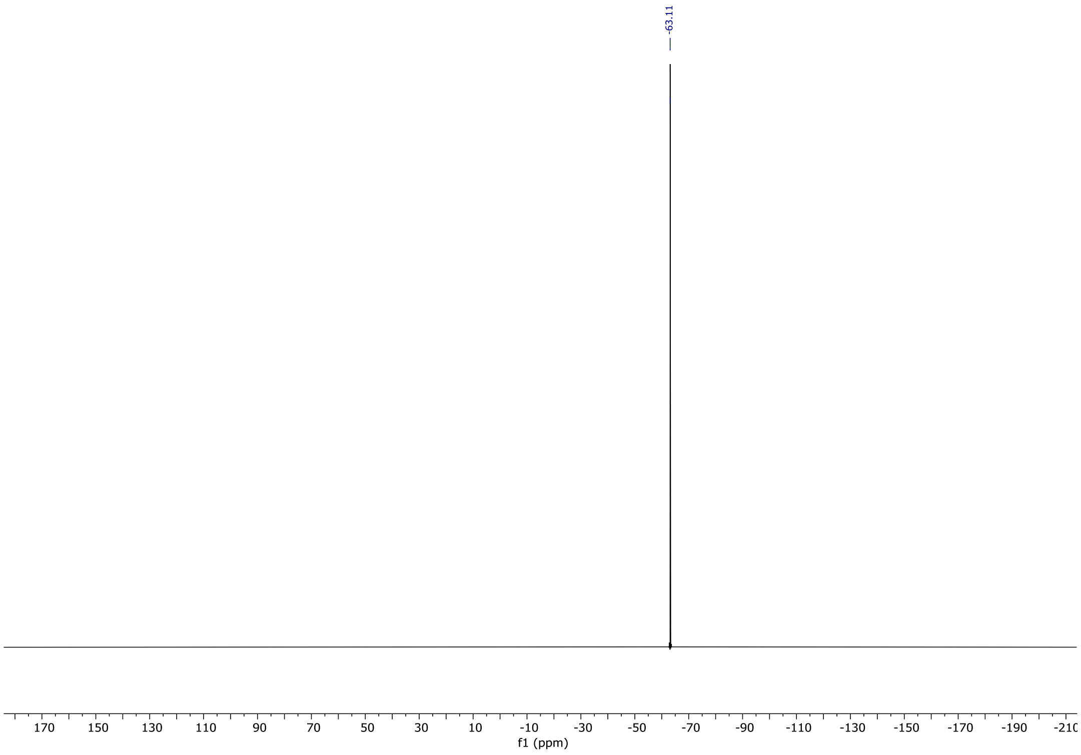

Supplementary Figure 23. ^{19}F NMR spectra of compound 3a. $CDCl_3$ solvent and 375 MHz frequency.

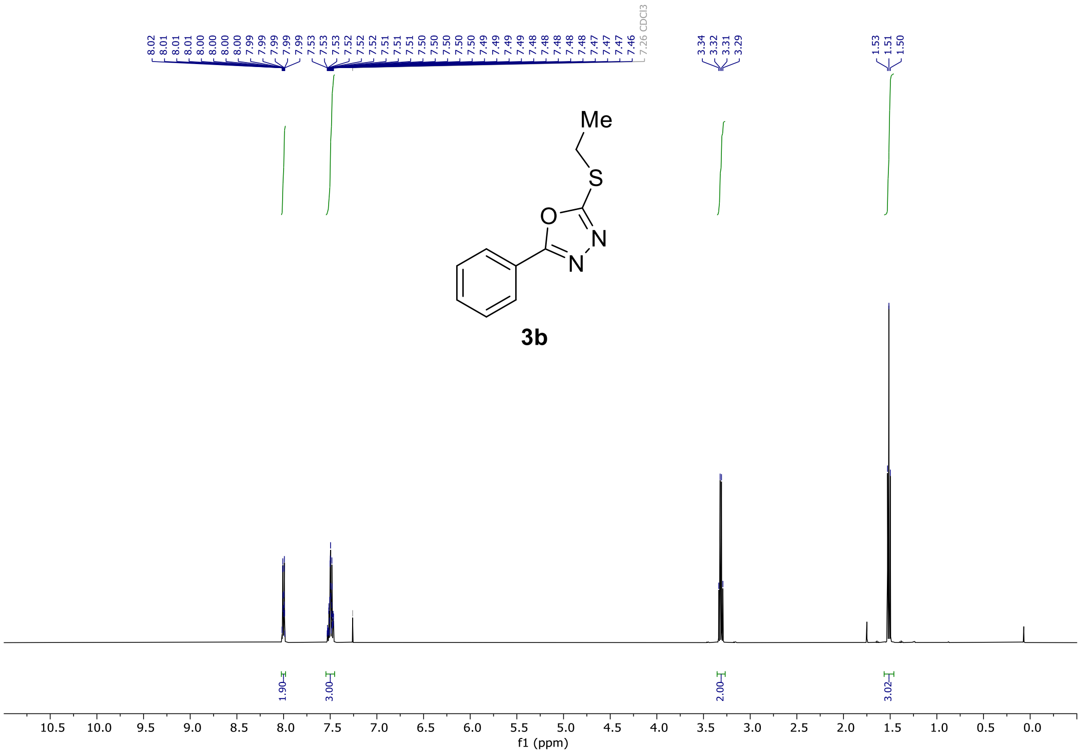

Supplementary Figure 24. ^{1}H NMR spectra of compound 3b. $CDCl_3$ solvent and 500 MHz frequency.

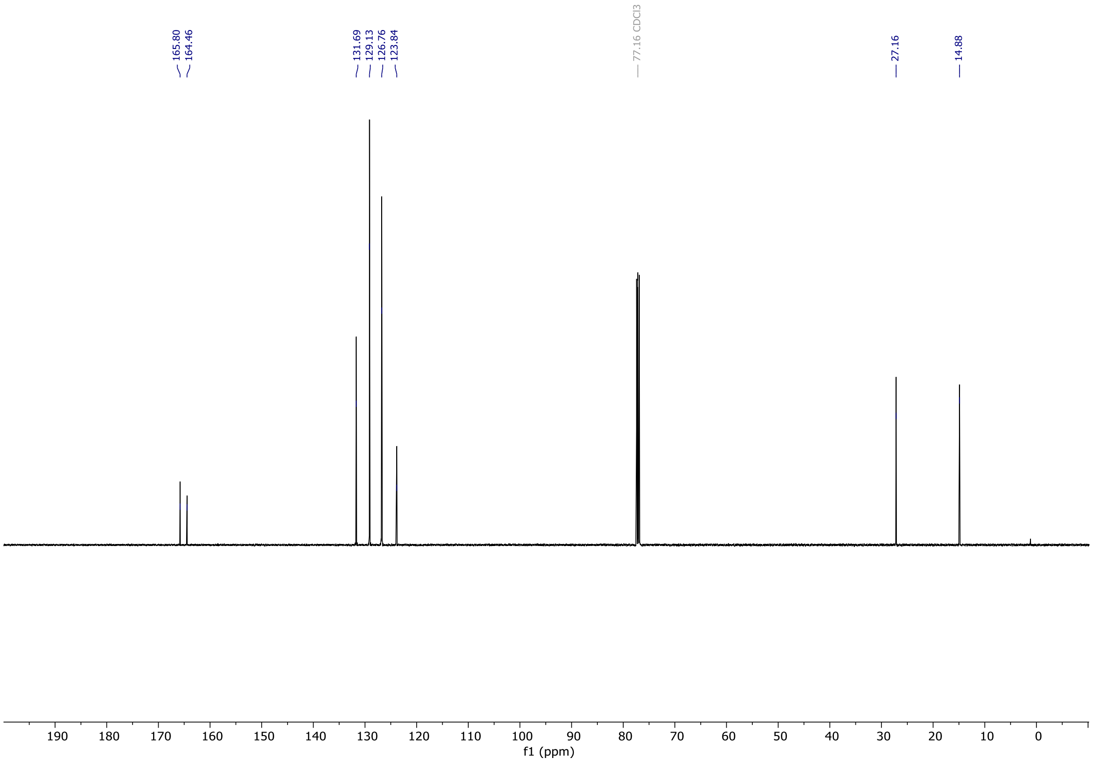

Supplementary Figure 25. ^{13}C NMR spectra of compound 3b. $CDCl_3$ solvent and 125 MHz frequency.

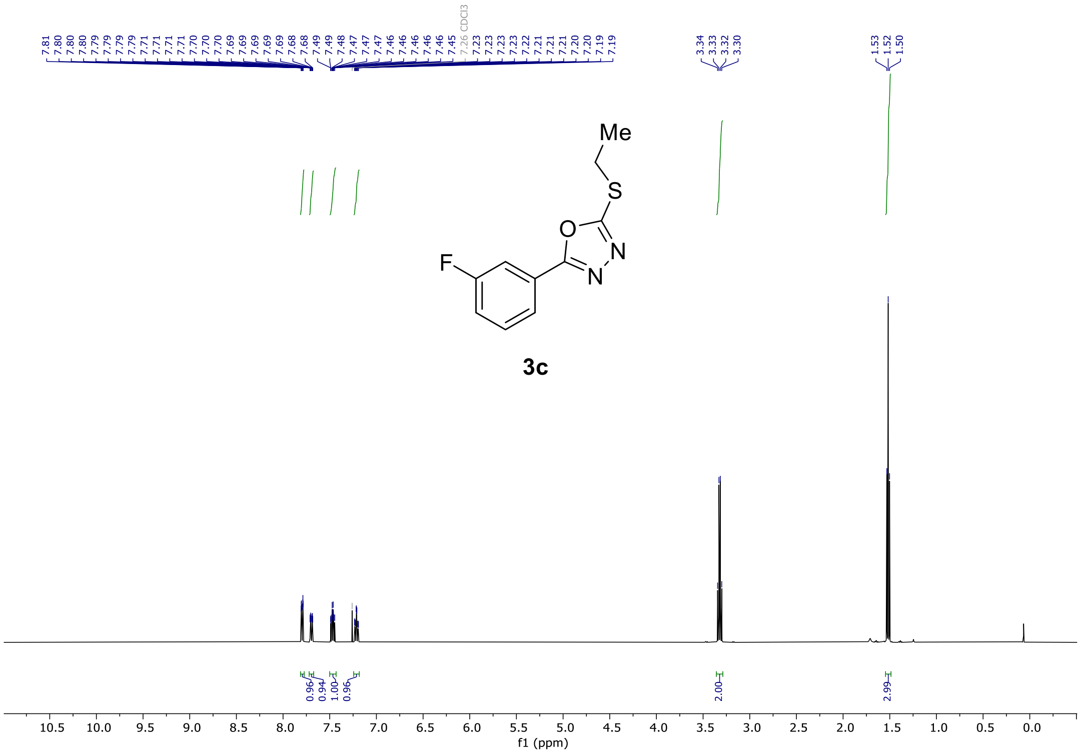

Supplementary Figure 26. ^{1}H NMR spectra of compound 3c. $CDCl_3$ solvent and 500 MHz frequency.

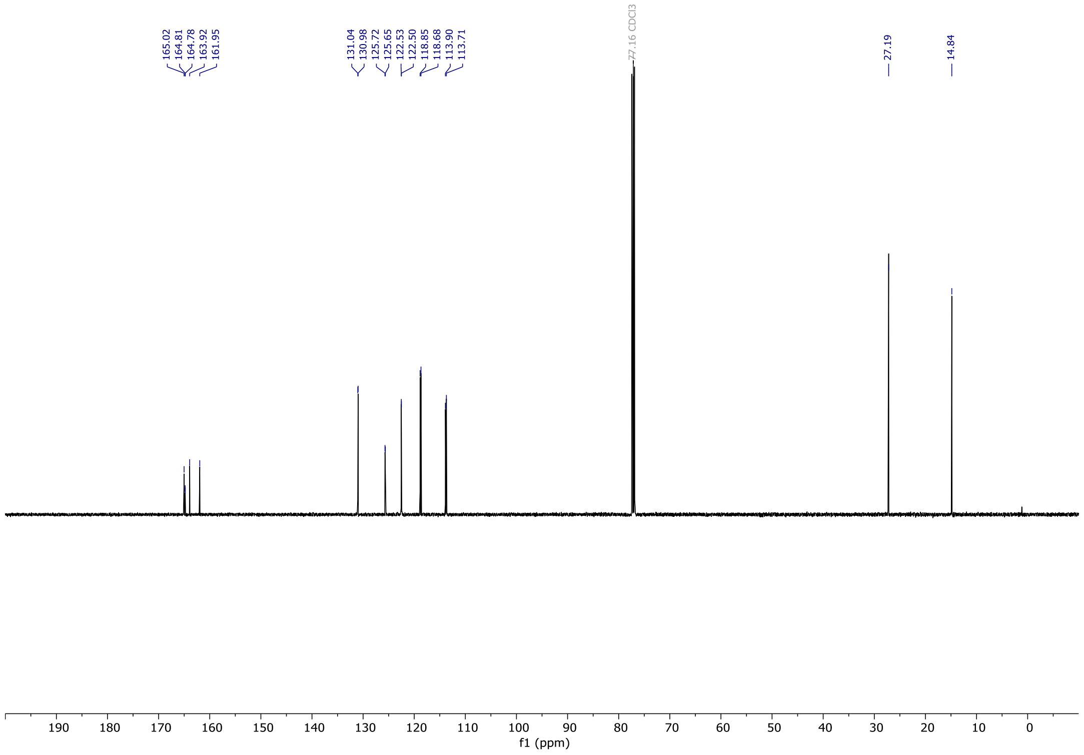

Supplementary Figure 27. ^{13}C NMR spectra of compound 3c. $CDCl_3$ solvent and 125 MHz frequency.

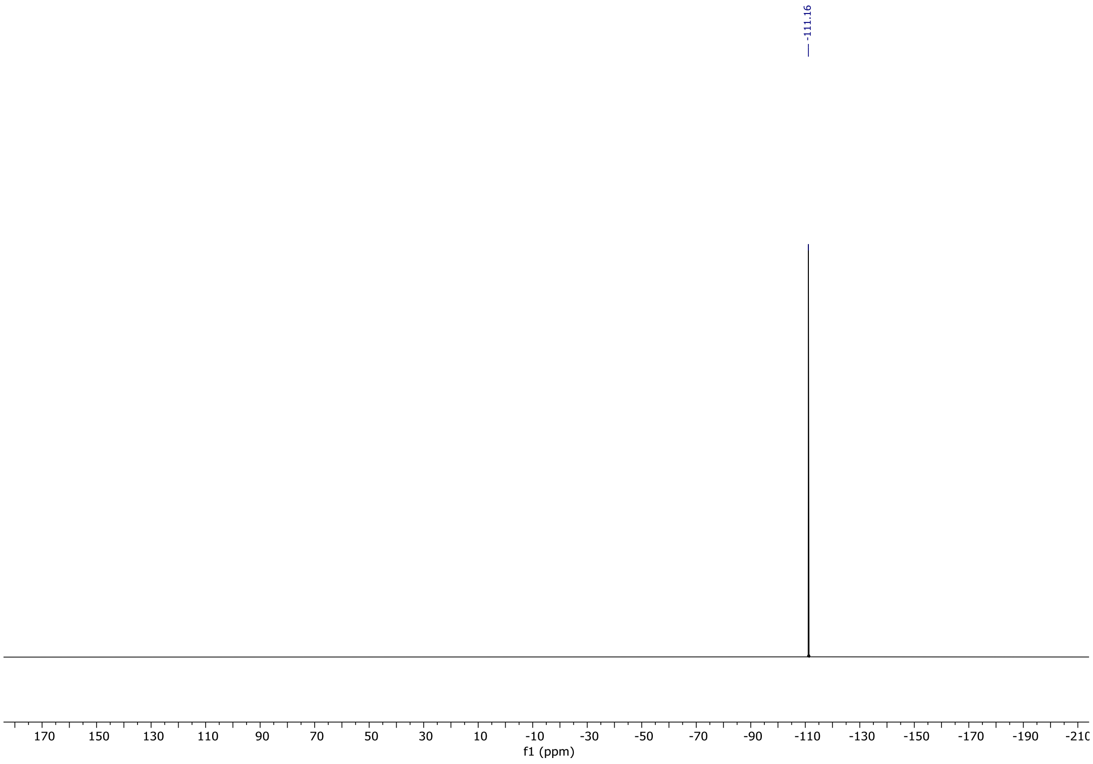

Supplementary Figure 28. ^{19}F NMR spectra of compound 3c. $CDCl_3$ solvent and 375 MHz frequency.

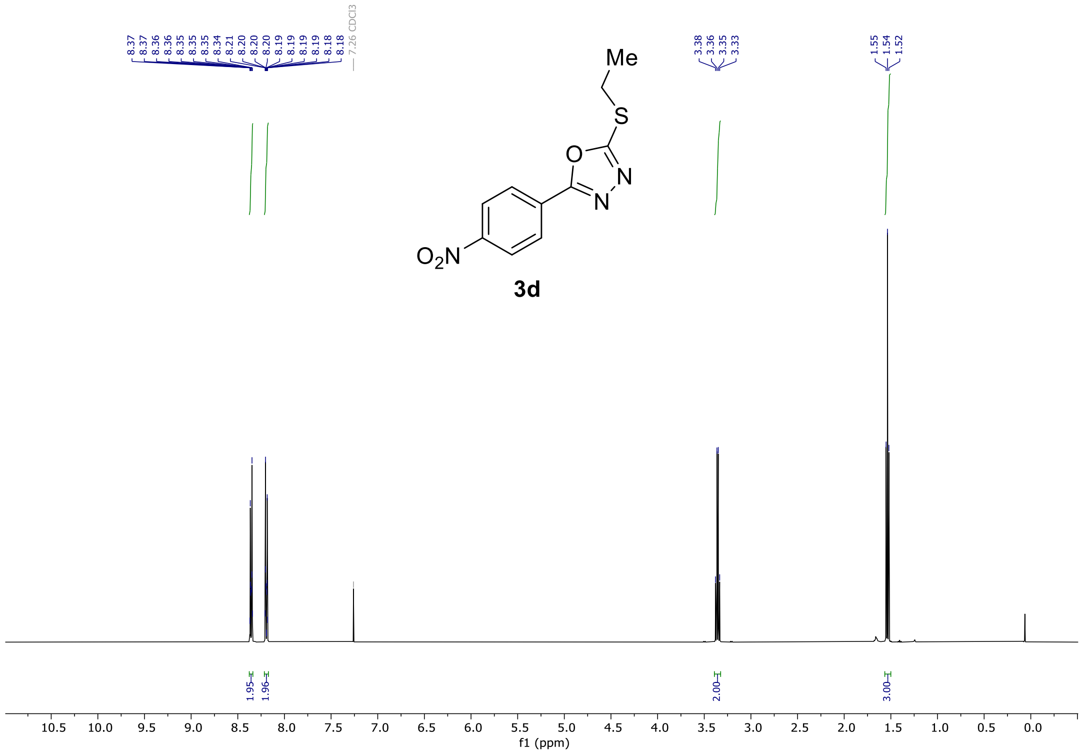

Supplementary Figure 29. ^{1}H NMR spectra of compound 3d. $CDCl_3$ solvent and 500 MHz frequency.

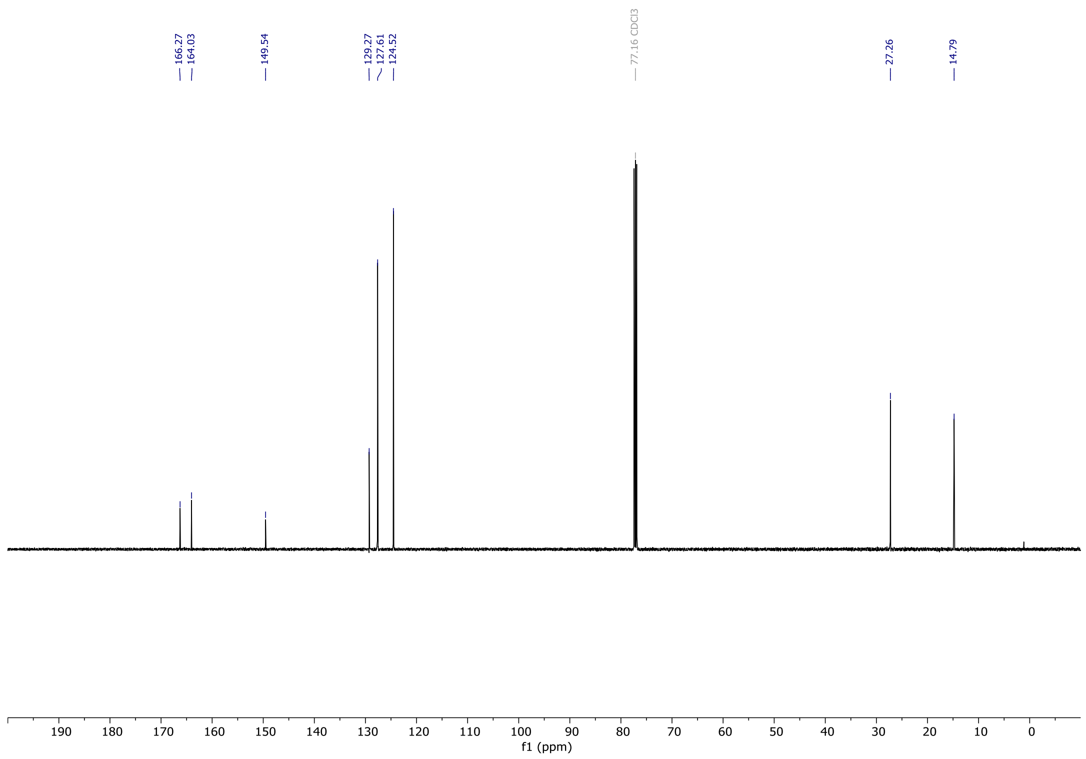

Supplementary Figure 30. ^{13}C NMR spectra of compound 3d. $CDCl_3$ solvent and 125 MHz frequency.

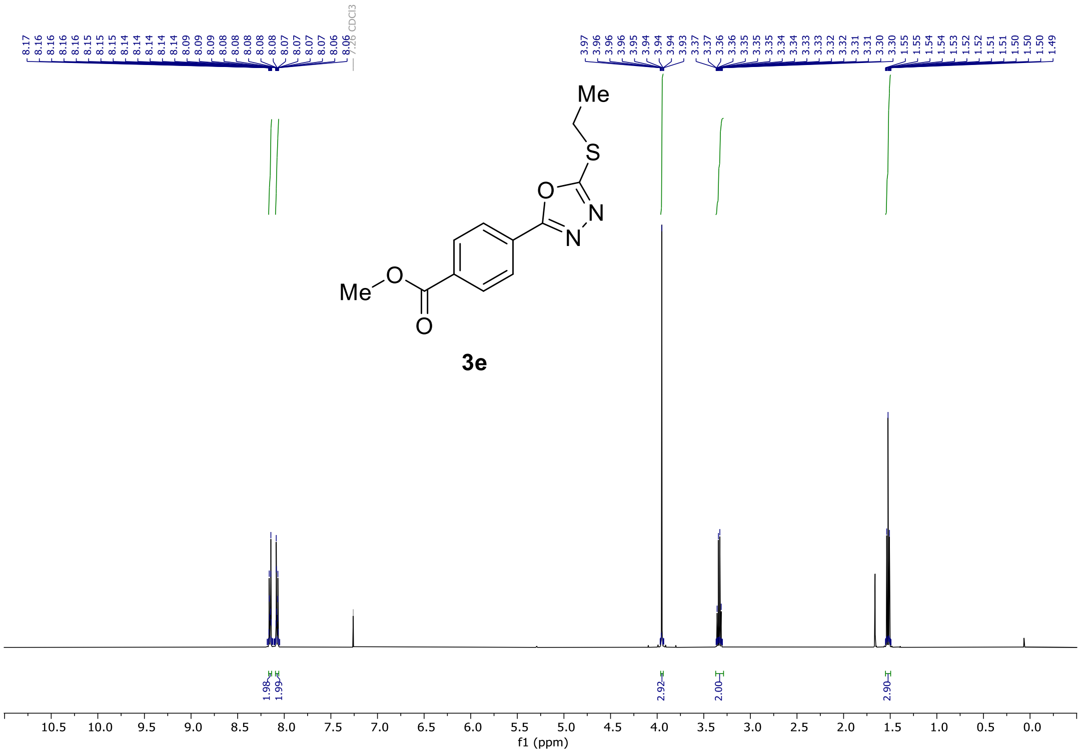

Supplementary Figure 31. ^{1}H NMR spectra of compound 3e. $CDCl_3$ solvent and 500 MHz frequency.

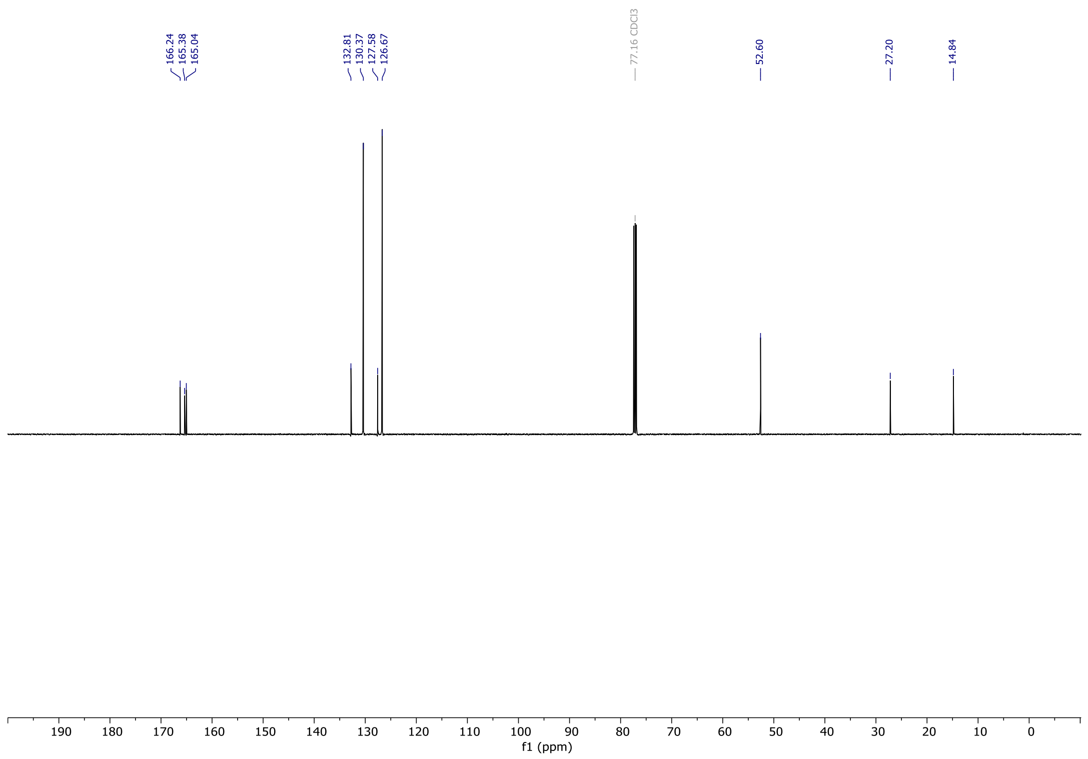

Supplementary Figure 32. ^{13}C NMR spectra of compound 3e. $CDCl_3$ solvent and 125 MHz frequency.

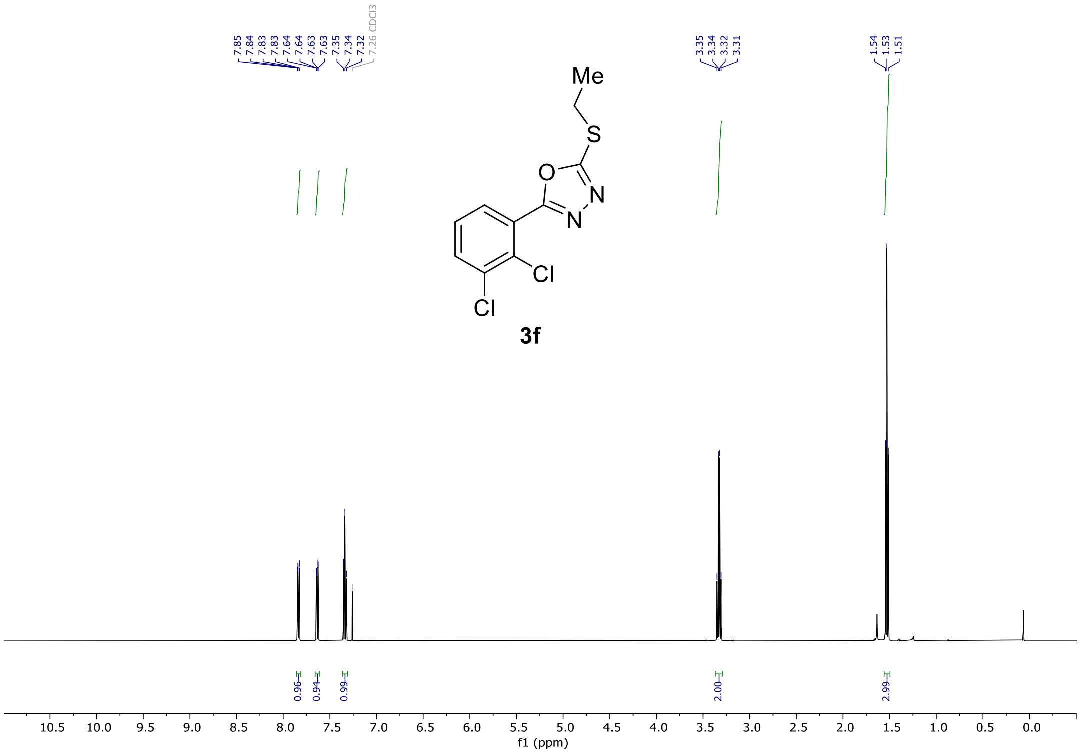

Supplementary Figure 33. ¹H NMR spectra of compound 3f. $CDCl_3$ solvent and 500 MHz frequency.

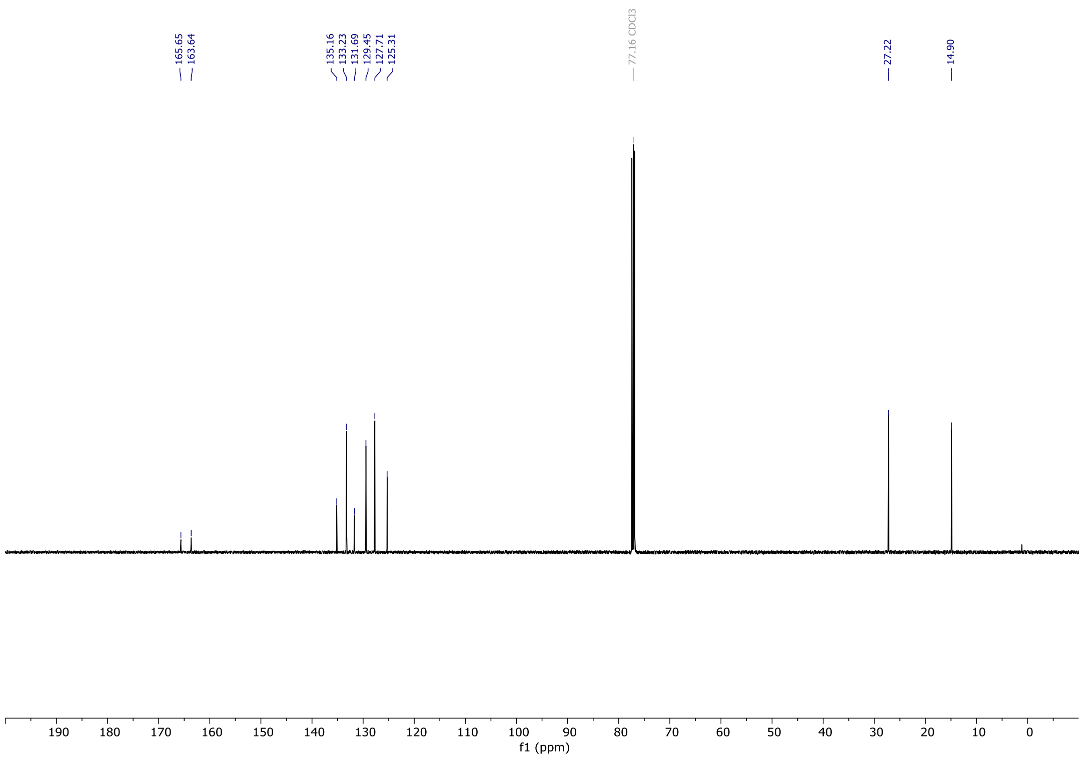

Supplementary Figure 34. ^{13}C NMR spectra of compound 3f. $CDCl_3$ solvent and 125 MHz frequency.

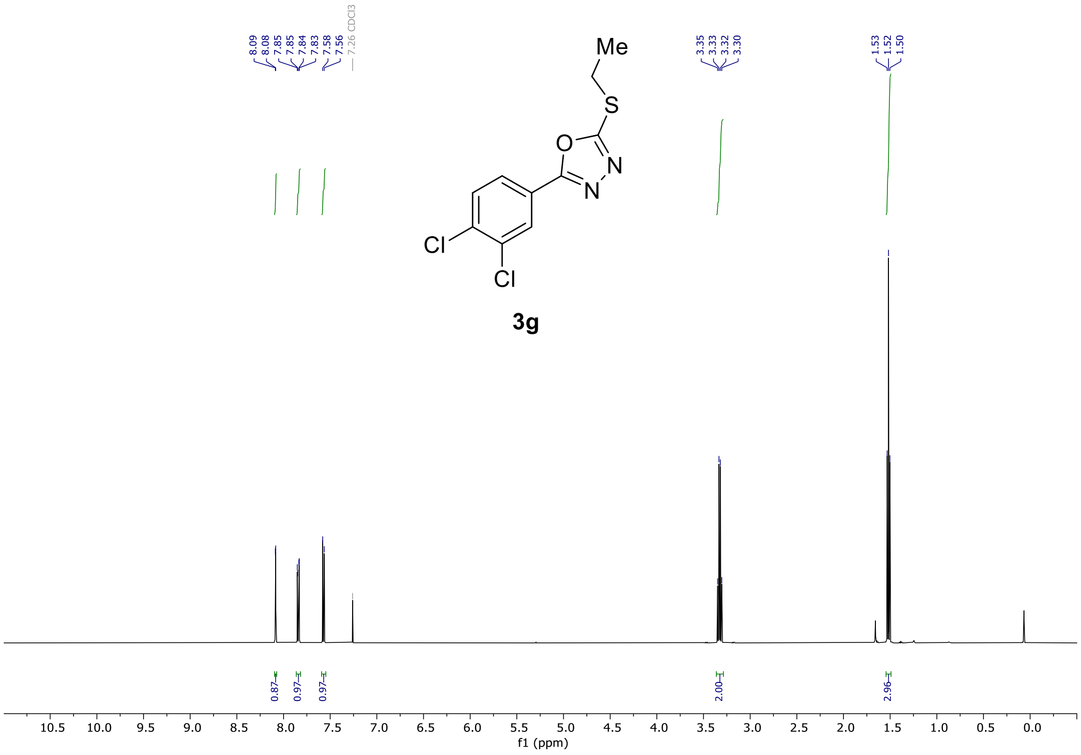

Supplementary Figure 35. ^{1}H NMR spectra of compound 3g. $CDCl_3$ solvent and 500 MHz frequency.

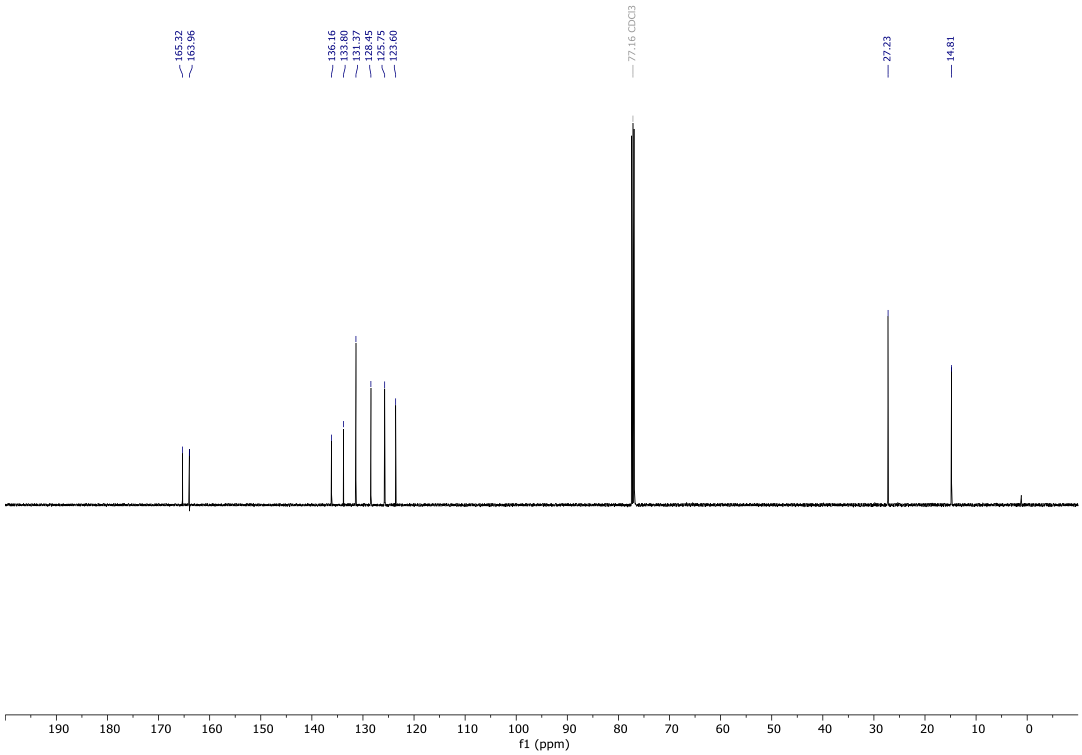

Supplementary Figure 36. ^{13}C NMR spectra of compound 3g. $CDCl_3$ solvent and 125 MHz frequency.

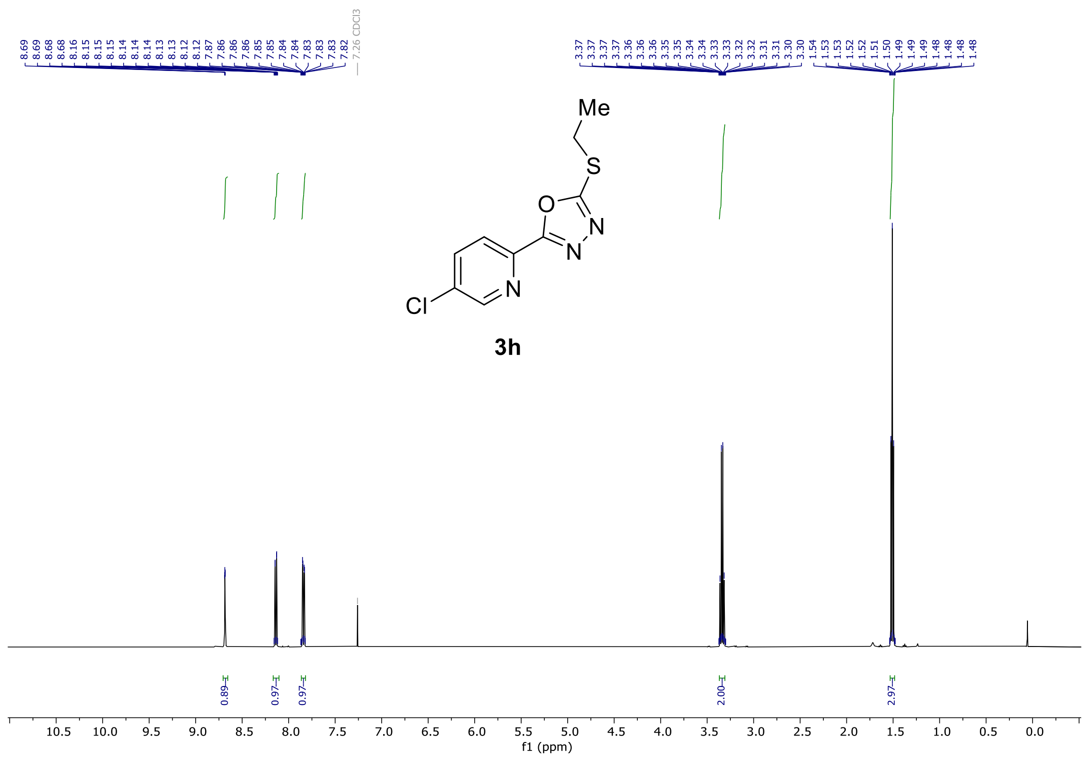

Supplementary Figure 37. ¹H NMR spectra of compound 3h. $CDCl_3$ solvent and 500 MHz frequency.

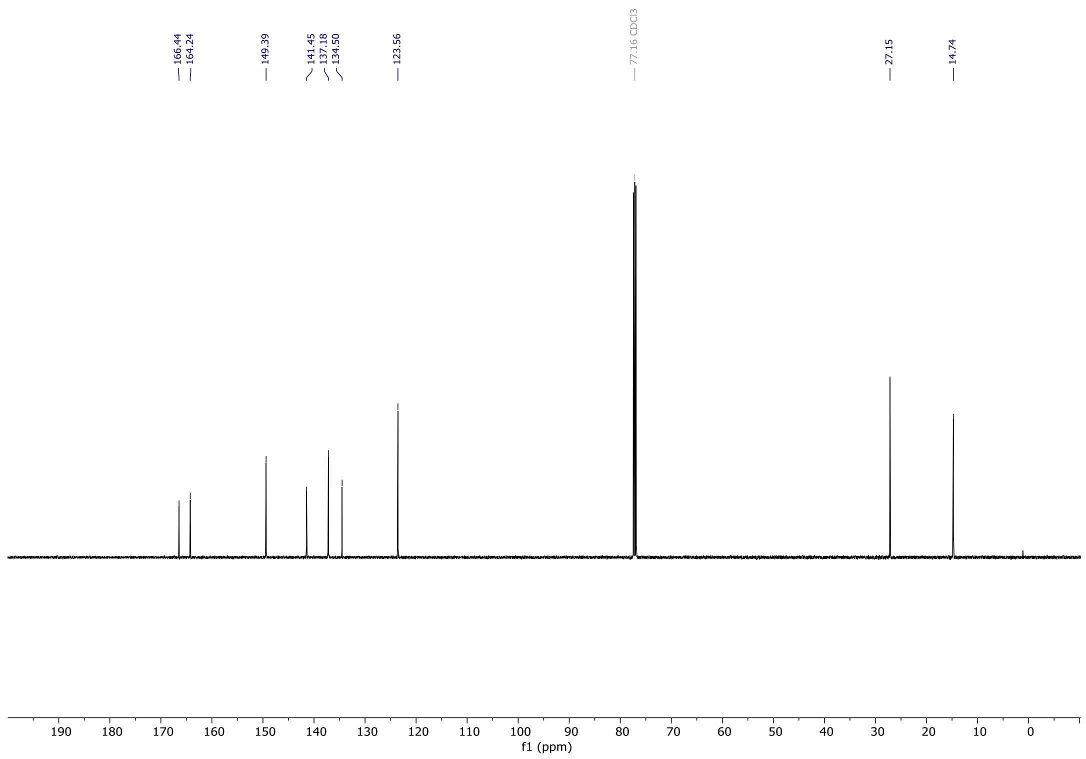

Supplementary Figure 38. ^{13}C NMR spectra of compound 3h. $CDCl_3$ solvent and 125 MHz frequency.

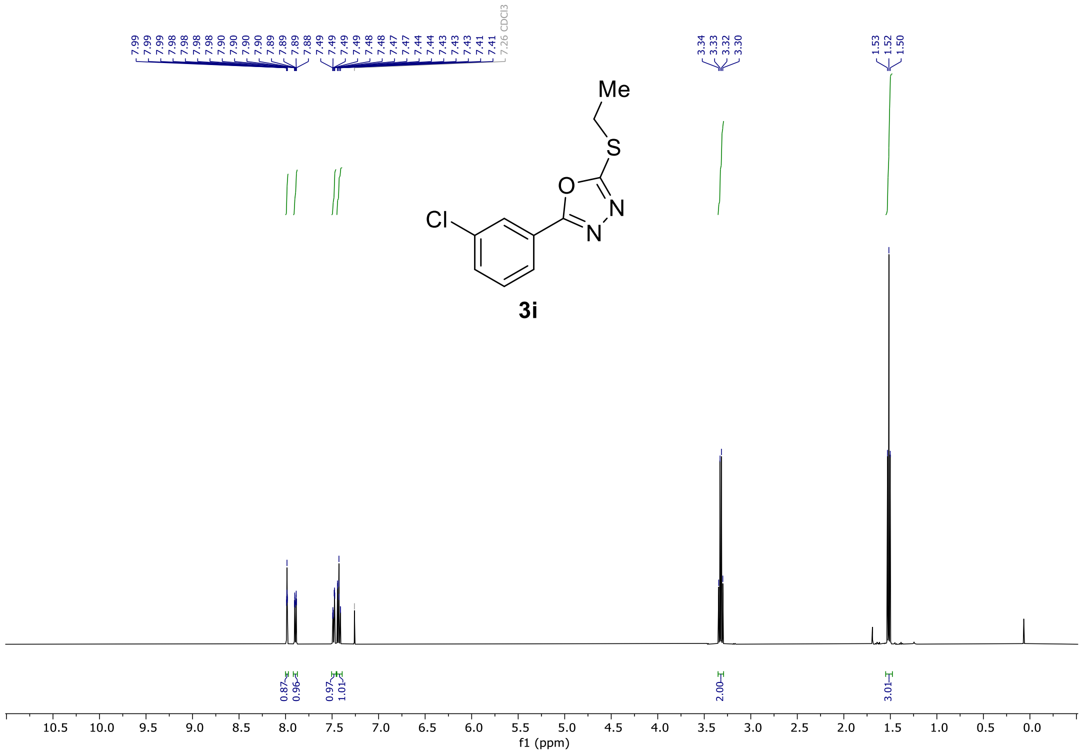

Supplementary Figure 39. ¹H NMR spectra of compound 3i. $CDCl_3$ solvent and 500 MHz frequency.

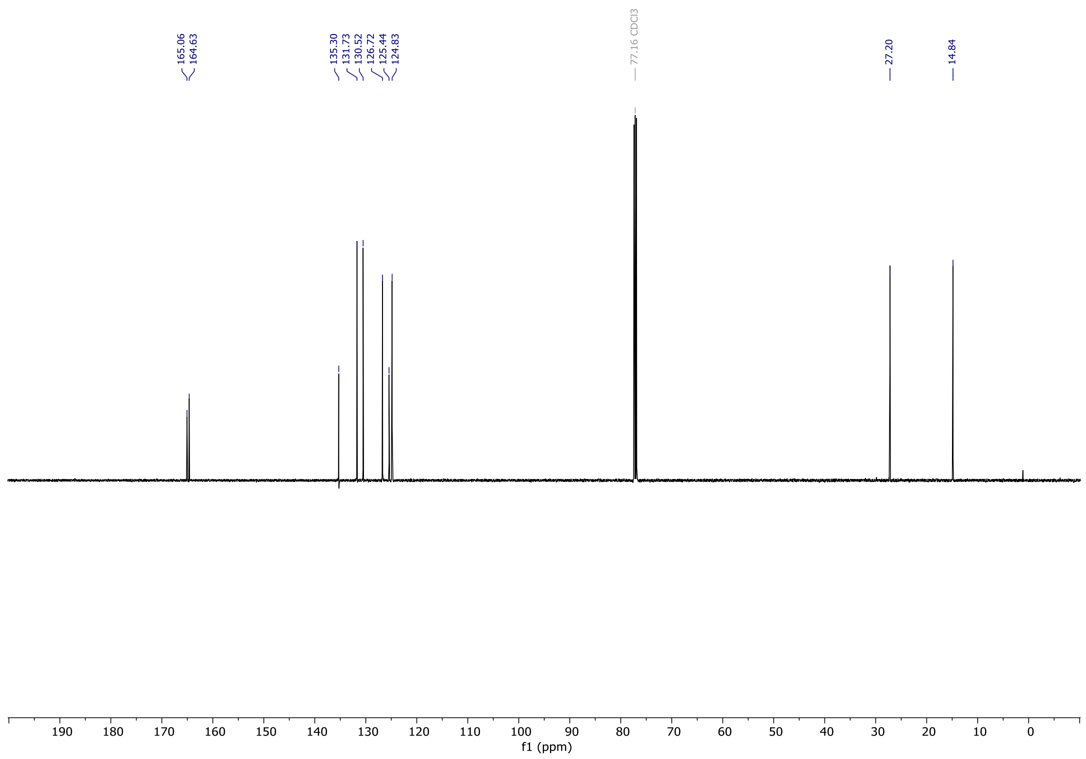

Supplementary Figure 40. ^{13}C NMR spectra of compound 3i. $CDCl_3$ solvent and 125 MHz frequency.

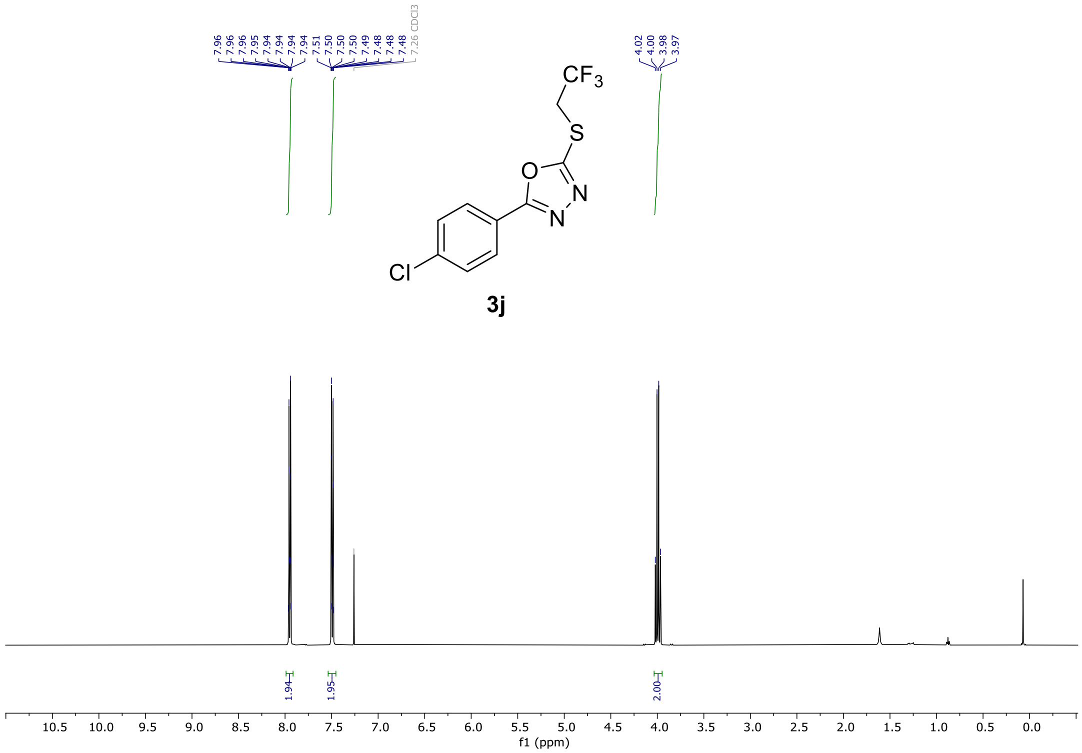

Supplementary Figure 41. ¹H NMR spectra of compound 3j. $CDCl_3$ solvent and 500 MHz frequency.

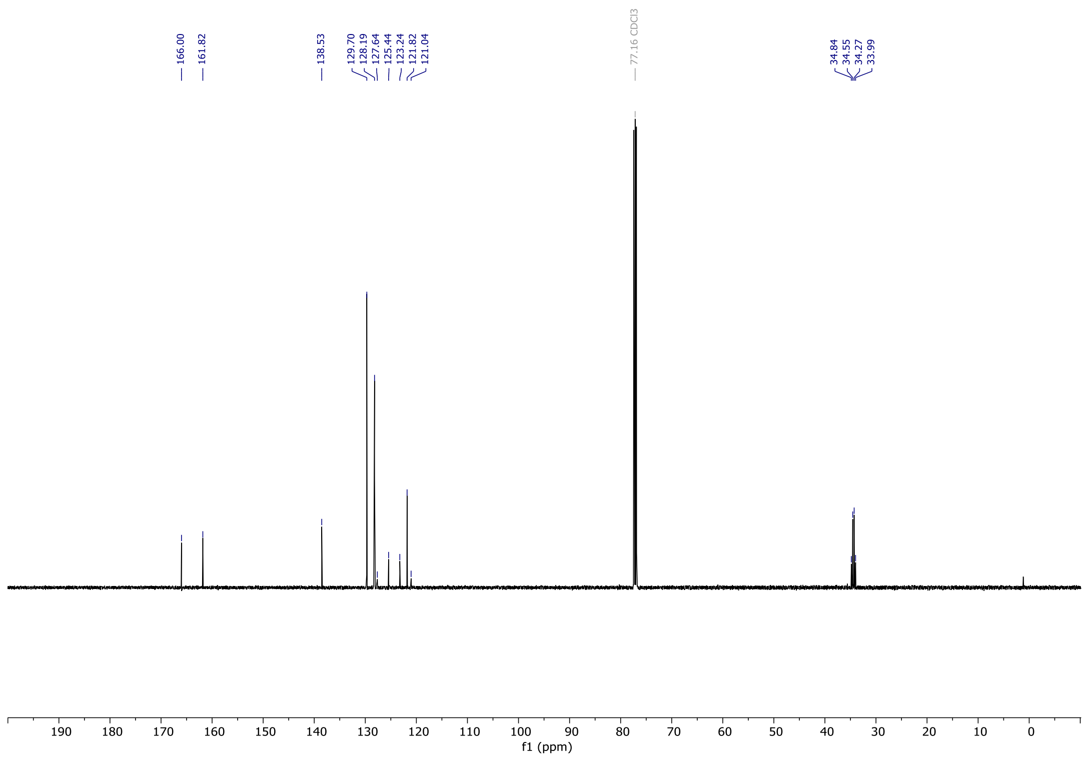

Supplementary Figure 42. ^{13}C NMR spectra of compound 3j. $CDCl_3$ solvent and 125 MHz frequency.

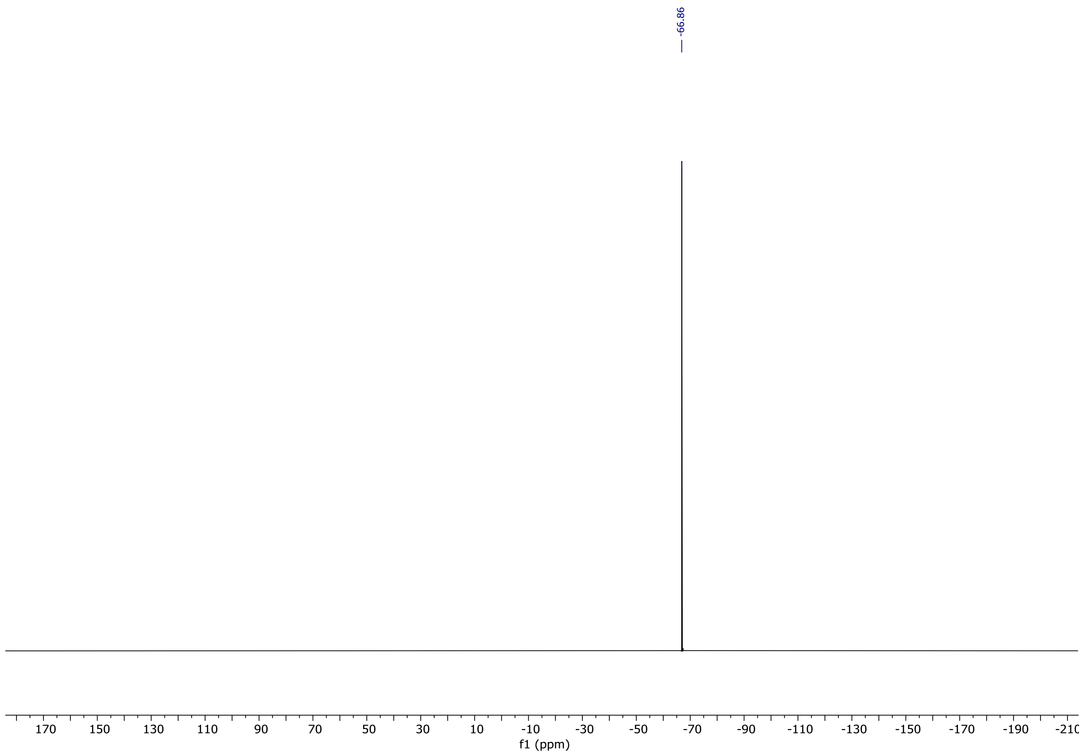

Supplementary Figure 43. ^{19}F NMR spectra of compound 3j. $CDCl_3$ solvent and 375 MHz frequency.

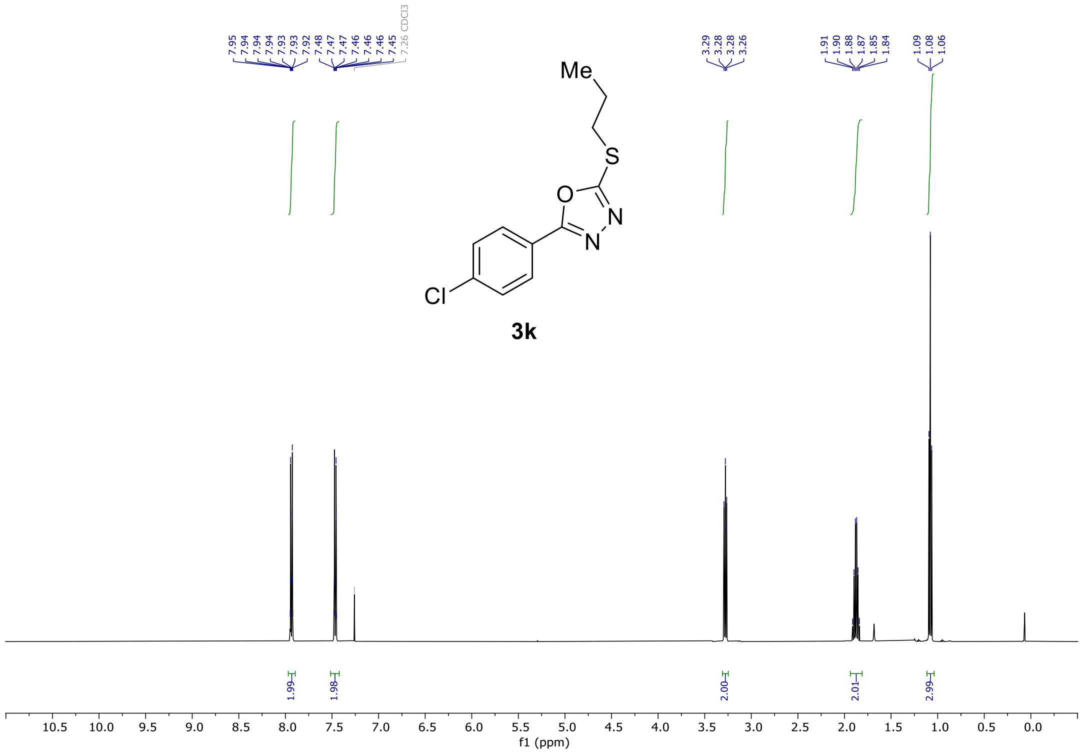

Supplementary Figure 44. ¹H NMR spectra of compound 3k. $CDCl_3$ solvent and 500 MHz frequency.

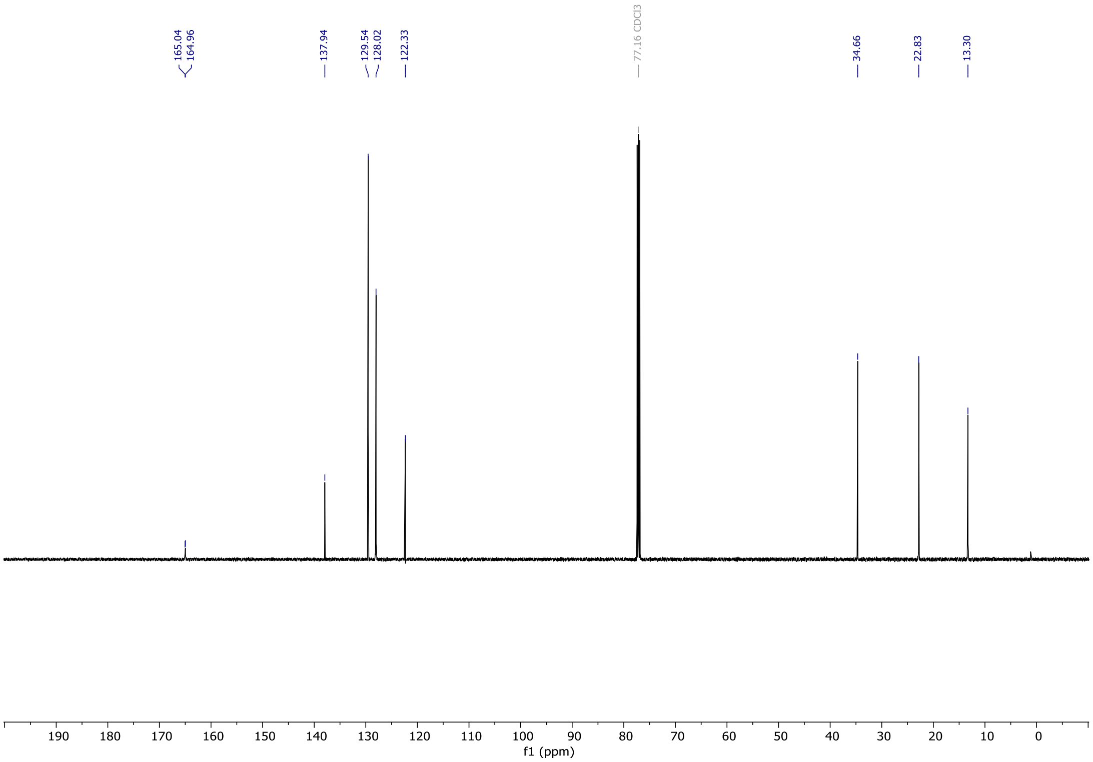

Supplementary Figure 45. ^{13}C NMR spectra of compound 3k. $CDCl_3$ solvent and 125 MHz frequency.

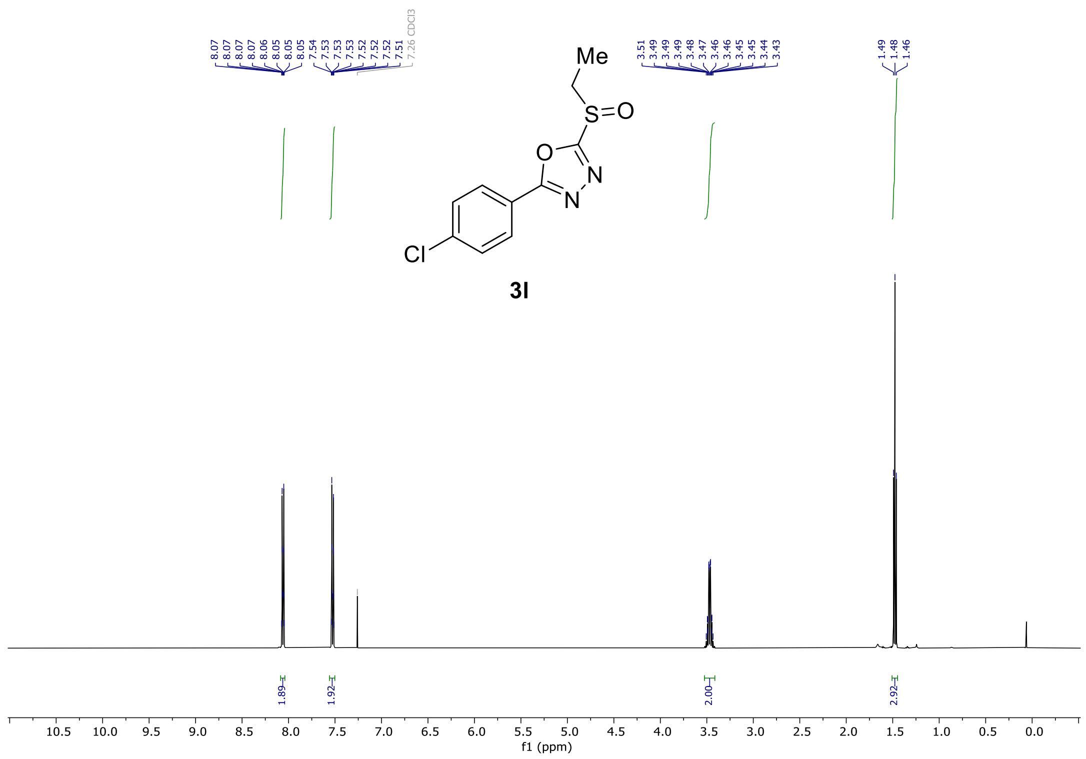

Supplementary Figure 46. ^{1}H NMR spectra of compound 3l. $CDCl_3$ solvent and 500 MHz frequency.

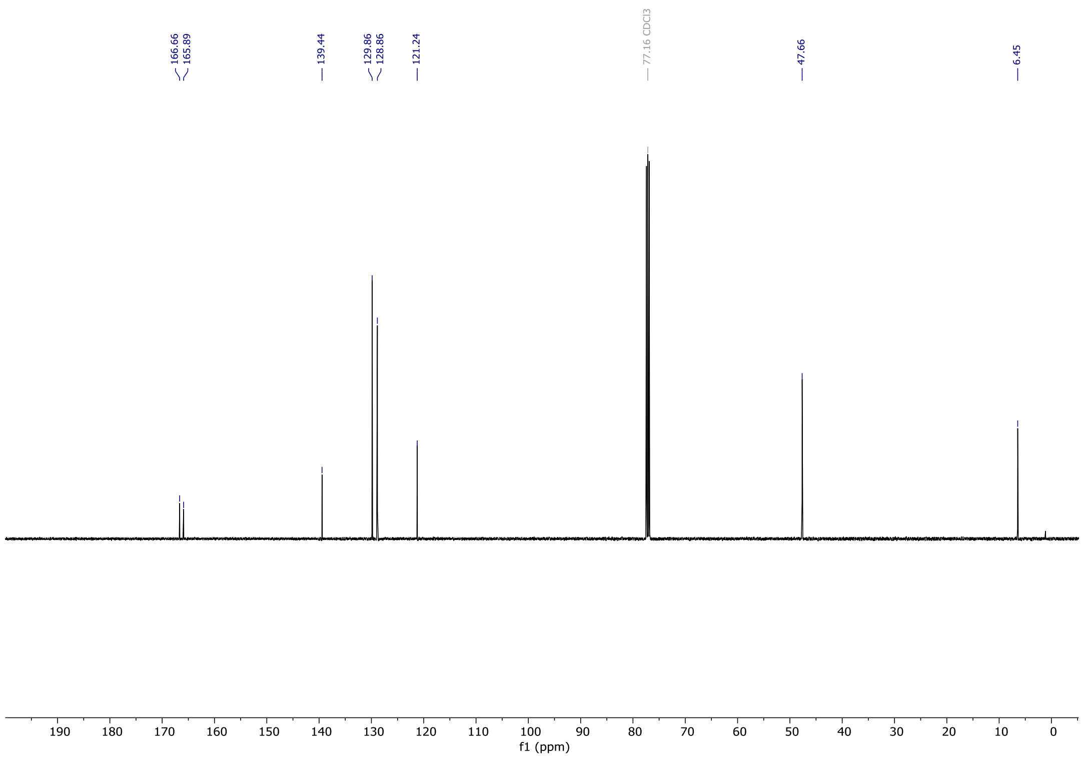

Supplementary Figure 47. ^{13}C NMR spectra of compound 3l. $CDCl_3$ solvent and 125 MHz frequency.

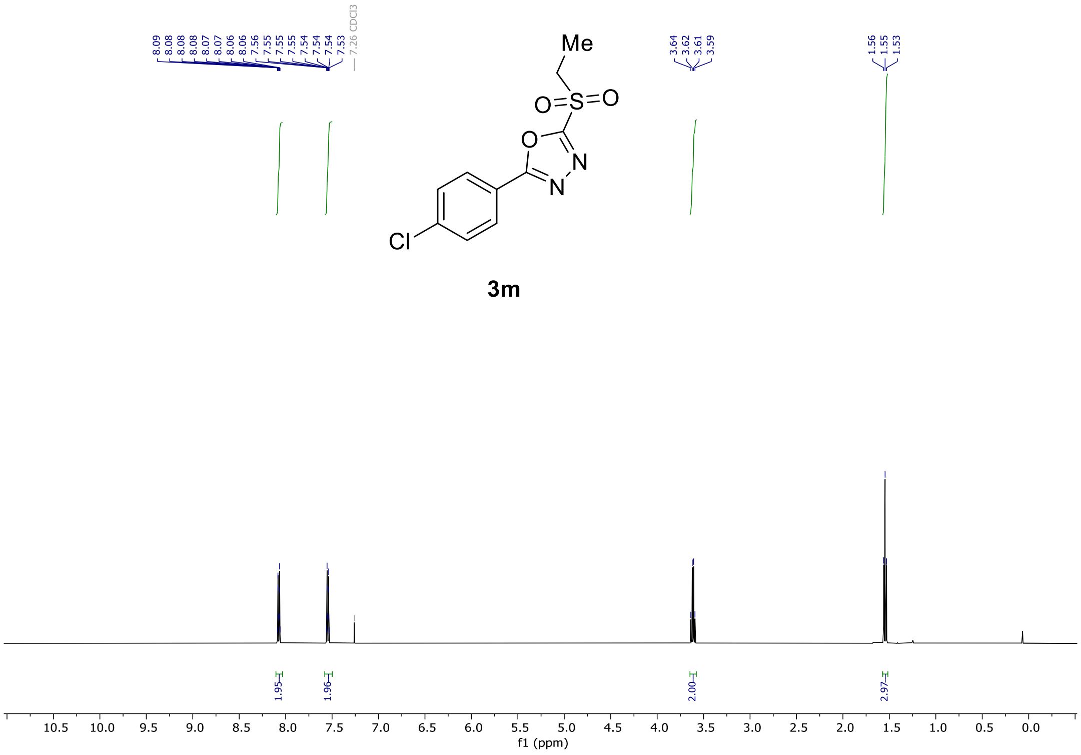

3m

Supplementary Figure 48. ¹H NMR spectra of compound 3m. $CDCl_3$ solvent and 500 MHz frequency.

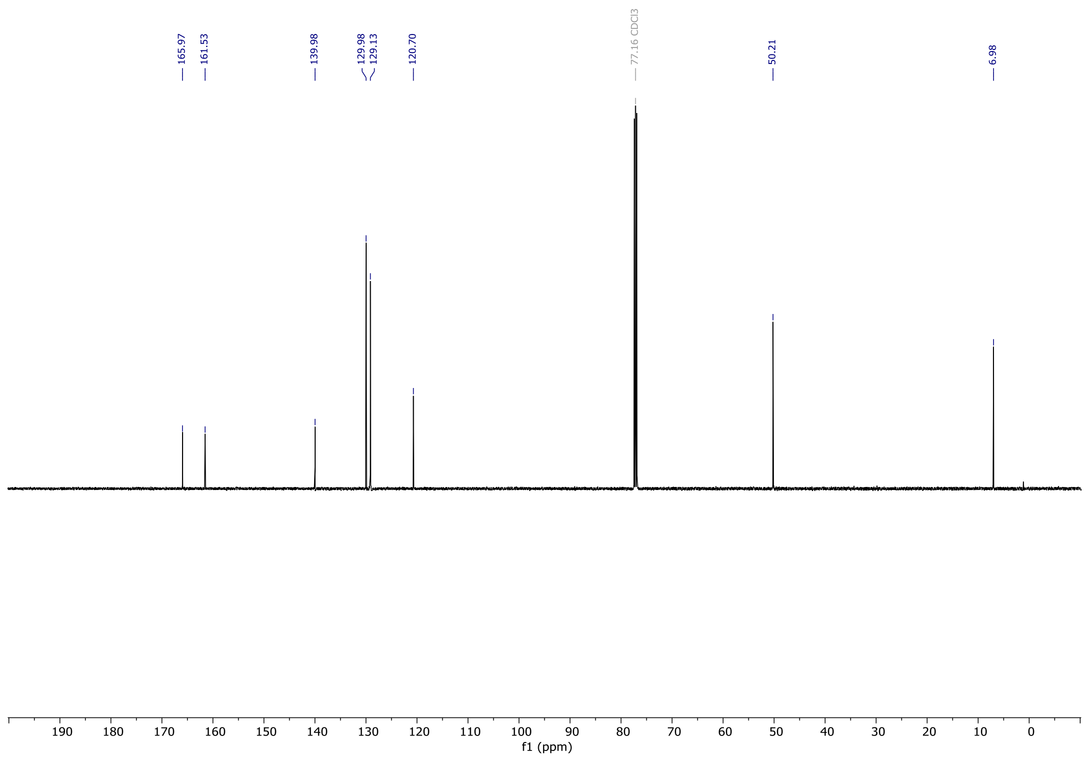

Supplementary Figure 49. ^{13}C NMR spectra of compound 3m. $CDCl_3$ solvent and 125 MHz frequency.

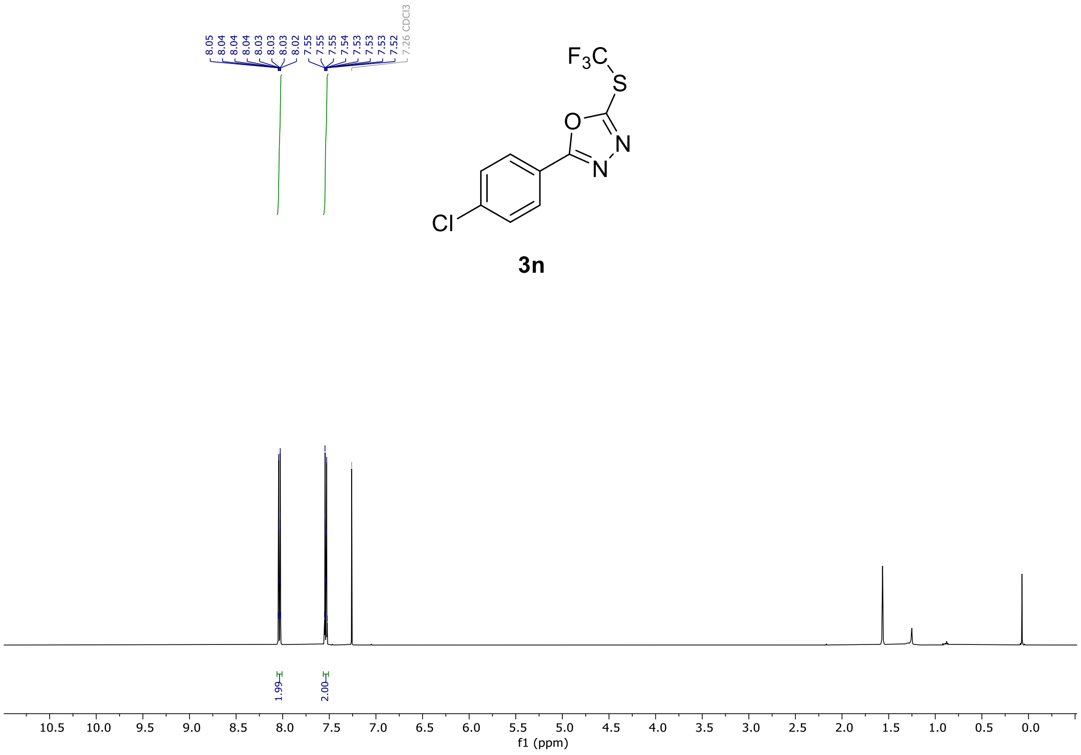

3n

Supplementary Figure 50. ^{1}H NMR spectra of compound 3n. $CDCl_3$ solvent and 500 MHz frequency.

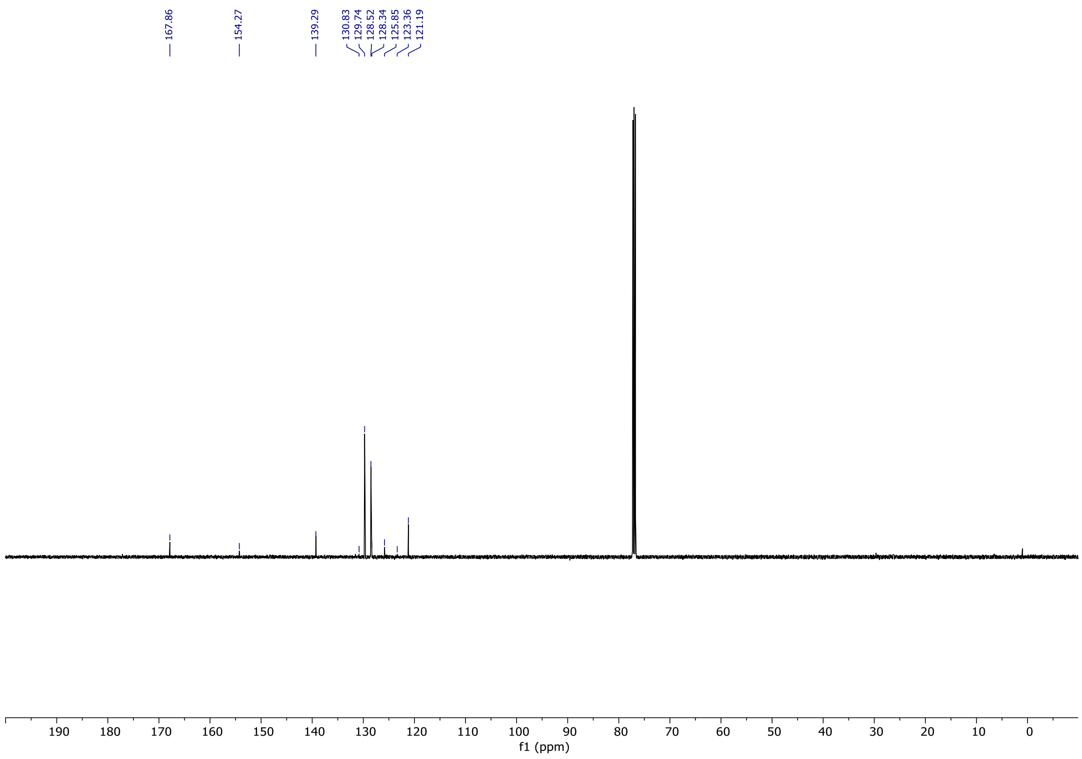

Supplementary Figure 51. ^{13}C NMR spectra of compound 3n. $CDCl_3$ solvent and 125 MHz frequency.

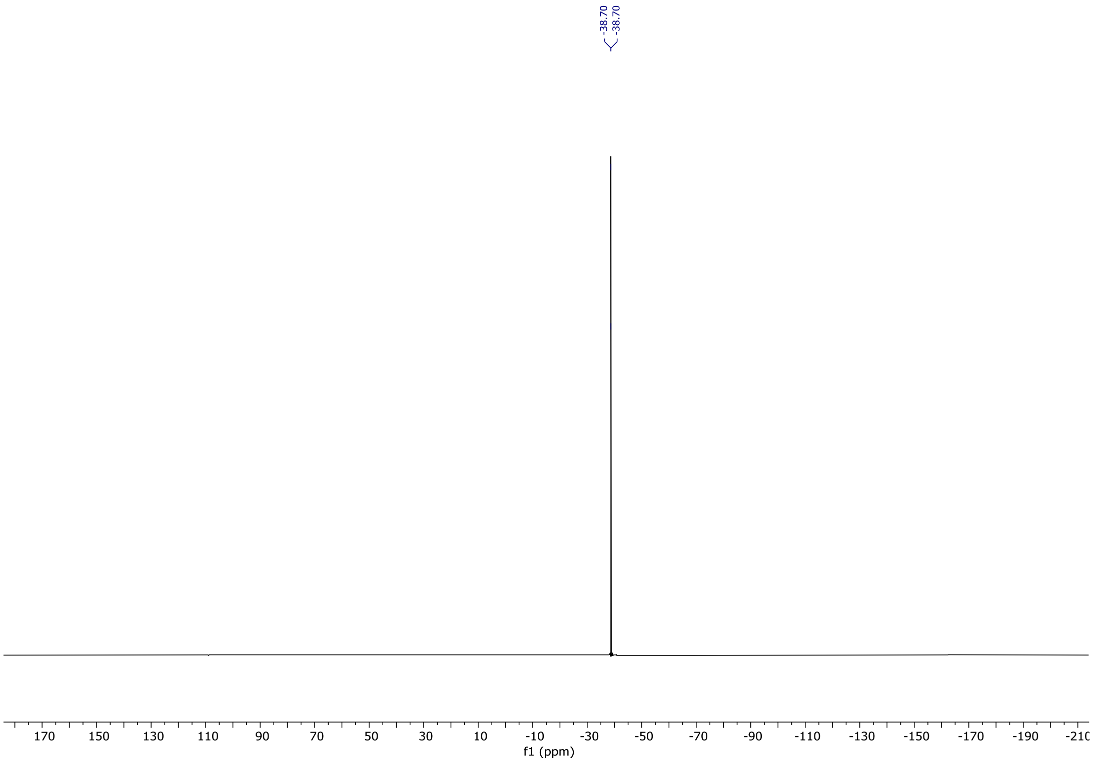

Supplementary Figure 52. ^{19}F NMR spectra of compound 3n. $CDCl_3$ solvent and 375 MHz frequency.

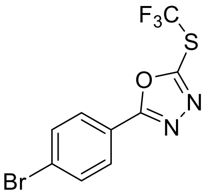

3o

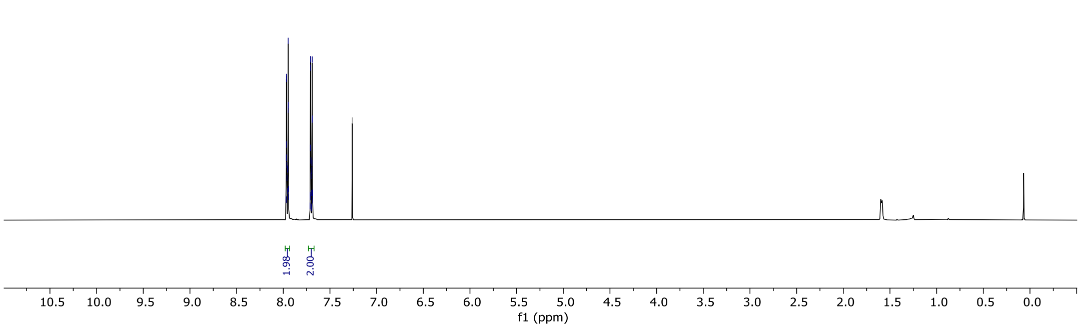

Supplementary Figure 53. ¹H NMR spectra of compound 3o. $CDCl_3$ solvent and 500 MHz frequency.

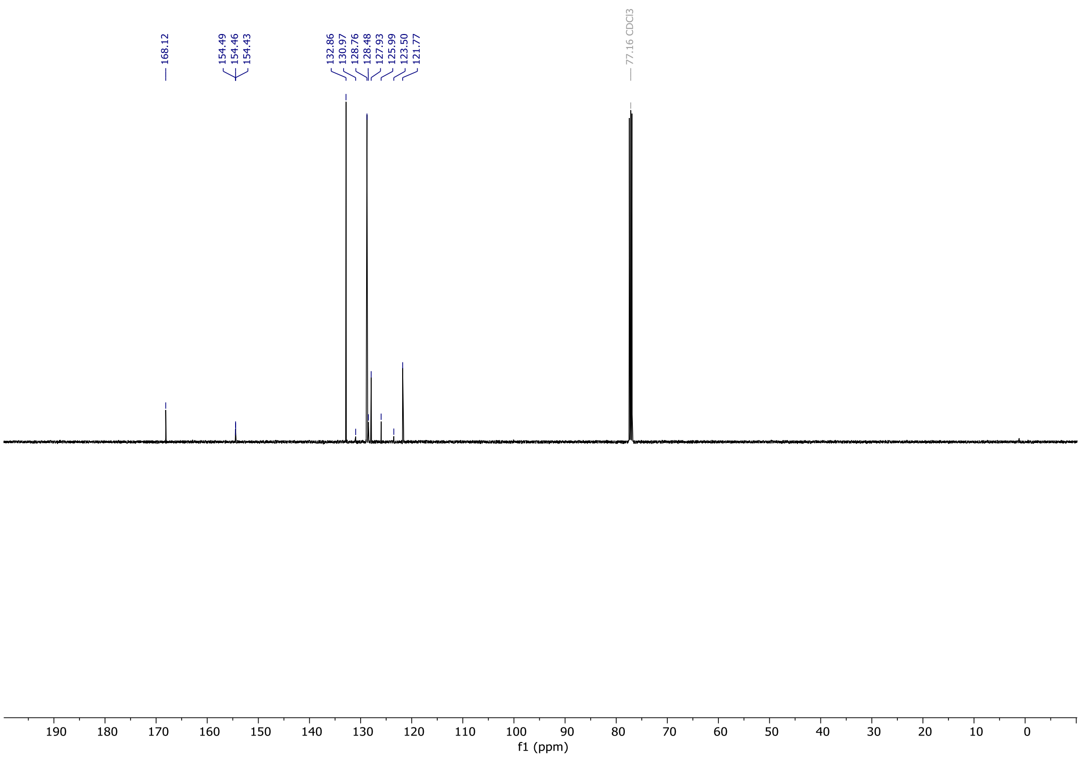

Supplementary Figure 54. ^{13}C NMR spectra of compound 3o. $CDCl_3$ solvent and 125 MHz frequency.

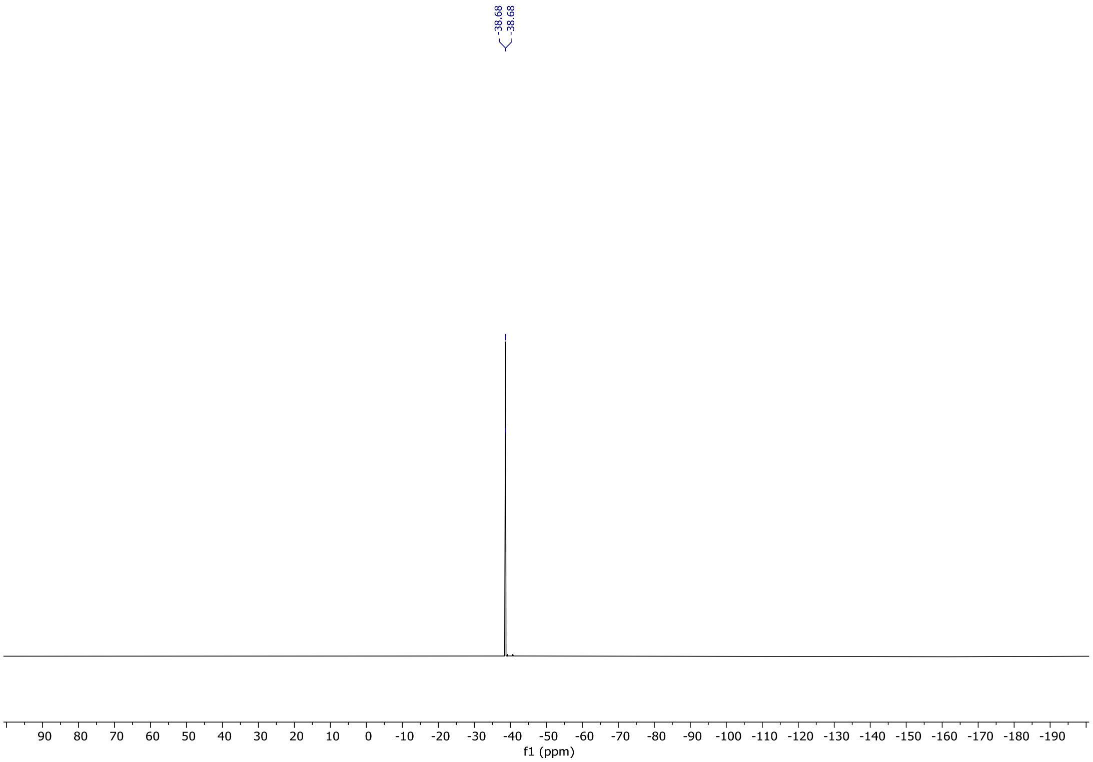

Supplementary Figure 55. ^{19}F NMR spectra of compound 3o. $CDCl_3$ solvent and 375 MHz frequency.

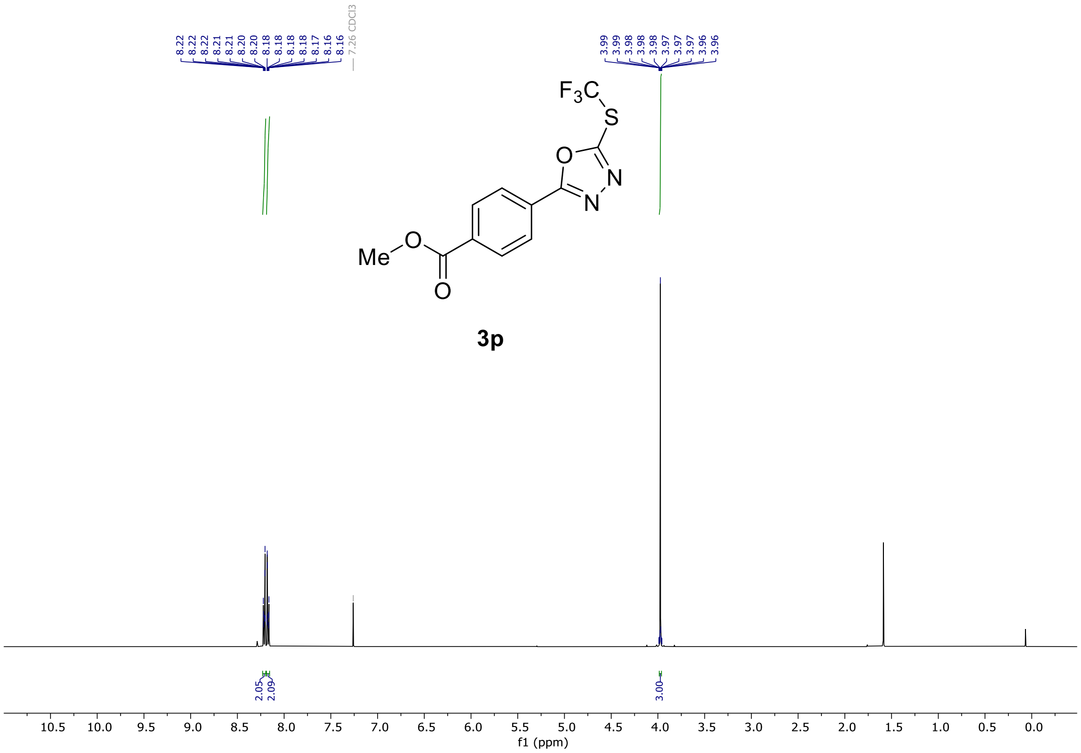

Supplementary Figure 56. ¹H NMR spectra of compound 3p. $CDCl_3$ solvent and 500 MHz frequency.

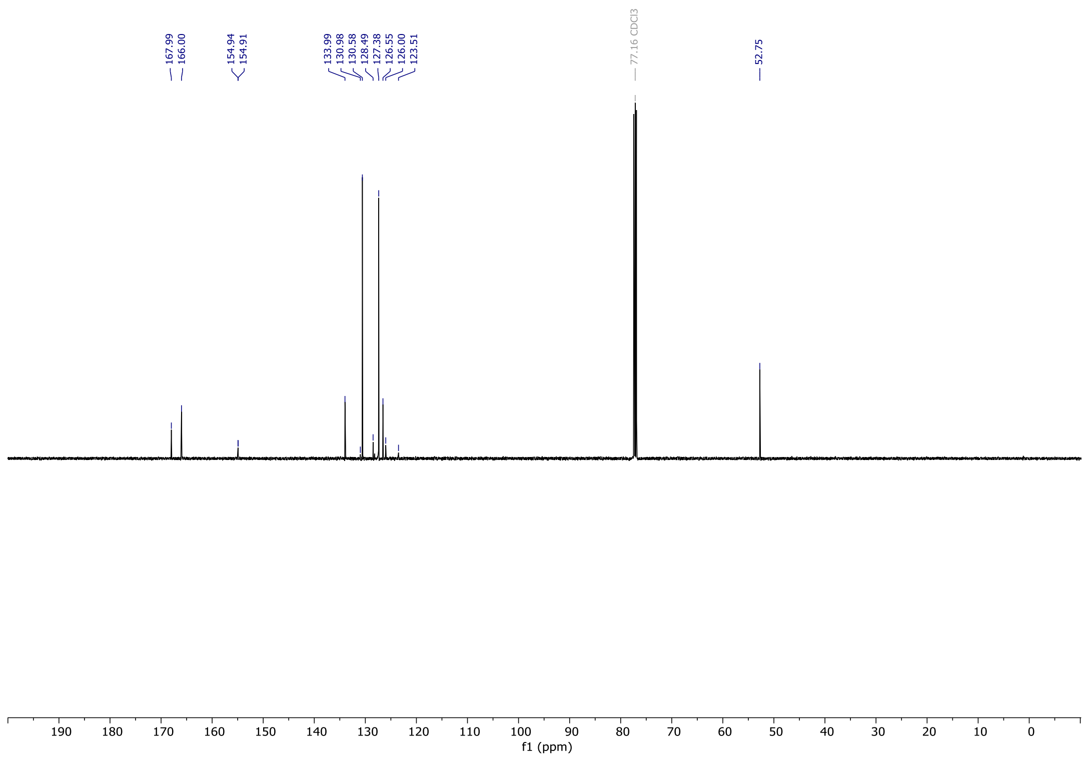

Supplementary Figure 57. ^{13}C NMR spectra of compound 3p. $CDCl_3$ solvent and 125 MHz frequency.

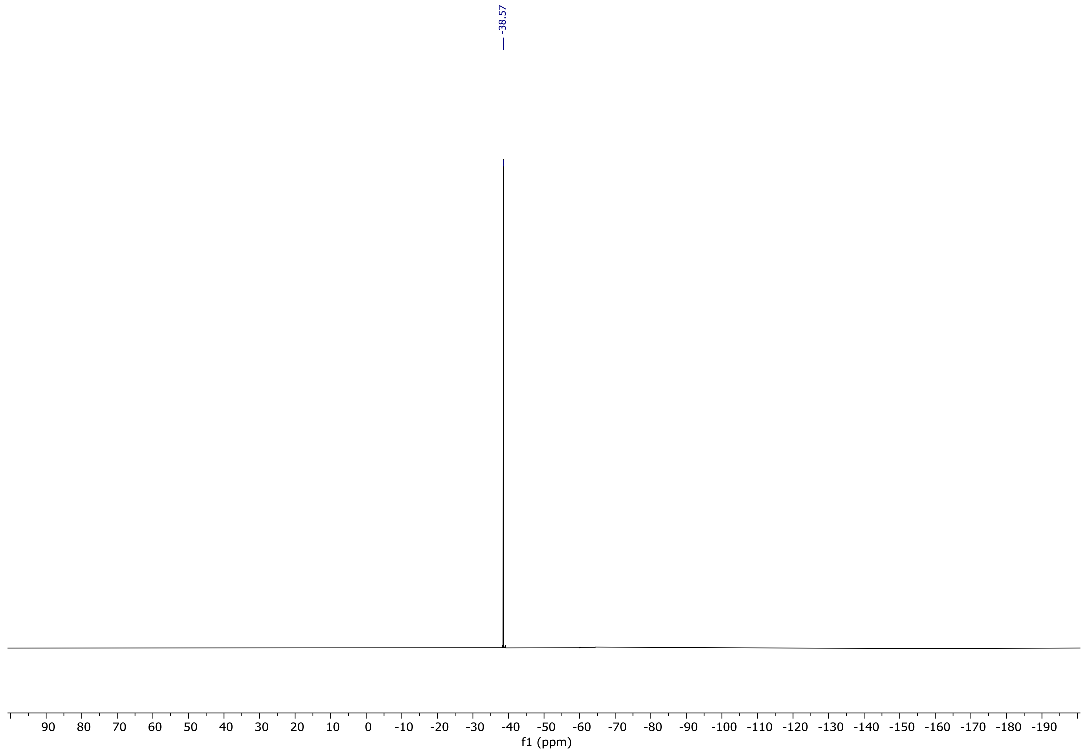

Supplementary Figure 58. ^{19}F NMR spectra of compound 3p. $CDCl_3$ solvent and 375 MHz frequency.

SUPPLEMENTARY TABLES

Supplementary Table 1. Solvent and flow rate gradients for HPLC analysis of *C. elegans* lysates.

Time	Flow Rate (mL/min)	% Solvent A 4.9:95:0.1 (ACN:H2O:Acetic Acid)	% Solvent B 95:4.9:0.1 (ACN:H2O:Acetic Acid)
0.00	1.3	85.0	15.0
4.49	1.3	43.5	56.5
4.50	1.0	43.5	56.5
5.54	1.0	42.0	58.0
6.54	2.0	30.0	70.0
8.04	3.0	0.0	100.0
8.34	3.0	0.0	100.0
8.35	2.0	85.0	15.0
8.65	2.0	85.0	15.0

Supplementary Table 2. Solvent and flow rate gradients for LC-MS analysis of nematode and yeast lysates

Time	Flow Rate (mL/min)	% Solvent A H2O, 0.1% Formic Acid	% Solvent B ACN, 0.1% Formic Acid
0.00	0.35	95.0	5.0
1.00	0.35	95.0	5.0
21.00	0.35	5.0	95.0
21.01	0.35	0.0	100.0
26.00	0.35	0.0	100.0
26.01	0.35	95.0	5.0
31.00	0.35	95.0	5.0

Supplementary Table 3. Exact mass and accurate mass values for cyprocide-B, cyprocide-E, and metabolites.

Organism	Analyte	Ionic Formula	Ion Species	Exact Mass	Accurate Mass	+/- (ppm)
C. elegans	cyprocide-B	$C_{10}H_{10}ClN_2OS$	$(M+H)^+$	241.0197	241.0193	-1.659615
C. elegans	sulfoxide	$C_{10}H_{10}ClN_2O_2S$	$(M+H)^+$	257.0146	257.0141	-1.945415
C. elegans	GSH-conjugate	$C_{18}H_{21}ClN_5O_7S$	$(M+H)^+$	486.0845	486.0847	0.411451
C. elegans	γ-Glu-Cys-conjugate	$C_{16}H_{18}ClN_4O_6S$	$(M+H)^+$	429.0630	429.0628	-0.466132
C. elegans	Cys-Gly-conjugate	$C_{13}H_{14}ClN_4O_4S$	$(M+H)^+$	357.0419	357.0423	1.120317
C. elegans	Cys-conjugate	$C_{11}H_{11}ClN_3O_3S$	$(M+H)^+$	300.0204	300.0210	1.999864
S. cerevisiae + EV	cyprocide-B	$C_{10}H_{10}ClN_2OS$	$(M+H)^+$	241.0197	241.0212	6.223558
S. cerevisiae + EV	sulfoxide	$C_{10}H_{10}ClN_2O_2S$	$(M+H)^+$	257.0146	not detected	not detected
S. cerevisiae + EV	GSH-conjugate	$C_{18}H_{21}ClN_5O_7S$	$(M+H)^+$	486.0845	not detected	not detected
S. cerevisiae + EV	γ-Glu-Cys-conjugate	$C_{16}H_{18}ClN_4O_6S$	$(M+H)^+$	429.0630	not detected	not detected
S. cerevisiae + EV	Cys-Gly-conjugate	$C_{13}H_{14}ClN_4O_4S$	$(M+H)^+$	357.0419	not detected	not detected
S. cerevisiae + EV	Cys-conjugate	$C_{11}H_{11}ClN_3O_3S$	$(M+H)^+$	300.0204	not detected	not detected
S. cerevisiae + CYP-35D1	cyprocide-B	$C_{10}H_{10}ClN_2OS$	$(M+H)^+$	241.0197	241.0207	4.149038
S. cerevisiae + CYP-35D1	sulfoxide	$C_{10}H_{10}ClN_2O_2S$	$(M+H)^+$	257.0146	not detected	not detected
S. cerevisiae + CYP-35D1	GSH-conjugate	$C_{18}H_{21}ClN_5O_7S$	$(M+H)^+$	486.0845	486.0865	4.114511
S. cerevisiae + CYP-35D1	γ-Glu-Cys-conjugate	$C_{16}H_{18}ClN_4O_6S$	$(M+H)^+$	429.0630	429.0658	6.525848
S. cerevisiae + CYP-35D1	Cys-Gly-conjugate	$C_{13}H_{14}ClN_4O_4S$	$(M+H)^+$	357.0419	357.0436	4.761346
S. cerevisiae + CYP-35D1	Cys-conjugate	$C_{11}H_{11}ClN_3O_3S$	$(M+H)^+$	300.0204	300.0223	6.332903
S. cerevisiae + CYP4731A3	cyprocide-B	$C_{10}H_{10}ClN_2OS$	$(M+H)^+$	241.0197	241.0207	4.149038
S. cerevisiae + CYP4731A3	sulfoxide	$C_{10}H_{10}ClN_2O_2S$	$(M+H)^+$	257.0146	not detected	not detected
S. cerevisiae + CYP4731A3	GSH-conjugate	$C_{18}H_{21}ClN_5O_7S$	$(M+H)^+$	486.0845	486.0871	5.348864
S. cerevisiae + CYP4731A3	γ-Glu-Cys-conjugate	$C_{16}H_{18}ClN_4O_6S$	$(M+H)^+$	429.0630	429.0664	7.924244
S. cerevisiae + CYP4731A3	Cys-Gly-conjugate	$C_{13}H_{14}ClN_4O_4S$	$(M+H)^+$	357.0419	357.0439	5.601583
S. cerevisiae + CYP4731A3	Cys-conjugate	$C_{11}H_{11}ClN_3O_3S$	$(M+H)^+$	300.0204	300.0226	7.332835
D. dipsaci	cyprocide-B	$C_{10}H_{10}ClN_2OS$	$(M+H)^+$	241.0197	241.0177	-8.298077
D. dipsaci	sulfoxide	$C_{10}H_{10}ClN_2O_2S$	$(M+H)^+$	257.0146	not detected	not detected
D. dipsaci	GSH-conjugate	$C_{18}H_{21}ClN_5O_7S$	$(M+H)^+$	486.0845	486.0809	-7.40612
D. dipsaci	γ-Glu-Cys-conjugate	$C_{16}H_{18}ClN_4O_6S$	$(M+H)^+$	429.0630	429.0593	-8.670055
D. dipsaci	Cys-Gly-conjugate	$C_{13}H_{14}ClN_4O_4S$	$(M+H)^+$	357.0419	357.0390	-8.122296
D. dipsaci	Cys-conjugate	$C_{11}H_{11}ClN_3O_3S$	$(M+H)^+$	300.0204	300.0182	-7.332835
P. penetrans	cyprocide-B	$C_{10}H_{10}ClN_2OS$	$(M+H)^+$	241.0197	241.0176	-8.712981
P. penetrans	sulfoxide	$C_{10}H_{10}ClN_2O_2S$	$(M+H)^+$	257.0146	257.0142	-1.556332
P. penetrans	GSH-conjugate	$C_{18}H_{21}ClN_5O_7S$	$(M+H)^+$	486.0845	486.0805	-8.229022
P. penetrans	γ-Glu-Cys-conjugate	$C_{16}H_{18}ClN_4O_6S$	$(M+H)^+$	429.0630	429.0594	-8.390376
P. penetrans	Cys-Gly-conjugate	$C_{13}H_{14}ClN_4O_4S$	$(M+H)^+$	357.0419	357.0396	-6.413813
P. penetrans	Cys-conjugate	$C_{11}H_{11}ClN_3O_3S$	$(M+H)^+$	300.0204	300.0187	-5.666281
M. hapla	cyprocide-B	$C_{10}H_{10}ClN_2OS$	$(M+H)^+$	241.0197	241.0174	-9.542788
M. hapla	sulfoxide	$C_{10}H_{10}ClN_2O_2S$	$(M+H)^+$	257.0146	not detected	not detected
M. hapla	GSH-conjugate	$C_{18}H_{21}ClN_5O_7S$	$(M+H)^+$	486.0845	486.0806	-8.023296
M. hapla	γ-Glu-Cys-conjugate	$C_{16}H_{18}ClN_4O_6S$	$(M+H)^+$	429.0630	429.0589	-9.555706
M. hapla	Cys-Gly-conjugate	$C_{13}H_{14}ClN_4O_4S$	$(M+H)^+$	357.0419	357.0383	-10.08285
M. hapla	Cys-conjugate	$C_{11}H_{11}ClN_3O_3S$	$(M+H)^+$	300.0204	300.0175	-9.666009

System	Analyte	Ionic Formula	Ion Species	Exact Mass	Accurate Mass	+/- (ppm)
S. cerevisiae + EV	cyprocide-E	$C_{11}H_{10}BrN_2OS$	$(M+H)^+$	296.9692	296.9707	5.051029
S. cerevisiae + EV	sulfoxide	$C_{11}H_{10}BrN_2O_2S$	$(M+H)^+$	312.9641	not detected	not detected
S. cerevisiae + EV	GSH-conjugate	$C_{18}H_{21}BrN_5O_7S$	$(M+H)^+$	530.0340	not detected	not detected
S. cerevisiae + EV	γ-Glu-Cys-conjugate	$C_{16}H_{18}BrN_4O_6S$	$(M+H)^+$	473.0125	not detected	not detected
S. cerevisiae + EV	Cys-Gly-conjugate	$C_{13}H_{14}BrN_4O_4S$	$(M+H)^+$	400.9914	not detected	not detected
S. cerevisiae + EV	Cys-conjugate	$C_{11}H_{11}BrN_3O_3S$	$(M+H)^+$	343.9699	not detected	not detected
S. cerevisiae + CYP4731A3	cyprocide-E	$C_{11}H_{10}BrN_2OS$	$(M+H)^+$	296.9692	296.9716	8.081646
S. cerevisiae + CYP4731A3	sulfoxide	$C_{11}H_{10}BrN_2O_2S$	$(M+H)^+$	312.9641	not detected	not detected
S. cerevisiae + CYP4731A3	GSH-conjugate	$C_{18}H_{21}BrN_5O_7S$	$(M+H)^+$	530.0340	530.0371	5.848681
S. cerevisiae + CYP4731A3	γ-Glu-Cys-conjugate	$C_{16}H_{18}BrN_4O_6S$	$(M+H)^+$	473.0125	473.0151	5.496683
S. cerevisiae + CYP4731A3	Cys-Gly-conjugate	$C_{13}H_{14}BrN_4O_4S$	$(M+H)^+$	400.9914	not detected	not detected
S. cerevisiae + CYP4731A3	Cys-conjugate	$C_{11}H_{11}BrN_3O_3S$	$(M+H)^+$	343.9699	343.9720	6.105185

Accurate mass was not determined for those analytes with an abundance below the threshold of 1 x 10^3 counts which were considered 'not detected'

SUPPLEMENTARY REFERENCES

1. Powers, D. C. *et al.* Connecting binuclear Pd(III) and mononuclear Pd(IV) chemistry by Pd-Pd bond cleavage. *J. Am. Chem. Soc.* **134**, 12002–12009 (2012).
2. Soleiman-Beigi, M., Alikarami, M. & Hosseinzadeh, T. One-pot Synthesis of 2-Alkylthio-1,3,4-oxadiazole and Bis-(1,3,4-oxadiazole-2-yl)thio alkyl Derivatives from Acid Hydrazides and CS2. *Asian J. Chem.* **25**, 4939–4942 (2013).
3. Yan, X. *et al.* Propylene oxide assisted one-pot, tandem synthesis of substituted-1,3,4-oxadiazole-2(3H)-ones in water. *Tetrahedron* **68**, 7978–7983 (2012).
4. Xu, W.-M. *et al.* Inhibition of Tobacco Bacterial Wilt with Sulfone Derivatives Containing an 1,3,4-Oxadiazole Moiety. *J. Agric. Food Chem.* **60**, 1036–1041 (2012).
