# Supplementary material for: Cyprocide selectively kills nematodes via cytochrome P450 bioactivation
Source: Nat Commun. 2024 Jul 2;15:5529. doi: 10.1038/s41467-024-49738-4 (PMC11219838; doi:10.1038/s41467-024-49738-4)
Supplement: Supplementary file 3 — Description of Additional Supplementary Files [file 41467_2024_49738_MOESM3_ESM.pdf]

Description of Additional Supplementary Files

Supplementary Data 1. Details on the POR disruption survey to identify bioactivated worm-active molecules (wactives).

Supplementary Data 2. DODA chemical library information and detailed screen results identifying molecules that kill C. elegans and plant-parasitic nematodes.

Supplementary Data 3. Cytochrome P450 cDNA sequences codon optimized for S. cerevisiae expression.
