# Supplementary material for: Cyprocide selectively kills nematodes via cytochrome P450 bioactivation
Source: Nat Commun. 2024 Jul 2;15:5529. doi: 10.1038/s41467-024-49738-4 (PMC11219838; doi:10.1038/s41467-024-49738-4)
Supplement: Supplementary file 7 — Reporting Summary [file 41467_2024_49738_MOESM7_ESM.pdf]

nature portfolio

Corresponding author(s): Peter Roy

Last updated by author(s): Jun 12, 2024

Reporting Summary

Nature Portfolio wishes to improve the reproducibility of the work that we publish. This form provides structure for consistency and transparency in reporting. For further information on Nature Portfolio policies, see our Editorial Policies and the Editorial Policy Checklist.

Statistics

For all statistical analyses, confirm that the following items are present in the figure legend, table legend, main text, or Methods section.

n/a	Confirmed	
☐	☒	The exact sample size (*n*) for each experimental group/condition, given as a discrete number and unit of measurement
☐	☒	A statement on whether measurements were taken from distinct samples or whether the same sample was measured repeatedly
☐	☒	The statistical test(s) used AND whether they are one- or two-sided<br>*Only common tests should be described solely by name; describe more complex techniques in the Methods section.*
☒	☐	A description of all covariates tested
☒	☐	A description of any assumptions or corrections, such as tests of normality and adjustment for multiple comparisons
☐	☒	A full description of the statistical parameters including central tendency (e.g. means) or other basic estimates (e.g. regression coefficient) AND variation (e.g. standard deviation) or associated estimates of uncertainty (e.g. confidence intervals)
☐	☒	For null hypothesis testing, the test statistic (e.g. *F*, *t*, *r*) with confidence intervals, effect sizes, degrees of freedom and *P* value noted<br>*Give P values as exact values whenever suitable.*
☒	☐	For Bayesian analysis, information on the choice of priors and Markov chain Monte Carlo settings
☒	☐	For hierarchical and complex designs, identification of the appropriate level for tests and full reporting of outcomes
☒	☐	Estimates of effect sizes (e.g. Cohen's *d*, Pearson's *r*), indicating how they were calculated

Our web collection on statistics for biologists contains articles on many of the points above.

Software and code

Policy information about availability of computer code

Data collection	HPLC-DAD data was collected using HP Chemstation for LC 3D (v. A.10.02). LC-MS data was collected using Agilent MassHunter Acquisition Software (v. 11.0.1).
Data analysis	Cheminformatic analysis was performed using the Strip-it command line tool (v. 1.0.2), the ChemmineR Cheminformatic Toolkit for R package (v. 3.50.0), OpenBabel (v. 3.1.0) and Cytoscape (v. 3.9.1). HPLC-DAD analysis was performed using the HP Chemstation for LC 3D (v. A.10.02), Microsoft Excel for Mac (v. 16.72), GraphPad Prism (v. 10.0), and a python script for generating heatmaps of HPLC chromatograms (https://doi.org/10.5281/zenodo.7731172). LC-MS data analysis was performed using the Agilent MassHunter Qualitative Analysis Software (v.10.0) and GraphPad Prism (v. 10.0). All other data were analyzed using Microsoft Excel for Mac (v. 16.72) and/or GraphPad Prism (v. 10.0). Chemical structures were drawn using ChemDraw (v. 22.0.0).

For manuscripts utilizing custom algorithms or software that are central to the research but not yet described in published literature, software must be made available to editors and reviewers. We strongly encourage code deposition in a community repository (e.g. GitHub). See the Nature Portfolio guidelines for submitting code & software for further information.

Data

Policy information about availability of data

All manuscripts must include a data availability statement. This statement should provide the following information, where applicable:

- Accession codes, unique identifiers, or web links for publicly available datasets
- A description of any restrictions on data availability
- For clinical datasets or third party data, please ensure that the statement adheres to our policy

All data are available in the main text, the Supplementary Information and/or in the Source Data file. Source data are provided with this paper. All data are available from the corresponding author upon request.

Research involving human participants, their data, or biological material

Policy information about studies with human participants or human data. See also policy information about sex, gender (identity/presentation), and sexual orientation and race, ethnicity and racism.

Reporting on sex and gender	Our manuscript focuses on model systems, so these issues are not relevant.
Reporting on race, ethnicity, or other socially relevant groupings	Our manuscript focuses on model systems, so these issues are not relevant.
Population characteristics	Our manuscript focuses on model systems, so these issues are not relevant.
Recruitment	Our manuscript focuses on model systems, so these issues are not relevant.
Ethics oversight	Our manuscript focuses on model systems, so these issues are not relevant.

Note that full information on the approval of the study protocol must also be provided in the manuscript.

Field-specific reporting

Please select the one below that is the best fit for your research. If you are not sure, read the appropriate sections before making your selection.

[x] Life sciences [ ] Behavioural & social sciences [ ] Ecological, evolutionary & environmental sciences

For a reference copy of the document with all sections, see nature.com/documents/nr-reporting-summary-flat.pdf

Life sciences study design

All studies must disclose on these points even when the disclosure is negative.

Sample size	No statistical methods were used to predetermine sample size. Typically at least three independent biological repeats were performed with multiple technical repeats within each biological replicate. Sample sizes were chosen that would allow for error calculations and significance testing while maintaining feasibility of data acquisition and analysis. The specific number of replicates for each experiment are indicated in the Methods section and in the figure legends.
Data exclusions	No data was excluded from the trials.
Replication	Statistical analyses were performed as described in the manuscript. At least three independent replicates were performed for nearly all experiments and variability was relatively low between replicates. All attempts at replication were successful - there were no instances where replicates were attempted and results could not be reproduced.
Randomization	In all experiments, test subjects (organisms, cells, etc.) were chosen at random from a large population for experimentation. There was no attempt at cherry picking or biasing the samples.
Blinding	Experiments represented by Figures 1A and 1C had so many samples that they should be considered blinded. Other experiments are quantitative in nature and not subjected to bias in any obvious way.

Reporting for specific materials, systems and methods

We require information from authors about some types of materials, experimental systems and methods used in many studies. Here, indicate whether each material, system or method listed is relevant to your study. If you are not sure if a list item applies to your research, read the appropriate section before selecting a response.

Materials & experimental systems

n/a	Involved in the study
☒	☐ Antibodies
☐	☒ Eukaryotic cell lines
☒	☐ Palaeontology and archaeology
☐	☒ Animals and other organisms
☒	☐ Clinical data
☒	☐ Dual use research of concern
☐	☒ Plants

Methods

n/a	Involved in the study
☒	☐ ChIP-seq
☒	☐ Flow cytometry
☒	☐ MRI-based neuroimaging

Eukaryotic cell lines

Policy information about cell lines and Sex and Gender in Research

Cell line source(s)	HEK293 cells were purchased from ThermoFisher (Cat# R71007). HepG2 cells were purchased from ATCC (Cat: HB-8065)
Authentication	HEK293 cells were authenticated by STR analysis at the Toronto Hospital for Sick Children authentication facility. HepG2 cell line was not authenticated as the specific tissue of origin was not imperative to the validity of the reported results.
Mycoplasma contamination	HEK293 cells are tested regularly for mycoplasma contamination using the eMyco kit from FroggaBio (Cat. # 25239). Tests performed a short time before experimentation confirmed that the cell line is negative for mycoplasma contamination. HepG2 cell line tested negative for mycoplasma contamination via PCR.
Commonly misidentified lines (See ICLAC register)	HEK293 and HepG2 are not on the list of commonly misidentified cell lines.

Animals and other research organisms

Policy information about studies involving animals; ARRIVE guidelines recommended for reporting animal research, and Sex and Gender in Research

Laboratory animals	The D. rerio (zebrafish) assays used AB wild-type fish at 3 days-post-fertilization.
Wild animals	N/A
Reporting on sex	For our drosophila experiments equal numbers of male and female flies were used. Sex is not a consideration with any of the other model systems that we have used, including zebrafish, where sex is undetermined until adulthood.
Field-collected samples	N/A
Ethics oversight	The D. rerio (zebrafish) ethics protocol is 65697, approved by the Animal Care Committee at The Hospital for Sick Children, Canada

Note that full information on the approval of the study protocol must also be provided in the manuscript.

Dual use research of concern

Policy information about dual use research of concern

Hazards

Could the accidental, deliberate or reckless misuse of agents or technologies generated in the work, or the application of information presented in the manuscript, pose a threat to:

No	Yes	
☒	☐	Public health
☒	☐	National security
☒	☐	Crops and/or livestock
☒	☐	Ecosystems
☒	☐	Any other significant area

Experiments of concern

Does the work involve any of these experiments of concern:

No	Yes	
☒	☐	Demonstrate how to render a vaccine ineffective
☒	☐	Confer resistance to therapeutically useful antibiotics or antiviral agents
☒	☐	Enhance the virulence of a pathogen or render a nonpathogen virulent
☒	☐	Increase transmissibility of a pathogen
☒	☐	Alter the host range of a pathogen
☒	☐	Enable evasion of diagnostic/detection modalities
☒	☐	Enable the weaponization of a biological agent or toxin
☒	☐	Any other potentially harmful combination of experiments and agents

Plants

Seed stocks	Tomato seeds (Solanum lycopersicum 'Rutgers') were originally purchased from Burpee Gardening, Warminster, PA.
Novel plant genotypes	No novel plant genotypes were used.
Authentication	No authentication was performed.
